# Tuning the anion binding properties of lanthanide receptors to discriminate nucleoside phosphates in a sensing array†

**DOI:** 10.1039/d0sc00343c

**Published:** 2020-03-11

**Authors:** Sarah H. Hewitt, Georgina Macey, Romain Mailhot, Mark R. J. Elsegood, Fernanda Duarte, Alan M. Kenwright, Stephen J. Butler

**Affiliations:** Department of Chemistry, Loughborough University Epinal Way Loughborough LE11 3TU UK S.J.Butler@lboro.ac.uk; Chemistry Research Laboratory, University of Oxford 12 Mansfield Road Oxford OX1 3TA UK; Department of Chemistry, Durham University South Road Durham DH1 3LE UK

## Abstract

The development of synthetic receptors for the selective binding and discrimination of anions in water requires an understanding of how anions interact with these synthetic receptors. Molecules designed to differentiate nucleoside phosphate anions (*e.g.* ATP, ADP, GTP, GDP, UDP) under physiological conditions could underpin exciting new sensing tools for biomedical research and drug discovery, but it is very challenging due to the similarities in anion structure, size and charge. We present a series of lanthanide-based anion receptors and establish key structural elements that impact on nucleoside phosphate anion binding and sensing. Structural evidence of anion binding using X-ray crystallographic and NMR data, supported by DFT calculations indicate the binding modes between the lanthanide complexes and certain phosphoanions, revealing a bidentate (α-, γ-) binding mode to ATP. We further use four of the receptors to allow discrimination of eight nucleoside phosphate anions in the first array-based assay using lanthanide complexes, taking advantage of the multiple emission bands and long emission lifetimes associated with luminescent lanthanide complexes.

## Abstract 2

Discrimination of nucleoside phosphate anions in the first supramolecular sensing array using luminescent lanthanide complexes.

## Graphical abstract

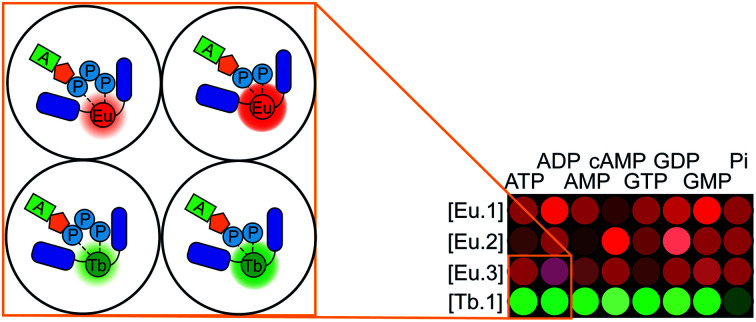

## Introduction

A central challenge in the field of supramolecular anion receptor chemistry is the design and synthesis of host molecules that bind selectively to a target anion in water.^1,2^ A second challenge involves translating the binding event into a measurable (*e.g.* luminescent or colorimetric) signal.^3,4^ Significant research effort is focussed on developing synthetic receptors for nucleoside phosphate anions, due to the critical roles they play in biological processes, including energy transduction, phosphorylation, cellular signalling and DNA synthesis.^5,6^ The creation of receptors capable of binding and differentiating nucleoside phosphate anions (*e.g.* ATP, ADP, AMP, GTP, GDP, UDP) under physiological conditions could underpin exciting new sensing tools for biomedical research and drug discovery,^7^ but it is very challenging due to the similarities in anion structure, size and charge. Additionally, the high hydration energies of polyphosphate anions mean that a synthetic receptor must compete with water molecules for negatively charged phosphate groups. Consequently, examples of synthetic receptors that exhibit high affinity and selectivity for specific nucleoside phosphate anions are rare.^8,9^

Molecular receptors that utilise strong electrostatic or metal–ligand interactions are required to overcome the high hydration energies of phosphate anions in water. A series of dinuclear Zn(ii) complexes have been developed, which exhibit strong binding to a range of polyphosphate anions (*e.g.*, ATP, ADP, pyrophosphate),^10,11^ and phosphorylated peptides in water,^12,13^ where the anion acts as bridging ligand for the two zinc(ii) centres. However, the majority of these receptors bind di- and triphosphate anions (ATP, ADP, PPi) with similar affinities and produce similar fluorescence responses. Improvements in anion selectivity have been achieved by utilising a combination of metal–ligand and hydrogen bonding interactions in the receptor design.^14–17^

Receptors based on stable lanthanide complexes offer scope for the design of selective anion receptors, in which the affinity and selectivity can be modulated by variations in the ligand structure and its conformational flexibility, steric hindrance at the metal centre, and the overall charge of the complex.^18–20^ Macrocyclic heptadentate ligands have been designed to prepare emissive europium(iii) and terbium(iii) complexes with one or two available anion binding sites, occupied by water molecules in aqueous solution.^21,22^ Anion binding may be signaled by variations in luminescence intensity, spectral shape and lifetime, caused by changes in the Ln(iii) coordination environment and displacement of quenching water molecules. This strategy has been utilised to develop receptors for anions including phosphate,^23–25^ bicarbonate,^26,27^ fluoride,^28–30^ and lactate.^31^ Such Ln(iii) complexes are kinetically stable, avoiding complications arising from metal ion dissociation upon anion binding.

Owing to the Laporte forbidden nature of f–f orbital transitions, the luminescence of Eu(iii) and Tb(iii) complexes is sufficiently long-lived to be exploited for anion sensing in complex biological media (*e.g.* blood serum),^32^ by using time-gating methods to distinguish the lanthanide-centred emission from the short-lived autofluorescence of biomolecules.^33–35^ Time-gated (or time-resolved) luminescence measurements offer enhanced signal-to-noise and very low limits of anion detection.

**Fig. 1:**
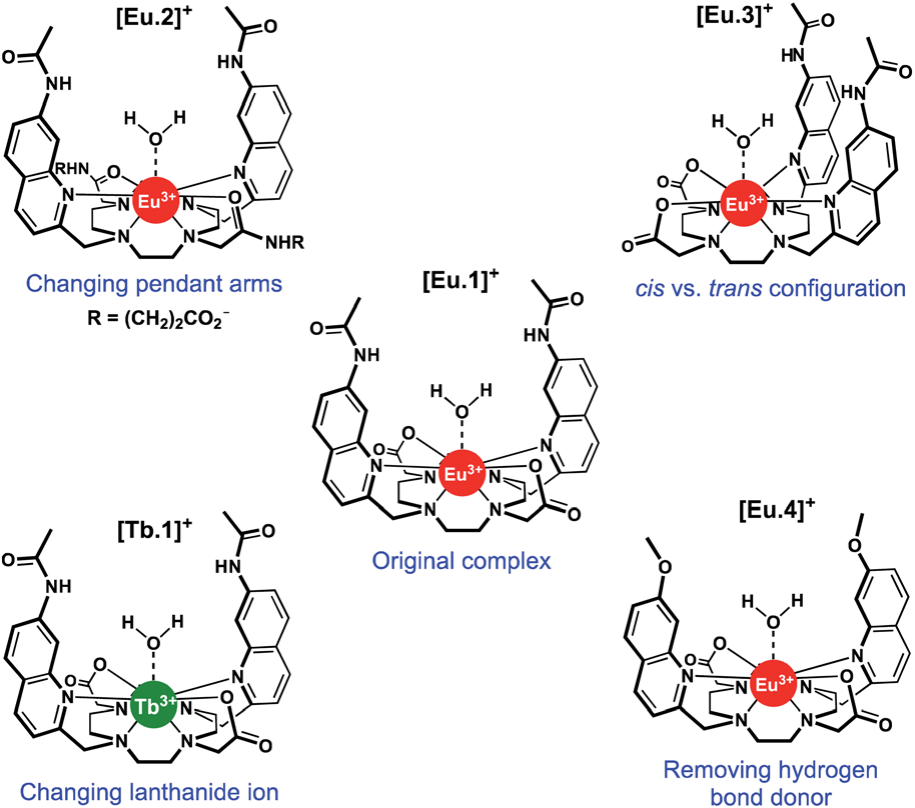
Structures of Ln complexes [**Eu.1–4**]^+^ and [**Tb.1**]^+^.

The design and synthesis of lanthanide-based receptors that provide a binding site to accommodate larger polyphosphate anions over smaller anions (*e.g.* phosphate, bicarbonate) remains a significant challenge. We recently reported [**Eu.1**]^+^ (Fig. 1), based on an octadentate ligand bearing two *trans*-related quinoline groups, as an effective receptor for polyphosphate anions.^36^ Lanthanide complexes of octadentate ligands (based on DOTA) generally provide insufficient space for the reversible coordination of larger anions. However, we showed that [**Eu.1**]^+^ is able to bind reversibly to ATP and ADP in aqueous solution, giving rise to distinctly different Eu(iii) emission spectra in the presence of physiological (millimolar) levels of MgCl_2_. The ability of [**Eu.1**]^+^ to differentiate ATP and ADP was utilised to monitor kinase catalysed phosphorylation reactions in real-time, by reporting on the conversion of ATP to ADP. Subsequently, the Eu(iii) receptor was developed into a miniaturised supramolecular assay for monitoring a range of pharmaceutically important enzymes that generate nucleoside phosphate anions (*e.g.*, kinases, glycosyltransferases, and phosphodiesterases).^37^

In this work, we have evaluated the anion binding and sensing properties of [**Eu.1**]^+^ and four structurally related lanthanide complexes [**Eu.2–4**]^+^ and [**Tb.1**]^+^ (Fig. 1). We have elucidated the binding mode of ATP to [**Eu.1**]^+^ and identified key structural elements required for polyphosphate anion recognition, using a combination of NMR spectroscopy, X-ray diffraction, and DFT calculations. It is possible to influence the affinity, selectivity, and emission response of this class of Ln(iii)-based receptors towards phosphoanion guests by modulating the hydrogen bond donor ability of the two quinoline groups, their relative positions on the macrocyclic scaffold, and the charge density of the Eu(iii) ion, by varying the nature of the ‘non-binding’ Eu(iii) donor groups. Finally, we demonstrate the utility of this new family of lanthanide complexes to discriminate ten nucleoside phosphate anions (ATP, ADP, AMP, GTP, GDP, GMP, UMP, CMP, cAMP, Pi) using an array approach in aqueous solution.

## Results and discussion

### Synthesis and photophysical analysis of Ln complexes

Using the previously reported Eu(iii) complexes [**Eu.1**]^+^ and [**Eu.2**]^+^,^38^ and three new, structurally related lanthanide complexes [**Eu.3**]^+^, [**Eu.4**]^+^ and [**Tb.1**]^+^, we examined the impact of several of the designed structural features of [**Eu.1**]^+^ on anion affinity and selectivity, including: (1) the two carboxylate pendant arms, which are replaced with neutral carbonyl amide donors in [**Eu.2**]^+^; (2) the *trans* orientation of the quinoline groups on the macrocyclic ligand, compared to the *cis* configuration in [**Eu.3**]^+^; (3) the presence of hydrogen bond donor groups, which are replaced with methoxy groups in [**Eu.4**]^+^ and; (4) the lanthanide ion, by comparing with [**Tb.1**]^+^.

Details of the synthesis and characterisation of complexes [**Eu.3**]^+^ and [**Eu.4**]^+^ and [**Tb.1**]^+^ are provided in the ESI.† Briefly, a cyclen derivative bearing either two *cis*- or *trans*-related secondary amines was reacted with an appropriately functionalised 2-methylquinoline mesylate ester in the presence of K_2_CO_3_, to give the protected macrocyclic ligand. The *tert*-butyl protecting groups were removed using trifluoroacetic acid, followed by the addition of one equivalent of LnCl_3_ (Ln = Eu or Tb) in a mixture of water/methanol at pH 7–8 to give the water soluble Eu(iii) complexes [**Eu.3**]^+^, [**Eu.4**]^+^ and [**Tb.1**]^+^, after purification by preparative reverse-phase HPLC.

Selected photophysical data for [**Ln.1–4**]^+^ are provided in Table 1. The lanthanide complexes display similar absorption spectra, each characterised by a broad band centred at approximately 330 nm. Each Eu(iii) complex displays characteristic emission bands in the red region of the visible spectrum (Fig. S1†), including Δ*J* = 0 (575–582 nm), Δ*J* = 1 (582–605 nm), Δ*J* = 2 (605–630 nm), and Δ*J* = 4 (680–705 nm), while [**Tb.1**]^+^ emits green light weakly, with characteristic emission bands for Δ*J* = 1 (475–510 nm), Δ*J* = 2 (530–565 nm), Δ*J* = 3 (575–605), and Δ*J* = 4 (605–630 nm).^41,42^ Additionally, [**Tb.1**]^+^ shows significant quinoline (ligand) fluorescence overlapping the terbium(iii)-centred emission. Tb(iii) complexes are well known to exhibit higher sensitivity to quenching by dissolved oxygen compared with analogous Eu(iii) complexes.^43,44^ Indeed, on bubbling nitrogen gas through samples of [**Tb.1**]^+^ and [**Eu.1**]^+^ in aqueous buffer, there is very little change in the emission spectra of [**Eu.1**]^+^, whereas [**Tb.1**]^+^ shows substantially increased luminescence (Fig. S2†).

**Table 1:** Photophysical data for complexes [**Ln.1–4**]^+^ (10 mM HEPES, pH 7.0)

Complex	*λ*_max_/nm	*ε*/mM^−1^ cm^−1^	*ϕ*_em_ a/%	*τ*(H_2_O)/ms	*τ*(D_2_O)/ms	*q* b
[**Eu.1**]^+^	332	12.5	7.0	0.48	1.39	1.2
[**Eu.2**]^+^	330	7.7	6.5	0.56	1.22	0.8
[**Eu.3**]^+^	328	14.0	9.6	0.46	0.99	1.0
[**Eu.4**]^+^	332	10.1	8.3	0.54	1.28	0.9
[**Tb.1**]^+^	332	4.7	n.d.	—	—	—

a Overall luminescence quantum yields were measured using a previously reported 8-benzyloxyquinoline functionalized DO3A Eu(iii) complex (*ϕ*_em_ = 6%),^39^ and rhodamine 101 in acidified ethanol, as standards. Quantum yields have an estimated maximum uncertainty of ±20%.

b Values of hydration state, *q* (±20%) were derived using the modified Horrocks equation.^40^

Complexes [**Eu.1**]^+^ and [**Eu.4**]^+^ have identical emission spectral form (Fig. S1†), indicating that they are conformationally identical, whereas [**Eu.1**]^+^, [**Eu.2**]^+^ and [**Eu.3**]^+^ have notably different emission spectra, arising from differences in Eu(iii) coordination environment, specifically the nature and relative positions of the appended nitrogen and oxygen donor groups. The quantum yields of the metal-centred luminescence of complexes [**Eu.1–4**]^+^ were determined to be in the range 7–10%, by indirect excitation *via* the quinoline antennae (Table 1). Emission lifetimes were found to be between 115–190% larger in D_2_O compared to H_2_O. The number of coordinated water molecules was determined to be one for each Eu(iii) complex.^40^

### Eu(iii) emission spectral changes upon phosphoanion binding

**Fig. 2:**
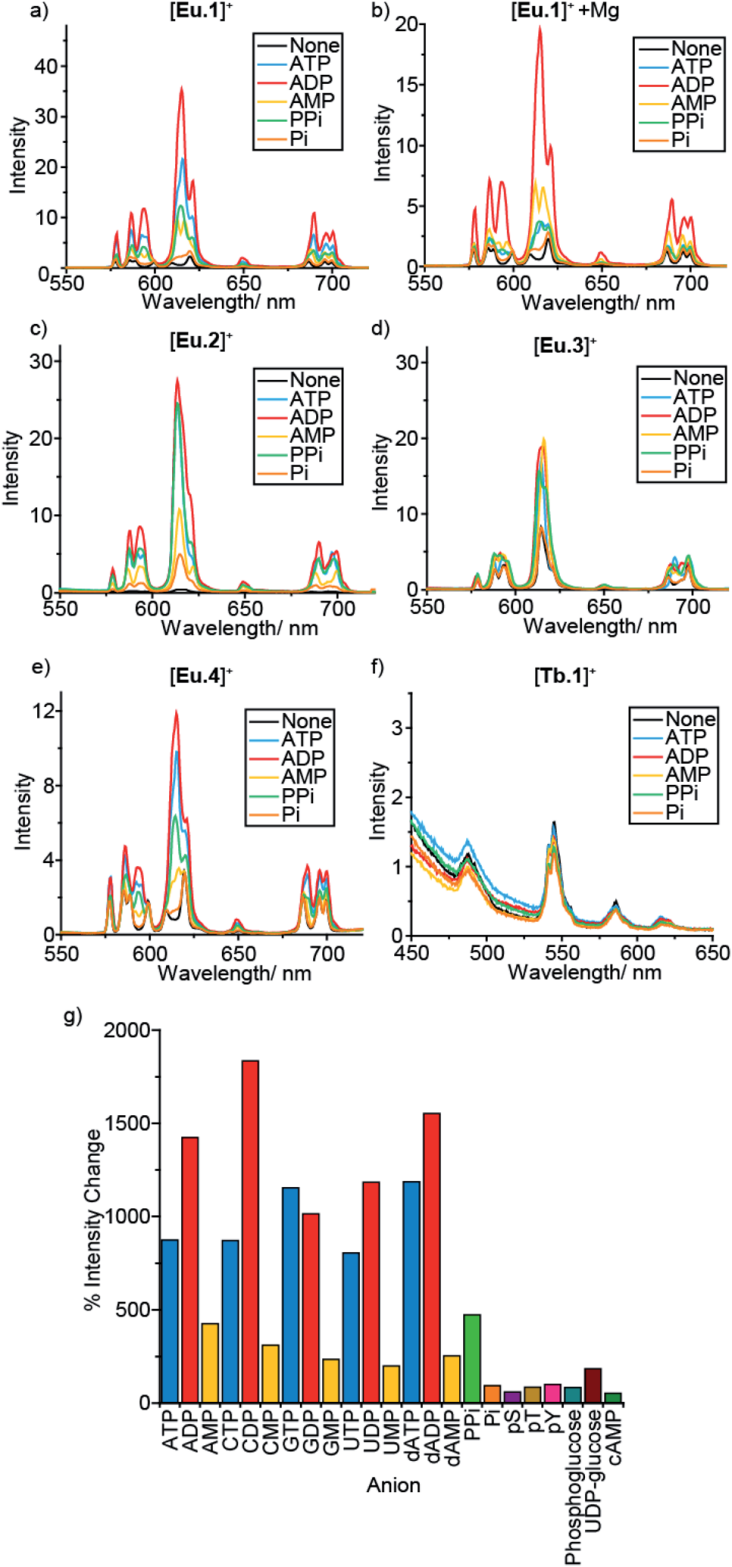
Effect of added anions (1 mM) on the emission spectra of complexes [**Ln.1–4**]^+^ in 10 mM HEPES, pH 7.0. (a) [**Eu.1**]^+^ (8 μM), (b) [**Eu.1**]^+^ (8 μM) in the presence of 5 mM MgCl_2_, (c) [**Eu.4**]^+^ (10 μM), (d) [**Eu.2**]^+^ (13 μM), (e) [**Eu.3**]^+^ (6 μM) and (f) [**Tb.1**]^+^ (15 μM). (g) Percentage change in emission intensity of the Δ*J* = 2 band of [**Eu.1**]^+^ (8 μM) on addition of a variety of phosphoanions (1 mM).

In a preliminary anion screening experiment, a range of nucleoside triphosphates (NTPs), nucleoside diphosphates (NDPs), nucleoside monophosphates (NMPs) and other mono-phosphorylated species, were added to the complexes in aqueous buffer (10 mM HEPES, pH 7.0) and the changes in emission spectra were recorded (Fig. 2 and S4–S16†). The addition of certain phosphate species to the Eu(iii) complexes caused significant enhancements in emission intensity, whereas the emission of [**Tb.1**]^+^ remained essentially unchanged, confirming the requirement of the Eu(iii) ion for anion sensing. Given that the emission bands of Tb(iii) are known to show only moderate sensitivity to changes in ligand environment, coupled with the sensitivity of [**Tb.1**]^+^ to dissolved oxygen, it is unsurprising that [**Tb.1**]^+^ is unable to sense phosphate anions in air-equilibrated aqueous solution.

For all four Eu(iii) complexes, two general trends can be observed from the anion screening. First, the addition of NTP and NDP anions enhances the luminescence of each Eu(iii) complex, due to displacement of a labile, quenching water molecule, verified by changes in luminescence lifetime for complexes [**Eu.1**]^+^, [**Eu.2**]^+^ and [**Eu.4**]^+^ with ATP and ADP (Table S10†). Second, adding mono-phosphorylated species (NMPs, cAMP, pY, pT, pS, Pi, phosphoglucose) or UDP–glucose causes only minor changes in emission intensity of the Eu(iii) complexes, indicating that di- or triphosphate groups are required for recognition.

For the individual Eu(iii) complexes, addition of any of the NTP anions (ATP, CTP, GTP, UTP) induces a similar emission response, as does any of the NDP or NMP anions. The nature of the nucleotide base does not appear to influence NPP binding, despite the presence of a nucleoside being important (pyrophosphate induces a much smaller emission response in [**Eu.2**]^+^ compared with the NDPs, Fig. 2g). Similarly, the emission response of the Eu(iii) complexes towards the adenosine and the deoxyadenosine phosphate series are essentially the same, indicating minimal interaction with the ribose hydroxyl groups.

For each Eu(iii) complex, the magnitude of the intensity change upon adding NTPs or NDPs varies between the Eu(iii) emission bands, with particularly large changes observed for the hypersensitive Δ*J* = 2 (605–630 nm) band. [**Eu.4**]^+^ shows similar changes in emission spectral form to [**Eu.1**]^+^, but with much smaller intensity increases. Both complexes present similar new signals in both the Δ*J* = 1 and Δ*J* = 2 bands, following addition of ATP or ADP, with only intensity increases observed in the Δ*J* = 4 band (Fig. S14–S16†). However, addition of AMP to [**Eu.1**]^+^ gives rise to a unique Δ*J* = 2 band, featuring two lines of equal intensity, which is not observed with [**Eu.4**]^+^, indicating that [**Eu.1**]^+^ and [**Eu.4**]^+^ have similar binding modes to ATP and ADP, but distinct binding modes to AMP.

Complex [**Eu.3**]^+^, bearing *cis*-related quinoline groups, displays much smaller emission intensity increases compared with the other complexes (Fig. 2d and S8†). [**Eu.2**]^+^ shows large enhancements in emission intensity but does not show distinctive changes in spectral form on addition of ATP, ADP, or AMP, suggesting that [**Eu.2**]^+^ undergoes only minor conformational changes upon anion binding.

The fine structure of the observed transitions in the Eu(iii) emission spectra is a consequence of crystal field effects; the ligand environment causes splitting of both the ground and excited states into Stark sub-levels. The nature of the Stark splitting in the Δ*J* = 1 emission band (582–605 nm) can provide insight into the local symmetry around the Eu(iii) ion.^45^ Analysis of the change in fine structure of the Δ*J* = 1 band of [**Eu.1**]^+^ and [**Eu.4**]^+^, with added ATP or ADP, revealed a change in magnitude and sign of the second order crystal field parameter, *B*_0_^2^ (Fig. S14†). This indicates a significant change to the crystal field around the Eu(iii) centre. This possibly suggests a change between a twisted square antiprismatic structure (TSAP) in the absence of added anion, to a square antiprismatic (SAP) structure when ATP or ADP is bound.^46^ However, interpretation of the current spectroscopic results is by no means unambiguous.^47,48^ For complexes [**Eu.2**]^+^ and [**Eu.3**]^+^, no significant change in the splitting pattern of the Δ*J* = 1 band was observed in the presence of ATP or ADP, indicating no change in sign of the crystal field parameter, and smaller changes in coordination geometry upon anion binding.

An important factor to consider when designing synthetic receptors for NPP anions, is the competitive interaction of Mg^2+^ and Ca^2+^ ions with NPP anions (particularly NTP anions) *in vivo*.^49^ Consequently, the impact of Mg^2+^/Ca^2+^ ions competing with the synthetic receptors for NPP binding should be assessed. The addition of Mg^2+^ ions also increases the ionic strength of the solution, decreasing the strength of electrostatic interactions between host and guest. Addition of 5 mM MgCl_2_ to the receptor–anion solutions leads to an overall quenching of luminescence, with no effect on the emission intensity of the Eu(iii) complexes alone (Fig. 2b and S6–S13†). A decrease in emission intensity is particularly prominent with the NTP anions (which are known to bind most strongly to Mg^2+^ ions), followed by NDPs, while NMPs and other phosphoanions show only small decreases, attributed to the increase in ionic strength rather than any specific anion–Mg^2+^ interactions. Notably, discrimination between ATP and ADP (and between GTP and GDP) by [**Eu.1**]^+^ is enhanced significantly in the presence of Mg^2+^ ions (Fig. 2b and S5†).

### Evaluation of phosphoanion binding affinities

Binding constants were determined for ATP, ADP and AMP with complexes [**Eu.1**]^+^, [**Eu.2**]^+^ and [**Eu.4**]^+^ in 10 mM HEPES buffer (pH 7.–0). Due to the minor changes in the emission spectrum of [**Tb.1**]^+^, even on addition of high anion concentrations (up to 100 mM, Fig. S25†), binding constants could not be determined. Incubation of [**Eu.3**]^+^ with ATP or ADP lead to a gradual decrease in Eu(iii)-centred emission and a concomitant increase in quinoline fluorescence over time, indicating decomplexation of [**Eu.3**]^+^ (Fig. S26†). It is hypothesised that the binding of ATP (or ADP) to [**Eu.3**]^+^ creates an unfavourable steric interaction with the two *cis*-related quinoline groups, causing displacement of the pendant arms from the Eu(iii) metal centre. As such, luminescence titrations could not be performed with [**Eu.3**]^+^, ruling out this complex as a kinetically stable phosphoanion receptor, and confirming the importance of the *trans* configuration of the quinoline groups for anion recognition.

Values of apparent binding constants for [**Eu.1**]^+^, [**Eu.2**]^+^ and [**Eu.4**]^+^ with ATP, ADP and AMP are given in Table 2,^36,38^ and full titration data and binding isotherms are given in Fig. S27–S29.† In general, each Eu(iii) complex shows similar binding affinities for ATP and ADP, whereas AMP binds with an order of magnitude lower affinity. Complex [**Eu.2**]^+^ exhibits the strongest binding to nucleoside phosphate anions, followed by [**Eu.1**]^+^ and [**Eu.4**]^+^. Complexes [**Eu.2**]^+^ and [**Eu.1**]^+^ differ only in the nature of two pendant donor groups: the charged carboxylate donors in [**Eu.1**]^+^ have been replaced with neutral carbonyl amide groups in [**Eu.2**]^+^. This increases the electropositive nature of the Eu(iii) metal centre in [**Eu.2**]^+^, which strengthens the electrostatic component of the Eu(iii)–phosphate bonds, increasing binding affinities by at least one order of magnitude. The presence of the peripheral negatively charged carboxylates in [**Eu.2**]^+^ might be expected to decrease affinity for anions; however, this is clearly outweighed by the increase in local positive charge at the metal centre.

**Table 2:** Apparent binding constants (log *K*_a_) of Eu(iii) complexes [**Eu.1**]^+^, [**Eu.2**]^+^ and [**Eu.4**]^+^ with ATP, ADP and AMP in the absence and presence of MgCl_2_^a^

Complex	ATP		ADP		AMP	
	0 mM MgCl_2_	5 mM MgCl_2_	0 mM MgCl_2_	5 mM MgCl_2_	0 mM MgCl_2_	5 mM MgCl_2_
[**Eu.1**]^+^	4.4	2.82 ± 0.05	4.6	3.37 ± 0.02	3.4	3.40 ± 0.02
[**Eu.2**]^+^	5.8	n.d.	5.7	4.6	4.8	3.8
[**Eu.4**]^+^	3.65 ± 0.04	3.26 ± 0.20	3.34 ± 0.01	2.93 ± 0.05	2.77 ± 0.03	2.85 ± 0.03

a Conditions: 10 mM HEPES, pH 7.0, *λ*_exc_ = 330 nm, n.d. = not determined.

Replacing the quinoline amide groups in [**Eu.1**]^+^ with methoxy groups in [**Eu.4**]^+^ results in lower apparent binding affinities, by approximately one order of magnitude. This is ascribed to the amide groups in [**Eu.1**]^+^ acting as hydrogen bond donors to the coordinated anion, whereas [**Eu.4**]^+^ is unable to engage in hydrogen bonding. This suggests a cooperative effect of Eu(iii)–phosphate interactions, combined with hydrogen bonding in [**Eu.1**]^+^ and [**Eu.2**]^+^, leading to high affinity polyphosphate binding in aqueous solution.

In a competitive background of 5 mM MgCl_2_, there is generally an order of magnitude lower affinity for both ATP and ADP, whereas there is no effect on the binding affinity to AMP. This is consistent with the specific interaction between Mg^2+^ ions and ATP or ADP competing for binding with the Eu(iii) complex, compared with the weaker interaction between AMP and Mg^2+^. An exception to this is [**Eu.2**]^+^, where the ATP titration in the presence of 5 mM MgCl_2_ produced a two-step binding profile, which prevented the determination of a binding constant, due to the occurrence of multiple equilibria involved in the binding of [**Eu.2**]^+^ to both ATP and ATP–Mg, as indicated by previously reported mass spectral data.^38^

Binding of ATP, ADP and AMP to [**Eu.1**]^+^, [**Eu.2**]^+^ and [**Eu.4**]^+^ was investigated further by measuring the luminescence lifetimes in H_2_O and D_2_O, and calculating the number of coordinated water molecules, *q*, using the modified Horrocks equation (Table S10†).^40^ This revealed that *q* = 1 for each complex in the absence of added anions, but *q* = 0 in the presence of ATP or ADP, consistent with displacement of the coordinated water molecule from each complex upon binding ATP or ADP. In the presence of 5 mM AMP, a *q* value of 0.3–0.7 was found, indicating partial hydration, possibly reflecting the weaker monodentate binding of AMP, in accordance with previous examples of phosphate binding at lanthanide centres.^18,23,25^

### Structural analysis of anion binding

Having demonstrated the importance of several structural elements of [**Eu.1**]^+^ for nucleoside polyphosphate recognition, including the *trans* configuration of the quinoline groups, the presence of two hydrogen bonding amide groups, and the europium(iii) ion, we investigated the mode of binding of nucleotide polyphosphate anions to [**Eu.1**]^+^, *via* solution NMR spectroscopy, X-ray diffraction and DFT molecular modelling.

#### Single crystal X-ray diffraction

**Fig. 3:**
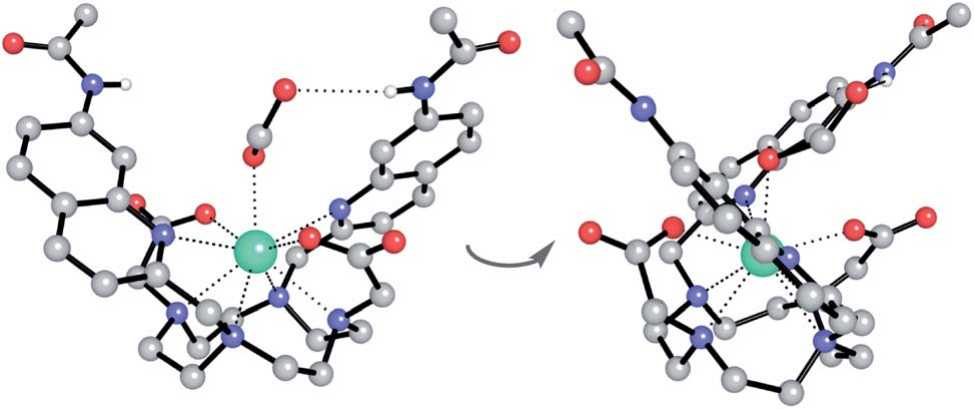
Two views of the X-ray crystal structure of the ternary adduct of [**Eu.1**]^+^ and formate, crystallised from acetonitrile/water (1 : 1). Key: Eu turquoise, C grey, N blue, O red, H white. Most H atoms and 7 water molecules of crystallisation are omitted for clarity.

Further evidence for the cooperative binding of anions to [**Eu.1**]^+^*via* metal–ligand and hydrogen bonding interactions came from X-ray analysis of [**Eu.1**]^+^ (Fig. 3 and S30–S32†). Despite multiple attempts to grow single crystals of ATP and ADP adducts of [**Eu.1**]^+^, we were unable to obtain crystals. However, colourless crystals of [**Eu.1**]^+^ bound to formate (present from the acidic method of RP-HPLC purification) were obtained by slow evaporation of a 1 : 1 mixture of acetonitrile/water. The Eu(iii) complex crystallised in the high symmetry orthorhombic space group *Fddd* and lies on a two-fold axis, which lies along the Eu(iii) to coordinated formate oxygen vector. The Eu(iii) ion is 9-coordinate, adopting a square antiprismatic geometry with the octadentate ligand, involving four nitrogen atoms from the macrocyclic ring, two oxygens from the carboxylate groups and two nitrogen atoms from the quinoline groups, which are oriented on the same face of the macrocycle but in opposite directions. A single formate anion occupies the axial position and is bound to the Eu(iii) ion in a monodentate manner. Notably, there are intermolecular N–H⋯O contacts between the quinoline amide N–H and the second oxygen atom of formate, confirming the ability of the quinoline amide groups to engage in hydrogen bonding to a coordinated anion.

#### Solution NMR studies

Further insight into the binding geometry of ATP and ADP to [**Eu.1**]^+^ in solution was gained by ^1^H and ^31^P NMR spectroscopy. The ^1^H NMR spectrum of [**Eu.1**]^+^ in the presence of either ATP or ADP (1 : 1 D_2_O/CD_3_OD, pD 7.4, Fig. S33†) revealed significant line broadening. This could be due to several factors, including an increase in conformational freedom in the host–guest complex, rapid exchange between the bound and unbound species occurring faster than the NMR timescale, or the formation of several host–guest complexes.

**Fig. 4:**
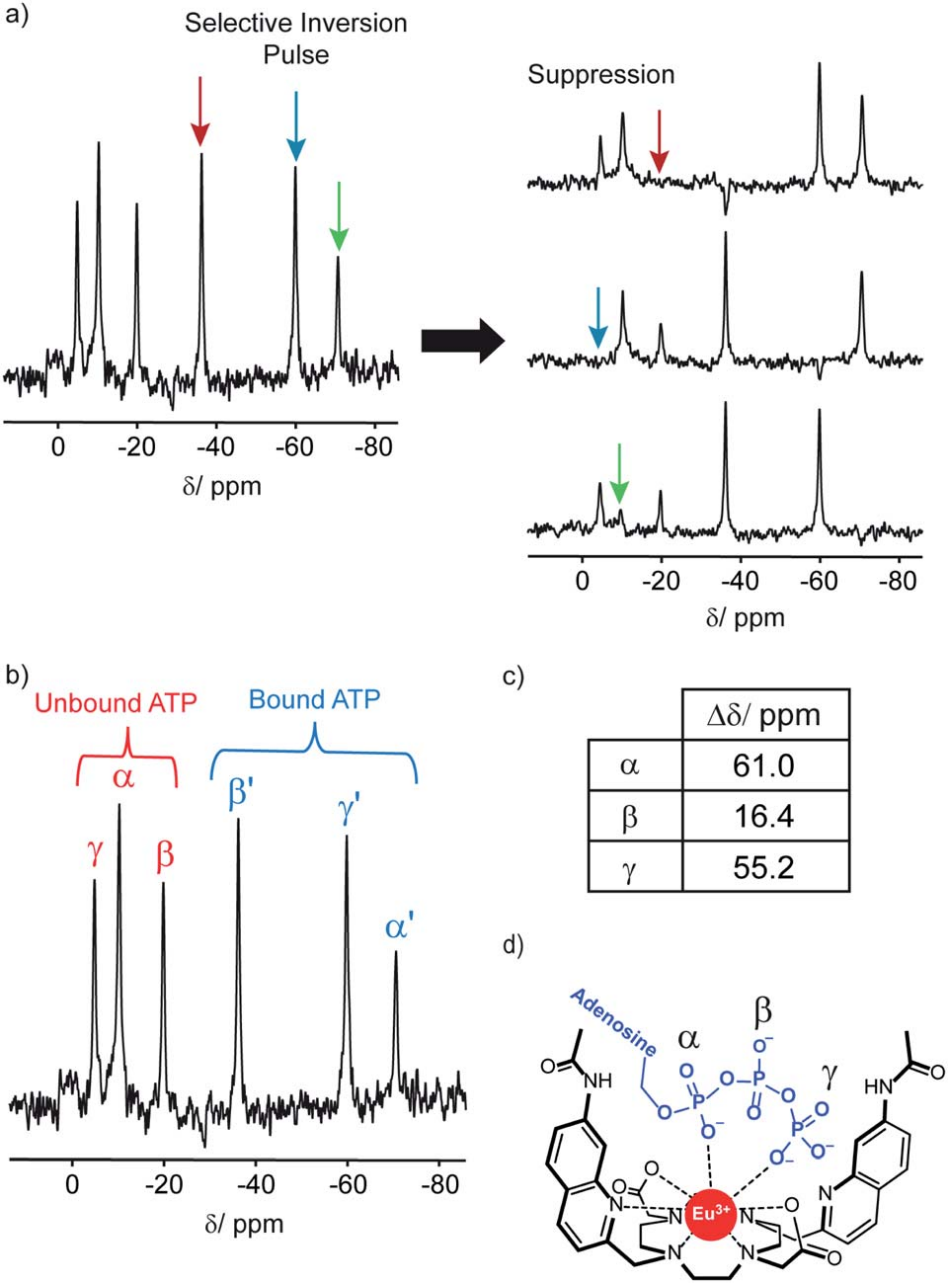
^31^P NMR (202.21 MHz) of ATP (6.57 mM) with [**Eu.1**]^+^ (6.57 mM) in 1 : 1 D_2_O : MeOD, pD 7.0, indicating bidentate binding of ATP to the europium(iii) ion. (a) ^31^P NMR selective inversion experiment, wherein selective inversion of one of the bound ATP ^31^P signals leads to suppression of the corresponding unbound ^31^P signal, due to rapid exchange on the NMR timescale. (b) ^31^P NMR of ATP + [**Eu.1**]^+^, showing ^31^P signals for both bound and unbound ATP. (c) Change in chemical shift for the α, β and γ-phosphates between the bound and unbound ^31^P NMR signals. (d) Proposed bidentate binding mode of ATP to [**Eu.1**]^+^, *via* the α and γ-phosphate groups.

The ^31^P NMR spectral data was more informative; addition of ATP to [**Eu.1**]^+^ resulted in six distinct resonances the ^31^P NMR spectrum, indicating the presence of both unbound ATP, and ATP bound in a single host–guest complex (Fig. 4). Similarly, four distinct signals were observed for [**Eu.1**]^+^ in the presence of ADP (Fig. S34†). However, there are significant differences between the spectra: with ATP the three bound ATP signals are of similar line width to the unbound ATP, but with ADP, the bound peaks are significantly broader, indicating different binding modes for the two anions and the strong likelihood of exchange between more than one binding mode for ADP.‡‡The notion of exchange between multiple bound states of ADP is supported further by ^1^H and ^31^P NMR spectra of a 1 : 1 mixture of ADP : [**Eu.1**]^+^, recorded at variable temperatures (Fig. S36B†). Upon decreasing the temperature from 25 °C to −20 °C, the ^31^P spectra shows little change in the unbound ADP signals; however, one of the bound ADP signals shifts by 20 ppm. This is consistent with exchange between multiple bound states of ADP, with the populations of the different states changing with temperature. Such exchange explains why the linewidths for bound ADP are considerably larger than those observed for bound ATP.

In order to assign the ^31^P NMR signals for the [**Eu.1**]^+^–ATP complex to the α, β and γ-phosphates of ATP, a selective inversion experiment was undertaken. A selective inversion pulse was applied at each of the frequencies of the bound ATP signals, followed by a mixing time of 5 milliseconds, and the decrease in the intensity of the corresponding unbound ATP signal was recorded (Fig. 4A). This enabled any bound/unbound nuclei in fast exchange to be identified. The three bound ATP signals at −71, −37 and −60 ppm were assigned to the α, β and γ-phosphorus atoms, respectively (Fig. 4B). These assignments were verified by ^31^P EXSY experiments, where cross-peaks for the β- and γ-phosphates were observed (Fig. S35†).

The change in chemical shift (Δ*δ*) for the α, β and γ-phosphorus atoms of ATP upon binding to [**Eu.1**]^+^ were measured to be 61 ppm, 16 ppm, and 55 ppm, respectively (Fig. 4C). Hence, the α and γ-phosphates of the bound ATP are shifted substantially compared with the β-phosphate. Assuming that the observed shifts are predominantly dipolar (pseudocontact), they can be described to a first approximation, by eqn (1) and (2) below,^50^1
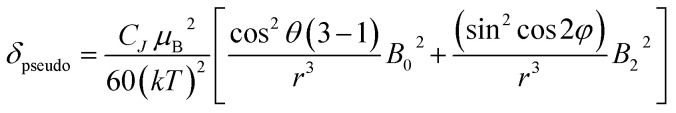

2 *C*_*J*_ = *g*_*J*_^2^〈*J*‖*α*‖*J*〉*J*(*J* + 1)(2*J* − 1)(2*J* + 3)

where *θ*, *φ*, and *r* define the polar coordinates and internuclear distance to the lanthanide(iii) ion, *C*_*J*_ is the Bleaney constant, *μ*_B_ is the Bohr magneton, *B*_0_^2^ and *B*_2_^2^ are second order crystal field splitting parameters, 〈*J*‖*α*‖*J*〉 is a numerical coefficient, *J* is the total angular momentum and *g* the electron g-factor (see ESI, Section 2.1 for further description†).

Thus, the chemical shifts depend both on the internuclear distance (1/*r*^3^) and on geometry factors. All of the shifts are in the same direction (to low frequency), which is as expected if the ATP binding pocket is approximately on the *C*_2_ symmetry axis of the Eu(iii) complex. Since there is a 1/*r*^3^ dependence of the pseudocontact shift on the internuclear distance from the paramagnetic centre, and assuming that the binding pocket lies on the symmetry axis, we propose that the distance factors will dominate and that the data therefore indicates a bidentate binding of ATP to [**Eu.1**]^+^, *via* the α and γ-phosphate groups (Fig. 4D). It is hypothesised that ATP binding causes displacement of one of the coordinated quinoline groups from the Eu(iii) ion, to accommodate the large polyphosphate anion and satisfy the preferred Eu(iii) coordination number of 9. This is consistent with a mechanism of binding proposed previously for adjacent phosphotyrosine residues in peptides to [**Eu.1**]^+^.^51^ The structure of the binding pockets of [**Eu.1**]^+^ and [**Eu.4**]^+^ are very similar, hence a similar binding mode is expected between ATP and [**Eu.4**]^+^. This is consistent with the almost identical changes in emission spectral form observed upon adding ATP to [**Eu.1**]^+^ and [**Eu.4**]^+^ (Fig. 1 and S14†).

A selective inversion experiment was also performed with ADP (Fig. S36†); however, inversion of each of the [**Eu.1**]^+^ bound ^31^P signals did not lead to any significant decrease in either of the unbound ADP signals, possibly due to the binding on/off rates between [**Eu.1**]^+^ and ADP being slower than the relevant NMR timescale (dominated by relaxation of the complexed species).

### DFT optimised structures of host-anion binding

#### [**Eu.1**]^+^ bound to nucleoside phosphate anions

Using the crystallographic data for [**Eu.1**]^+^, we modelled this complex bound to a single water molecule and to formate, which was present in the crystal structure (Fig. S37†). Then, we evaluated the feasibility of [**Eu.1**]^+^ to bind AMP, ADP and ATP, using a model system where the adenosine base was replaced with a methyl group. The mono-coordinated mode was initially explored for each of the phosphoanions (Fig S38a–c†). In all cases, the phosphoanion binds to the Eu(iii) complex though interactions with the metal centre and two hydrogen-bonding interactions. A monodentate binding mode does not, however, explain the differences in selectivity and emission spectral form observed between AMP and ADP/ATP (Fig. S27†). Further, it is not consistent with the NMR spectroscopic data for the ATP-[**Eu.1**]^+^ adduct, which suggests both the α and γ-phosphates interact directly with the Eu(iii) metal.

**Fig. 5:**
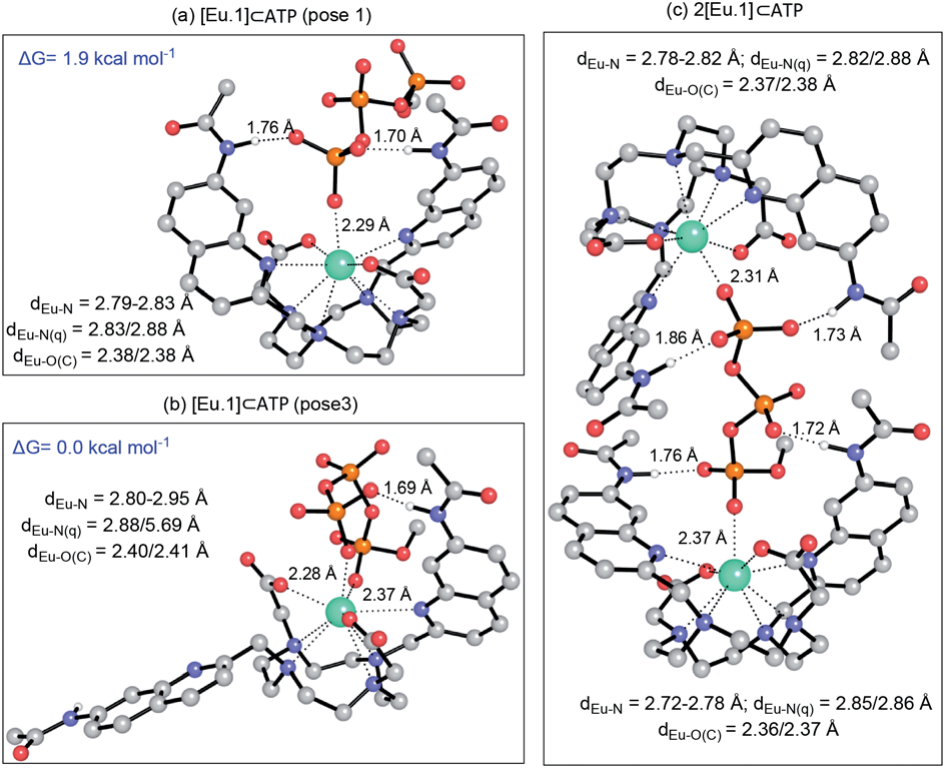
(a) Optimised geometries of the [**Eu.1**]⊂ATP complex with ATP bound in a (a) monodentate manner; (b) bi-coordinated with one quinoline group moved away from the coordination shell, and (c) as a 2 : 1 complex. All geometries were optimised at the PCM(H_2_O)-TPSSh/6-31G* level of theory. Hydrogens have been removed for clarity, except those that form H-bonds with the anion, for which distances are reported. Distances (Å) between the metal centre and its first coordination shell are shown for each mode. Relative energies for the [**Eu.1**]⊂ATP complex were calculated at the PCM(H_2_O)-M06-2X/6-311+G**//PCM(H_2_O)-TPSSh/6-31G* level of theory.

We hypothesised that the higher affinity of [**Eu.1**]^+^ for ATP arises from a bidentate interaction with the metal, *via* the α and γ-phosphate groups. Initial modelling of this binding mode led to significant changes in the coordination geometry, wherein both quinoline groups are displaced from the Eu(iii) metal centre (Fig. S38d†). It also caused weakening of the interaction between the metal centre and the four nitrogen atoms of the macrocyclic ring, suggesting dissociation of the Eu(iii) metal ion from the ligand. However, we have shown that when [**Eu.1**]^+^ is incubated with 10 mM ATP (or ADP) for 2 hours, the emission intensity and spectral form is almost identical to the immediate emission enhancement (Fig. S26†), confirming the formation of a stable host–guest complex. A second structure (Fig. 5b), where one of the quinoline groups is rotated away from the Eu(iii) ion, led to a more stable host–guest complex, involving bidentate binding of ATP, interaction of one quinoline nitrogen with the metal centre, and shorter interactions between the Eu(iii) centre and the four ring nitrogen atoms.

The possibility of a 2 : 1 [**Eu.1**]^+^:ATP complex was also considered, where one ATP molecule bridges two Eu(iii) metal centres, *via* the α and γ-phosphate groups (Fig. 5c). This structure shows four hydrogen bonding interactions between the amide N–H groups and the α, β and γ-phosphates of ATP. However, fitting of the data obtained from the titration of [**Eu.1**]^+^ with ATP to a 2 : 1 binding model, showed no substantial improvement in fitting compared with the 1 : 1 binding model (difference in the covariance of fit = 1.7).§§A 1 : 1 and 2 : 1 [**Eu.1**]^+^ : ATP binding model was used to fit the data obtained from the titration of [**Eu.1**]^+^ with ATP, using Bindfit [http://supramolecular.org]. The covariance of fit (cov_fit_) allowed comparison of the quality of the curve fitting between the 1 : 1 and 2 : 1 binding models (see ESI†). Due to the higher number of parameters from the 2 : 1 binding model, an improvement in the cov_fit_ by a factor greater than 3 would indicate that the 2 : 1 binding model was preferential.^58^ Compared to the 1 : 1 binding model, [**Eu.1**]^+^ showed no substantial improvement in fitting for the 2 : 1 binding model, with *F* cov_fit_ = 1.7. Evidence in support of a 1 : 1 binding mode between [**Eu.1**]^+^ and ATP (and ADP) was provided by high resolution mass spectrometric data, which revealed signals for the singly charged species [**Eu.1** + ATP + 2H]^−^ and [**Eu.1** + ADP + H]^−^, respectively (Fig. S39†). No species corresponding to a 2 : 1 complex was observed in electrospray mass spectrometry.

### Nucleoside phosphate discrimination in an array approach

The ability of Eu(iii) complexes [**Eu.1**]^+^, [**Eu.2**]^+^ and [**Eu.4**]^+^ to discriminate between NTPs, NDPs, and NMPs to varying degrees, combined with the distinctive changes in emission intensity and spectral form, suggested that these compounds could be excellent candidates for differential sensing in arrays. An array approach functions in a similar manner to a mammalian nose or tongue,^52^ where relatively low affinity binding of an analyte to a range of receptors leads to a fingerprint response pattern unique to that analyte. Arrays using synthetic receptors have been reported for the successful discrimination of anions,^53^ cations,^54^ small molecules, and large biomolecules.^55^

We considered that our Ln(iii)-based receptors would be particularly well suited to array sensing, as their luminescence response is information-rich, comprising multiple well-defined emission bands, each capable of offering a differential response to a specific anionic guest. Moreover, variations in the luminescence lifetimes of the complexes upon anion binding could be exploited to gain additional selectivity through time-resolved measurements. Finally, the differential effect of adding Mg^2+^ ions to the host–guest complexes should add an extra dimension to the response pattern, without the need for time-consuming synthesis of additional receptors.

To this end, we developed a high-throughput plate-based array, to determine the extent to which our lanthanide complexes (both individually and in combination) could discriminate between phosphate anions, using multivariate statistical techniques. We incubated the four stable complexes, [**Eu.1**]^+^, [**Eu.2**]^+^, [**Eu.4**]^+^ and [**Tb.1**]^+^, with and without 5 mM MgCl_2_ with a wide variety of phosphoanions, and calculated the percentage change in the luminescence intensity of the Δ*J* = 1, Δ*J* = 2, Δ*J* = 4, and a time-resolved Δ*J* = 2 intensity of the three Eu(iii) complexes (Fig. S40–S42†). Similarly, the percentage intensity changes for the Δ*J* = 1, Δ*J* = 2, Δ*J* = 3, Δ*J* = 4 and a time-resolved Δ*J* = 2 band of [**Tb.1**]^+^ complex were calculated (Fig. S43†). Overall, this allowed a fingerprint luminescence response for each anion to be attained. Principal component analysis (PCA) was carried out on this data and subsets thereof (Table S1†).^56^ This uses matrix techniques to calculate new orthogonal linear combinations (principle components) of the change in emission data, to give the maximum data variance in the minimum number of principle components, in this case 2 principle components have been used.^57^ This allows the data to be plotted as a 2-dimensional scatter plot, giving clustering of the data for different anions, allowing for their discrimination.

**Fig. 6:**
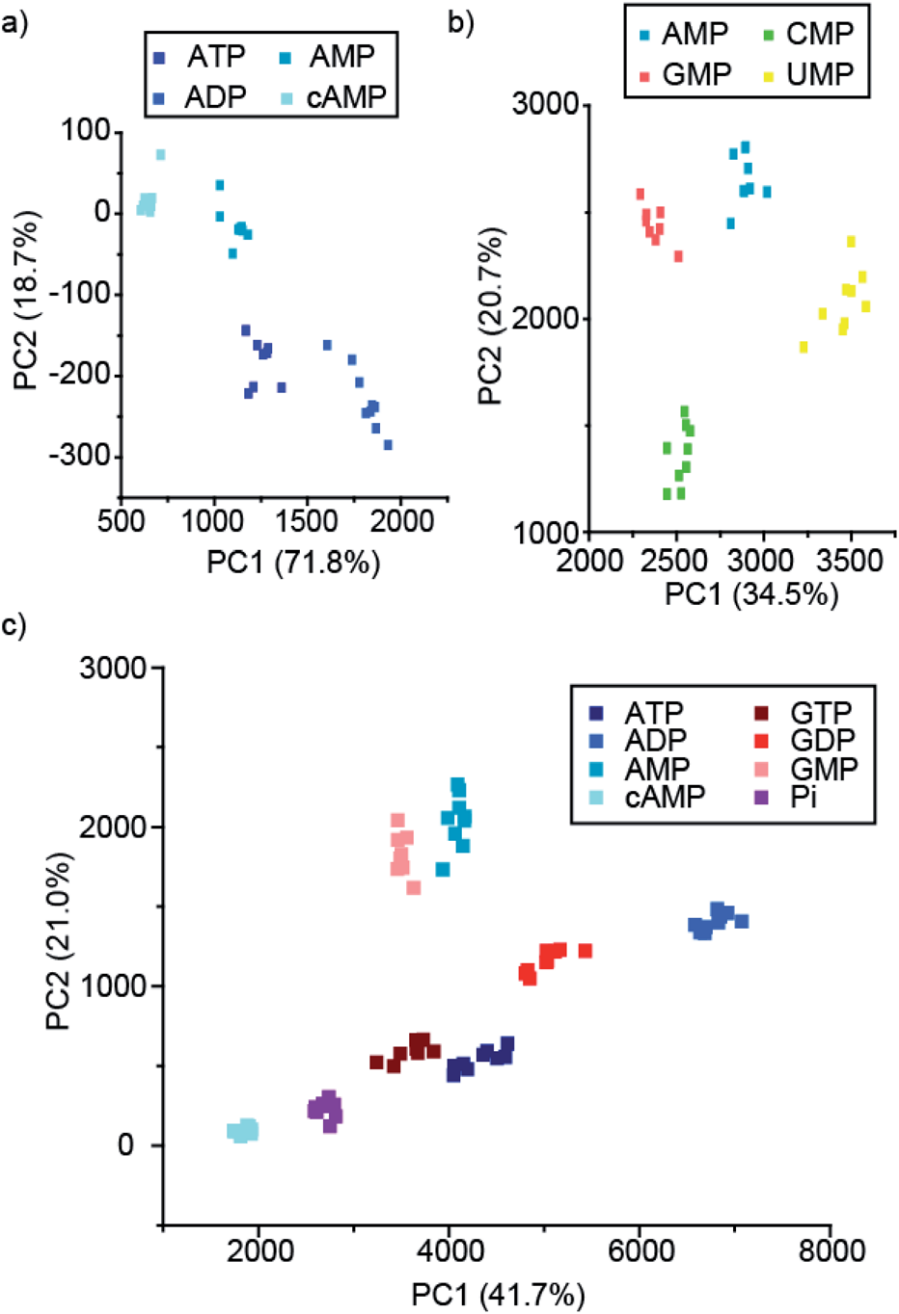
PCA score plots of % change in emission intensity of (a) just [**Eu.1**]^+^ (8 μM) with the adenosine series of anions (1 mM), (b) [**Eu.1**]^+^ (8 μM), [**Eu.2**]^+^ (13 μM), [**Eu.4**]^+^ (10 μM) and [**Tb.1**]^+^ (15 μM) with NMP anions (1 mM), and (c) the adenosine and guanosine series of NPP anions (1 mM), in 10 mM HEPES, pH 7.0.

Pleasingly, the principal component analysis for the luminescence bands of [**Eu.1**]^+^ and [**Eu.2**]^+^ individually (with and without MgCl_2_), show separate clusters for ATP, ADP, AMP and cAMP (Fig. 6a and S44†). This demonstrates the ability to discriminate these phosphoanions using a single lanthanide complex, by exploiting the information-rich emission bands.

Combining the luminescence data from the four anion receptors gives much tighter clusters on analysis of the same four anions (ATP, ADP, AMP and cAMP, Fig. S45†). Adding data for the NTPs, NDPs, and NMPs of the guanosine, cytidine, and uridine series (Fig. S45b†), shows evident clustering of each of the anions, with separate clusters for each of the NMPs, cAMP, and ADP. The other NTPs and NDPs display some overlapping clusters, although overall the NTPs and NDPs are separate.

Focusing on just the monophosphate anions with our four complexes there are clear and separate clusters for the four NMP anions, showing excellent discrimination (Fig. 6b). Similarly, if we reduce our analysis to just the purines (adenosine and guanosine) there is clear separation of the clusters for the anions ATP, ADP, AMP, cAMP, GTP, GDP, GMP and Pi (Fig. 6c), revealing that despite little base discrimination individually, in combination very good purine base discrimination can be achieved.

## Conclusion

We have shown that it is possible to modulate the phosphoanion binding and sensing properties of a series of Eu(iii)-based receptors in aqueous solution, through systematic modifications in the ligand structure. Specifically, the relative positions of the quinoline binding domains on the macrocyclic ligand were found to significantly impact on host–anion complex stability, with the *trans*-related quinoline groups providing sufficient flexibility to accommodate polyphosphate (*e.g.* ATP) binding and form stable ternary adducts, whereas the *cis*-orientation reduces stability of the host–anion complex, leading to dissociation of Eu(iii) over time. Anion affinity can be increased by an order of magnitude by incorporating hydrogen bond donor groups in the quinoline units, or by introducing neutral pendant Ln(iii) donor groups, thereby increasing the local positive charge at the Eu(iii) metal centre. We have shown that ATP binds to the parent host complex, [**Eu.1**]^+^, in a bidentate manner, *via* the α- and γ-phosphate groups, forming a stable host–guest complex in aqueous solution with fast binding kinetics on the NMR timescale.

Finally, we have demonstrated, for the first time, that lanthanide complexes are excellent candidates for differential anion sensing in a high-throughput array format. Using the emission bands of a single Eu(iii) complex, it was possible to discriminate between ATP, ADP, AMP, and cAMP using principal component analysis, whereas using a combination of four Ln(iii)-based receptors, which exhibit differential phosphoanion binding behaviour, it was possible to discriminate eight nucleoside phosphate anions (ATP, ADP, AMP, GTP, GDP, GMP, cAMP, Pi) in buffered aqueous solution. Our anion sensing array takes advantage of the information-rich emission spectra and variations in luminescence lifetimes of the Ln(iii) receptors, generating additional anion selectivity through time-resolved measurements.

In future designs of Ln(iii)-based anion receptors, it should be possible to integrate additional recognition motifs within the ligand structure that enable cooperative non-covalent interactions with the different structural components of a specific NPP anion. Research is being carried out in our laboratories in this regard, and will be reported in due course.

## Experimental

### Crystallography

#### Crystal data for [**Eu.1**]^+^·7H_2_O

C_37_H_61_EuN_8_O_15_·7(H_2_O), *M* = 1009.89, orthorhombic, *a* = 18.1194(3), *b* = 20.3557(3), *c* = 43.9834(7) Å, *U* = 16 222.5(4) Å^3^, *T* = 100(2) K, space group *Fddd*, *Z* = 16, 46 525 reflections measured, 4666 unique (*R*_int_ = 0.025), which were used in all calculations. The final w*R*(*F*^2^) was 0.115 (all data) and *R*_1_ was 0.046 (for 4373 reflections with *I* > 2*σ*(*I*)). Further details are given in the ESI.† CCDC 1975607 contains the supplementary crystallographic data for this paper.†

## Conflicts of interest

There are no conflicts to declare.

## Supplementary Material

SC-011-D0SC00343C-s001

SC-011-D0SC00343C-s002
