# Supplementary material for: Tuning the anion binding properties of lanthanide receptors to discriminate nucleoside phosphates in a sensing array
Source: Chem Sci. 2020 Mar 11;11(14):3619–28. doi: 10.1039/d0sc00343c (PMC8152522; doi:10.1039/d0sc00343c)
Supplement: SC-011-D0SC00343C-s001 [file SC-011-D0SC00343C-s001.pdf]

Electronic Supplementary Material (ESI) for Chemical Science.
This journal is © The Royal Society of Chemistry 2020

Supporting Information

Tuning the anion binding properties of lanthanide receptors to discriminate nucleoside phosphates in a sensing array

Sarah H. Hewitt,[a] Georgina Macey,[a] Romain Mailhot,[a] Mark R. J. Elsegood,[a] Fernanda Duarte,[b] Alan M. Kenwright[c] and Stephen J. Butler*[a]

[a]*Department of Chemistry, Loughborough University, Epinal Way, Loughborough, LE11 3TU, UK.*

[b]*Chemistry Research Laboratory, University of Oxford, 12 Mansfield Road, Oxford OX1 3TA, UK*

[c]*Department of Chemistry, Durham University, South Road, Durham, DH1 3LE, UK.*

1. Experimental Section

1.1 General considerations

^{1}H, ^{13}C, COSY and HMQC NMR spectra were recorded in the stated deuterated solvent on a JEOL ECS-400 spectrometer (^{1}H at 399.782 MHz, ^{13}C at 100.525 MHz), or a Bruker Advance Ultra-Shield 400 spectrometer (^{1}H at 400.134 MHz, ^{13}C at 100.624 MHz) at 293 K. Chemical shifts are expressed in ppm and are adjusted to the chemical shift of the residual NMR solvent resonances ($CDCl_3$: ^{1}H δ = 7.26 ppm, ^{13}C δ = 77.16 ppm, CD_3OD: ^{1}H δ = 3.31 ppm, ^{13}C δ = 49.00 ppm or D_2O: ^{1}H δ = 1.56 ppm). The coupling constants are expressed in Hz.

Liquid Chromatography Electrospray Mass spectra were recorded on a Shimadzu Prominence LC system with a Shimadzu SPDM20A Photodiode Array Detector, a Shimazu CTO-20A column oven, Shimadzu SIL-20A autosampler and a Shimadzu LCMS 20 mass spectrometer controlled using LabSolutions software. The system operates in positive or negative ion mode, with acetonitrile as the carrier solvent. The flow rate was maintained at 1.5 mL/min over a gradient of 5 to 95% acetonitrile (0.1% formic acid) in water (0.1% formic acid) for 8 minutes. High resolution mass spectra were recorded using a Thermofisher Q-Exactive orbitrap mass spectrometer.

Column chromatography was performed using flash silica gel 60 (particle size 40–63 microns) purchased from Apollo scientific. Thin layer chromatography (TLC) was performed on aluminium sheet silica gel plates with 0.2 mm thick silica gel 60 F_{254} using the stated mobile phase.

Preparative RP-HPLC was performed using a Waters 2489 UV/Visible detector performed at 254 nm, a Waters 1525 Binary HPLC pump controlled by the Waters Breeze 2 HPLC system software. Separation was achieved using a semi-preparative XBridge C18 (5 µm OBD 19 × 100 mm) column at a flow rate maintained at 17 mL min^{-1}. A solvent system composed of either water (0.1% formic acid) / methanol (0.1% formic acid) or water (50 mM NH_4HCO_3) / acetonitrile was used over the stated linear gradient (usually 0 to 100% organic solvent over 10 min). Analytical RP-HPLC was performed using a XBridge C18 5 µm 4.6 × 100 mm at a flow rate maintained at 2.0 mL min^{-1} using the same gradients and solvents.

1.2 Compound Synthesis and Characterisation

[**Eu.1**]$^{+}$, [**Eu.2**]$^{+}$ and *N*-(2-(hydroxymethyl)quinolin-7-yl)acetamide (**1**) were synthesised using previously reported methods.[1,2]

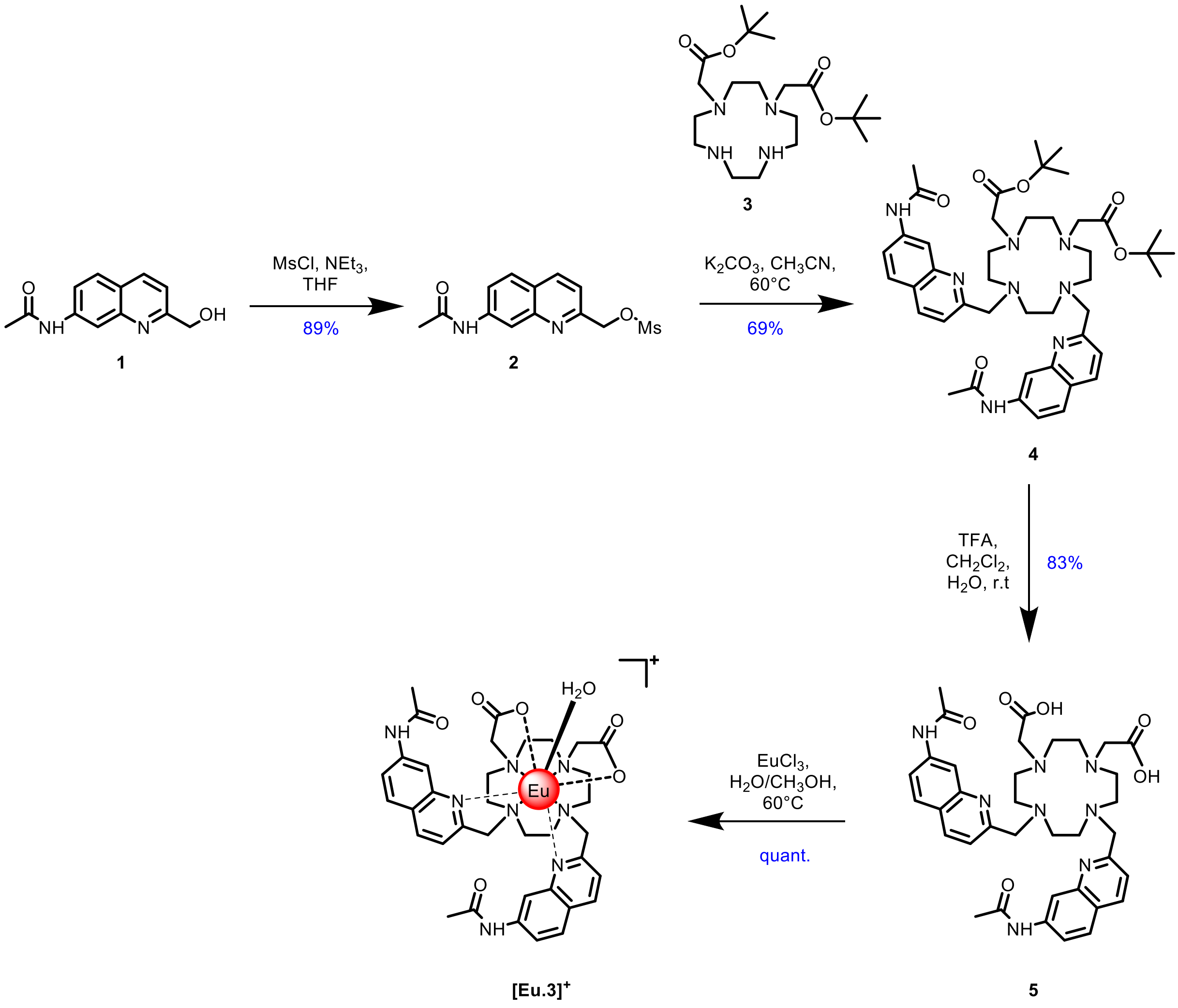

Scheme 1: Synthesis of complex **[Eu.3]**$^+$

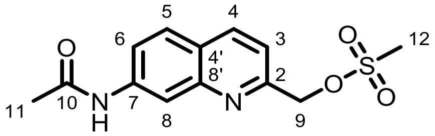

7-Acetamidoquinolin-2-yl)methyl methanesulfonate (2)

To a solution of *N*-(2-(hydroxymethyl)quinolin-7-yl)acetamide **1** (84 mg, 0.38 mmol) in anhydrous tetrahydrofuran (1 mL) was added di*iso*propylethylamine (100 µL, 0.58 mmol) and methanesulfonyl chloride (32 µL, 0.40 mmol). The reaction mixture was stirred for 2 hours at room temperature under a nitrogen atmosphere and then concentrated under reduced pressure. The resulting material was partitioned between water (10 mL) and dichloromethane (10 mL). The aqueous layer was extracted with dichloromethane (2 × 10 mL) and the organic layers combined, dried ($MgSO_4$) and the solvent removed under reduced pressure to give (7-acetamidoquinolin-2-yl)methyl methanesulfonate **2** (100

mg, 89%) as a yellow oil. The crude material was used immediately without any further purification. ^{1}H NMR (400 MHz, $CDCl_3$) δ 9.41 (s, 1H, NH), 8.29 (br. s, 1H, H^8), 8.01 (d, ^3J= 9.0, 1H, H^4), 7.81 (br. d, ^3J= 8.5 Hz, 1H, H^6), 7.60 (d, ^3J= 8.5 Hz, 1H, H^5), 7.34 (d, ^3J= 9.0 Hz, 1H, H^3), 5.36 (s, 2H, H^9), 3.05 (s, 3H, H^{12}), 2.19 (s, 3H, H^{11}); ^{13}C NMR (101 MHz, $CDCl_3$) δ 169.8 (C^{10}), 153.9 (C^2), 149.0 ($C^{8'}$), 140.5 (C^7), 137.1 (C^4), 128.12 (C^5), 124.4 ($C^{4'}$), 121.5 (C^6), 118.2 (C^3), 116.7 (C^8), 46.7 (C^9), 24.6 (C^{11}), 8.6 (C^{12}); LCMS: *m/z* (ESI^+) 295.1 $[M+H]^+$ ($C_{13}H_{15}N_2O_4S$ requires 295.1); R_f = 0.24 (silica, ethyl acetate/hexane, 50/50 v/v).

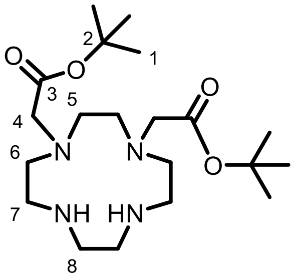

Di-*tert*-butyl-2,2'-(1,4,7,10-tetraazacyclododecane-1,4-diyl)diacetate (3)

1,4,7,10-Tetraazacyclododecane (2.00 g, 11.6 mmol) and triethylamine (16.2 mL, 116 mmol) were dissolved in anhydrous chloroform (200 mL) at room temperature. The reaction mixture was cooled to -5 °C and a solution of *tert*-butyl bromoacetate (3.43 mL, 23.2 mmol) in chloroform (25.0 mL) was added dropwise over a period of 2 hours. The reaction mixture was then stirred at room temperature overnight. The organic solvent was evaporated, and the resulting residue was dissolved in deionised water (100 mL). The pH was then adjusted to 3 by the addition of aq. of HCl. Undesired over-alkylated products were extracted with chloroform (4 x 50 mL). The pH was then adjusted to 6 by addition of aq. NaOH and the aqueous layer was washed again with chloroform (4 x 50 mL) to remove remaining over-alkylated product. Finally, the pH was adjusted to 7-8 and the desired product was extracted with chloroform (4 x 50 mL). The organic layers were combined, dried over $MgSO_4$ and concentrated under reduced pressure. The residue was purified by column chromatography (silica gel, CH_2Cl_2/CH_3OH 98:2 v/v to CH_2Cl_2/CH_3OH, 84:16 v/v, with an increment of 2%) to give compound **3** (2.46 g, 6.13 mmol, 53%) as a slightly orange amorphous solid. ^{1}H NMR (400 MHz, $CDCl_3$) δ (ppm): 3.34 (4H, s, H^4), 3.10-2.96 (8H, m, 4 x CH_2 cyclen), 2.96-2.80 (8H, m, 4 x CH_2 cyclen), 1.43 (18H, s, H^1). HRMS: (ESI+) *m/z* 401.3121 $[M+H]^+$ ($C_{20}H_{41}N_4O_4$ requires m/z = 401.3122). Spectral data were in accordance with that reported in the literature.[3]

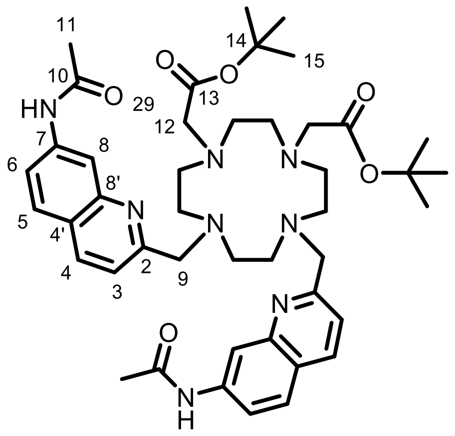

Di-tert-butyl 2,2'-(7,10-bis((7-acetamidoquinolin-2-yl)methyl)-1,4,7,10-tetraazacyclododecane-1,4 diyl)diacetate (4)

To a solution of the di-*tert*-butyl-2,2'-(1,4,7,10-tetraazacyclododecane-1,4-diyl)diacetate **3** (63 mg, 0.16 mmol) in anhydrous acetonitrile (8 mL) was added potassium carbonate (110 mg, 0.80 mmol) and (7-acetamidoquinolin-2-yl)methyl methanesulfonate **2** (103 mg, 0.35 mmol). The reaction mixture was stirred at 60 °C under an atmosphere of nitrogen for 18 hours. The resulting mixture was centrifuged (1500 rpm, 3 mins) and the filtrate separated from precipitated potassium salts and evaporated under reduced pressure. The crude material was purified by column chromatography (silica gel; neat dichloromethane to dichloromethane/methanol, 88/12 v/v) to give the protected macrocyclic ligand **4** (87 mg, 69%) as a white solid; ^{1}H NMR (400 MHz, $CDCl_3$) δ 8.15 (br. s, 2H, H^{4}), 7.98 (s, 2H, H^{8}) , 7.79 (d, ^{3}J = 8.0 Hz, 2H, H^{5}), 7.42 (d, ^{3}J = 8.0 Hz, 2H, H^{6}), 7.00 (br. s, 2H, H^{3}), 3.41 (s, 4H, H^{9}), 2.82 (br s, $4H^{12}$), 2.18 (s, 6H, H^{11}), 1.33 (s, 18H, H^{15}), signals for ring CH_2 are obscured, NH signals not observed; ^{13}C NMR (400 MHz, $CDCl_3$) δ 171.9 (C^{13}), 170.3 (C^{10}), 158.0 (C^{2}), 148.2 ($C^{8'}$), 140.6 (C^{7}), 136.5 (C^{4}), 127.7 (C^{5}), 123.8 ($C^{4'}$), 121.9 (C^{6}), 120.7 (C^{3}), 117.9 (C^{8}), 82.4 (C^{14}), 60.0 (cyclen CH_2), 56.5 (C^{9}), 50.6 (C^{12}), 49.6 (cyclen CH_2), 28.09 (C^{15}), 24.4 (C^{11}); *m/z* (ESI^{+}) 797.4709 $[M+H]^{+}$ ($C_{44}H_{61}N_8O_6$ requires 797.4693) R_f = 0.5 (silica, dichloromethane/methanol, 9/1 v/v).

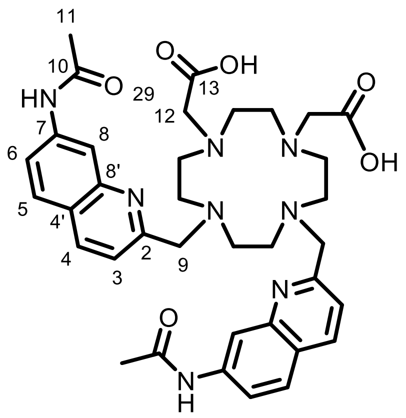

2,2'-(7,10-bis((7-acetamidoquinolin-2-yl)methyl)-1,4,7,10-tetraazacyclododecane-1,4-diyl)diacetic acid (5)

To a solution of the macrocyclic ligand **4** (87 mg, 0.11 mmol) in dichloromethane (5 mL) was added trifluoroacetic acid (5 mL). The reaction mixture was stirred at room temperature under an atmosphere of nitrogen for 18 hours. The solvent was removed under reduced pressure before purification by preparative RP-HPLC [gradient: 0 – 100% methanol in water (0.1% formic acid), over 10 min; t_R = 6.0 min] to give the deprotected ligand **5** (62 mg, 84%) as a yellow solid; ^{1}H-NMR (400 MHz, CD_3OD) δ 8.31 (s, 2H, H^8), 7.92 (d, ^3J = 8.0 Hz, 2H, H^4), 7.73 (d, ^3J = 8.5 Hz, 2H, H^5), 7.65(d, ^3J = 8.5 Hz, 2H, H^6), 7.42 (d, ^3J = 8.0 Hz, 2H, H^3), 4.19 (s, 4H, H^9), 3.64 (s, 4H, H^{12}), 2.24 (s, 6H, H^{11}), signals for ring CH_2 are obscured, NH signals not observed; ^{13}C-NMR (101 MHz, CD_3OD) δ 171.6 (C^{13}), 170.7 (C^{10}), 166.3 (C^2), 147.2 ($C^{8'}$), 140.2 (C^7), 137.1 (C^4), 127.91 (C^5), 124.25 ($C^{4'}$), 120.7 (C^6), 120.2 (C^3), 116.3 (C^8), 56.0 (C^9), 50.6 (C^{12}), 22.7 (C^{11}), macrocycle CH_2 signals were broad and difficult to determine. *m/z* (ESI^+) 685.3441 $[M+H]^+$ ($C_{36}H_{45}N_8O_6^+$ requires 685.3457).

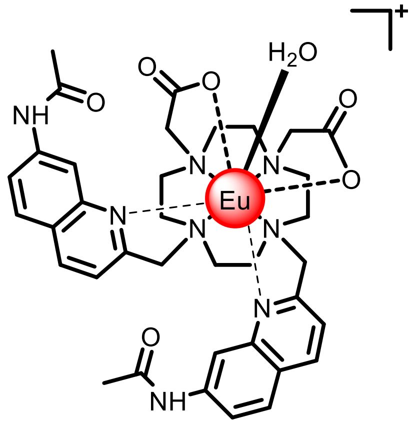

$[Eu.3]^+$

To a solution of deprotected ligand **5** (20 mg, 0.0291 mmol) in a mixture of water (4 mL) and methanol (4 mL) was added europium(III) chloride (11 mg, 0.029 mmol). The pH was adjusted to 8.0 by addition of NaOH (0.5 M) and the reaction mixture stirred at 60 °C for 18 hours. The methanol was removed

under reduced pressure and the water removed by lyophilisation, to give [**Eu.3**]$^+$ as a white solid (23 mg, quant.); ^{1}H NMR (500 MHz, MeOD) spectral range of 48 ppm (+30.6 to -17.6 ppm), 30.6, 27.7, 17.4, 13.3, 11.4, 8.7, 7.8, 7.6, 7.4, 3.4, 2.9, 2.6, 2.1, 2.0, -1.2, -3.3, -4.0, -5.4, -6.3, -6.6, -11.0, -12.5, -12.8, -13.4, -14.0, -17.6, N-H signals not observed; HRMS: *m/z* (ESI$^+$) 825.2422 [M+H]$^+$ ($C_{36}H_{42}EuN_8O_6^+$ requires 825.2434); λ_{max} = 328 nm; ε_{H2O} = 14000 M^{-1} cm^{-1}; Φ_{H2O} = 9.6%; $\tau_{(H2O)}$ = 0.65 ms, $\tau_{(D2O)}$ = 1.39 ms, hydration state, *q* = 0.7.

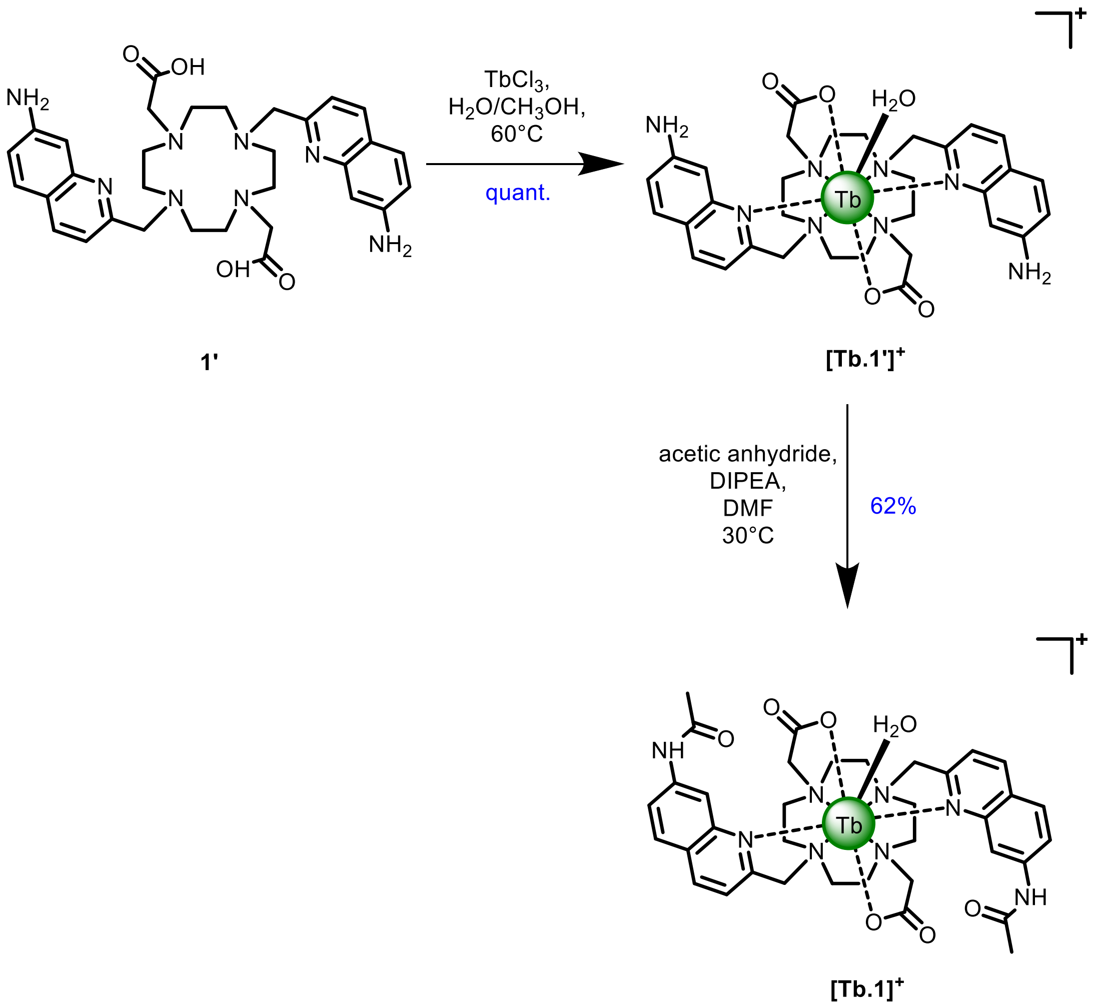

Scheme 2: Synthesis of complex [**Tb.1**]$^+$

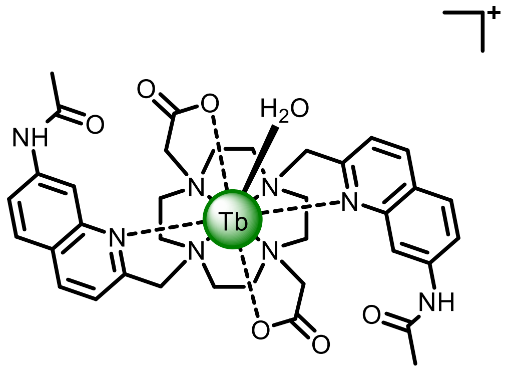

[Tb.1]$^+$

The deprotected ligand **1'** was synthesised according to previously reported methods.[1] To a solution of ligand **1'** (10 mg, 0.015 mmol) in a mixture of water (2 mL) and methanol (2 mL) was added terbium(III) chloride (6 mg, 0.016 mmol). The pH was adjusted to 8.0 by addition of NaOH (2 M) and the reaction mixture stirred at 60°C for 18 h. The methanol was removed under reduced pressure and the water by lyophilisation, to give the bis amine **[Tb.1']**$^+$ (12 mg, quant.) as a white solid. Next, **[Tb.1']**$^+$ (12 mg, 0.015 mmol) was dissolved in DMF (2 mL) and N,N-diisopropylethylamine (8 µL, 0.045 mmol) and acetic anhydride (4.5 µL, 0.045 mmol) were added, and the solution stirred at 30 °C for 24 hours. The mixture was cooled to room temperature and the crude material was purified by preparative RP-HPLC [gradient: 2 – 100% acetonitrile in 25 mM NH_4CO_3 over 15 min; t_R = 6.9 min], to give **[Tb.1]**$^+$ as a colourless solid (8 mg, 62%); HRMS *m/z* (ESI$^+$) 841.6736 [M+H]$^+$ ($C_{36}H_{42}TbN_8O_6^+$ requires 841.6952); λ_{max} = 332 nm; ε_{H2O} = 4,700 M^{-1} cm^{-1}.

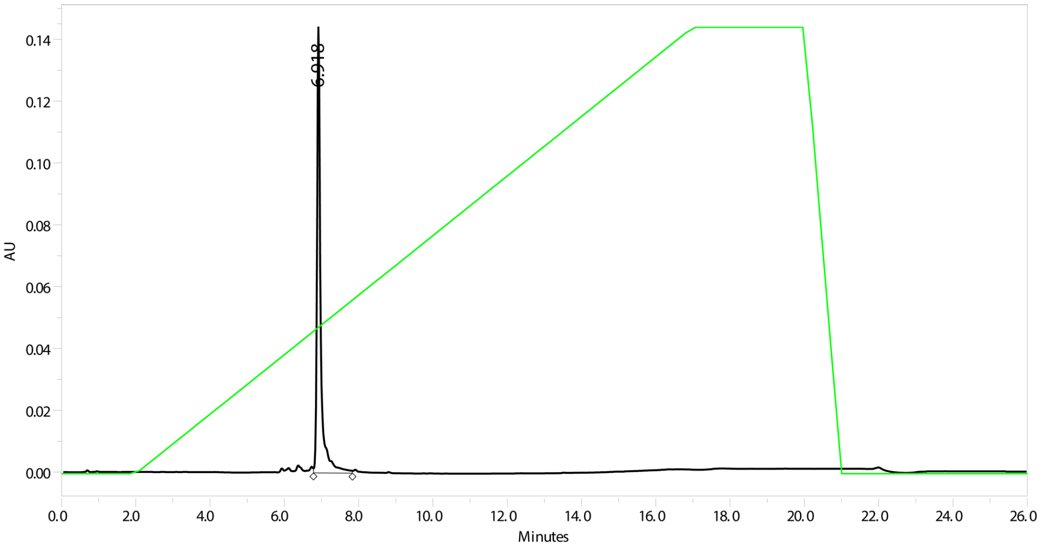

Analytical RP-HPLC trace of [**Tb.1**]$^+$; t_R = 6.91 min [gradient: 2 – 100% acetonitrile in 25 mM NH_4CO_3 over 15 min]

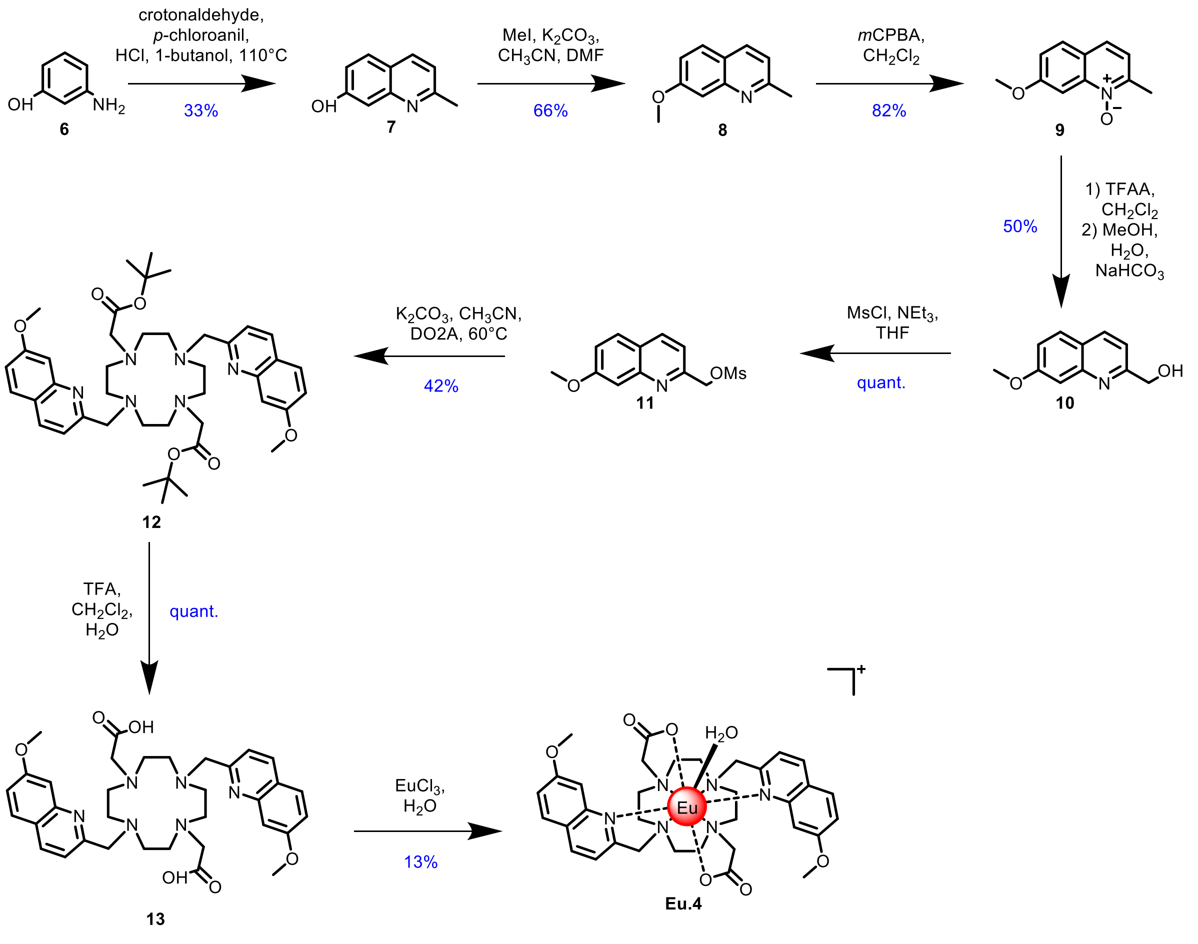

Scheme 3: Synthesis of complex **[Eu.4]**$^+$

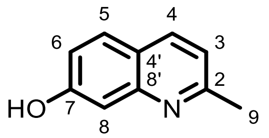

7-Hydroxy-2-methylquinaline (7)

3-Aminophenol **6** (3.85 g, 35.5 mmol) was dissolved in concentrated hydrochloric acid (10 mL). The reaction mixture was heated to 110 °C to help dissolution and stirred for 15 minutes. A solution of *p*-chloroanil (8.75 g, 35.5 mmol) in 1-butanol (12.5 mL) was added. Then over a period of 30 minutes a solution of crotonaldehyde (4 mL, 46.2 mmol) in 1-butanol (1 mL) was added. The reaction mixture was heated under reflux for 45 minutes. The solvent was removed under reduced pressure and the residue dissolved in water and washed with ether (3 × 25 mL). The aqueous layer was neutralised with 5 M sodium hydroxide and extracted with ethyl acetate (6 × 25 mL). The combined ethyl acetate phases were dried (magnesium sulfate), the solvent removed under reduced pressure and the crude material purified by column chromatography (silica gel, hexane/ethyl acetate neat to 3:2, v/v) to give

7-hydroxy-2-methylquinaline **7** (1.839 g, 33%) as an off-white solid; ^{1}H NMR (400 MHz, $CDCl_3$) δ 7.92 (d, ^{3}J = 8.2 Hz, 1H, H^4), 7.42 (d, ^{3}J = 8.8 Hz, 1H, H^5), 7.36 (d, ^{4}J = 2.3 Hz, 1H, H^8), 7.08 (d, ^{3}J = 8.2 Hz, 1H, H^3), 6.85 (dd, ^{3}J = 8.8 Hz, ^{4}J = 2.3 Hz, 1H, H^6), 2.71 (s, 1H, H^9); ^{13}C NMR (100 MHz, $CDCl_3$) δ 158.6 (C^2), 156.9 (C^7), 146.3 ($C^{8'}$), 136.4 (C^4), 127.7 (C^5), 119.9 ($C^{4'}$), 118.3 (C^3), 117.8 (C^6), 106.7 (C^8), 22.7 (C^9); *m/z* (ESI^+) 160.0754 $[M+H]^+$ ($C_{10}H_{10}NO^+$ requires 160.0757); R_f = 0.16 (hexane/ethyl acetate 3:2 v/v). Spectral data were in accordance with that reported in the literature.[4]

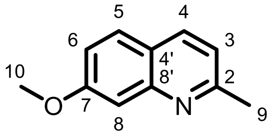

7-Methoxy-2-methylquinoline (8)

To 7-Hydroxy-2-methylquinaline **7** (500 mg, 3.14 mmol) and potassium carbonate (870 mg, 6.29 mmol) in anhydrous acetonitrile (22 mL) and anhydrous dimethyl formamide (2 mL) was added iodomethane (0.4 mL, 6.3 mmol) and the reaction mixture left to stir, under a nitrogen atmosphere, for 36 hours, at room temperature. Iodomethane (0.4 mL) in dimethylformamide (10 mL) was added and the reaction mixture stirred at 50°C for 18 hours. The solvent was removed under reduced pressure and the residue redissolved in ethyl acetate (3 x 20 mL) and washed with water (25 mL). The organic phases were combined, dried over magnesium sulfate and the solvent removed under reduced pressure. The crude material was purified by column chromatography (silica gel, hexane/ethyl acetate 1:1, v/v) to give 7-methoxy-2-methylquinoline **8** (355 mg, 66%) as a brown oil; ^{1}H NMR (400 MHz, $CDCl_3$) δ 7.93 (d, ^{3}J = 8.0 Hz, 1H, H^4), 7.62 (d, ^{3}J = 9.2 Hz, 1H, H^5), 7.34 (d, ^{4}J = 2.8 Hz, 1H, H^8), 7.12 (d, ^{3}J = 8.0 Hz, 1H, H^3), 7.11 (dd, ^{3}J = 9.2 Hz, ^{4}J = 2.8 Hz, 1H, H^6), 3.92 (s, 3H, H^{10}), 2.69 (s, 3H, H^9); ^{13}C NMR (100 MHz, $CDCl_3$) δ 160.8 (C^2), 159.3 ($C^{8'}$), 149.6 (C^7), 135.9 (C^4), 128.6 (C^5), 121.7 ($C^{4'}$), 119.9 (C^3), 118.8 (C^6), 106.9 (C^8), 55.6 (C^{10}), 25.4 (C^9); *m/z* (ESI^+) 174.0910 $[M+H]^+$ ($C_{11}H_{12}NO^+$ requires 174.0913); R_f = 0.34 (hexane/ethyl acetate 3:2 v/v). Spectral data were in accordance with that reported in the literature.[5]

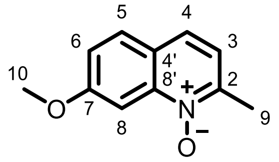

7-Methoxy-2-methylquinoline 1-oxide (9)

To 7-methoxy-2-methylquinoline **8** (305 mg, 1.76 mmol) in dichloromethane (8 mL) was added *meta*-chloroperoxybenzoic acid (603 mg, 3.49 mmol) and the reaction mixture stirred under a nitrogen atmosphere at room temperature for 4 hours. Reaction mixture was quenched with saturated aqueous sodium bicarbonate (10 mL) and the organic layer was extracted with saturated aqueous sodium bicarbonate (2 x 15 mL). The aqueous layer was backwashed with dichloromethane (2 x 20 mL). The organic phases were combined, dried (magnesium sulfate) and the solvent removed under reduced pressure to give 7-methoxy-2-methylquinoline 1-oxide **9** (271 mg, 82%) as a brown oil; ^{1}H NMR (400 MHz, $CDCl_3$) δ 8.12 (d, ^4J =2.4 Hz, 1H, H^8), 7.70 (d, ^3J = 9.2 Hz, 1H, H^5), 7.60 (d, ^3J = 8.8 Hz, 1H, H^3), 7.20 (dd, ^3J = 9.2 Hz, ^4J =2.4 Hz, 1H, H^6), 7.17 (d, ^3J = 8.8 Hz, 1H, H^4), 3.99 (s, 3H, H^{10}), 2.72 (s, 3H, H^9); ^{13}C NMR (100 MHz, $CDCl_3$) δ 162.1 (C^7), 146.7 (C^2), 142.9 ($C^{8'}$), 129.5 (C^3), 125.7 (C^5), 124.3 ($C^{4'}$), 120.8 (C^4), 120.4 (C^6), 98.3 (C^8), 56.0 (C^{10}), 19.0 (C^9); *m/z* (ESI^+) 189.0792 $[M+H]^+$ ($C_{11}H_{11}NO_2^+$ requires 189.0790); R_f = 0.012 (dichloromethane/methanol 49:1 v/v).

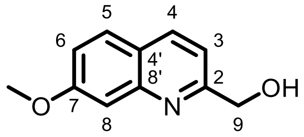

(7-methoxyquinolin-2-yl)methanol (10)

To 7-methoxy-2-methylquinoline 1-oxide **9** (50 mg, 0.26 mmol) in dichloromethane (2 mL) was added trifluoroacetic anhydride (0.15 mL, 1.06 mmol) and the reaction mixture stirred under a nitrogen atmosphere at room temperature for 2 hours. The solvent was removed under reduced pressure then the residue was redissolved in methanol (5 mL). Water (5 mL) and saturated aqueous sodium bicarbonate solution (5 mL) were added and the reaction mixture stirred for 16 hours. The solvent was removed under reduced pressure, and the residue was redissolved in dichloromethane (2 x 5 mL) and washed with saturated sodium bicarbonate solution (10 mL). The organic phases were combined, dried (magnesium sulfate) and the solvent removed under reduced pressure to give (7-methoxyquinolin-2-yl)methanol **10** (24 mg, 50%) as a yellow solid; ^{1}H NMR (400 MHz, $CDCl_3$) δ 8.04 (d, ^3J = 8.0 Hz, 1H, H^4), 7.68 (d, ^3J = 9.2 Hz, 1H, H^5), 7.38 (d, ^3J = 2.4 Hz, 1H, H^8), 7.17 (dd, ^3J = 9.2 Hz, ^4J = 2.4 Hz, 1H, H^6), 7.15 (d, ^3J = 8.0 Hz, 1H, H^3), 4.89 (s, 2H, H^9), 3.95 (s, 3H, H^{10}); ^{13}C NMR (100 MHz, $CDCl_3$) δ

161.0 (C^2), 159.3 ($C^{8'}$), 148.5 (C^7), 136.9 (C^4), 128.7 (C^5), 122.7 ($C^{4'}$), 119.3 (C^6), 116.1 (C^3), 106.9 (C^8), 64.2 (C^9), 55.6 (C^{10}); *m/z* (ESI$^+$) 189.0789 $[M+H]^+$ ($C_{11}H_{11}NO_2^+$ requires 189.0790); R_f = 0.28 (hexane/ethyl acetate 1:1 v/v).

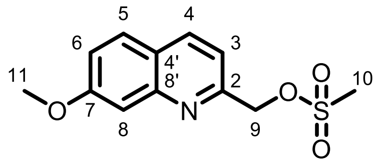

7-Methoxyquinolin-2-yl-methanesulfonate (11)

To methanesulfonyl chloride **10** (0.63 mL, 0.64 mmol) in anhydrous tetrahydrofuran (5 mL), at 0 °C was added (7-methoxyquinolin-2-yl)methanol (100 mg, 0.529 mmol) and triethylamine (0.15 mL, 1.1 mmol), and the reaction mixture left to stir for 30 minutes. The solvent was removed under reduced pressure, and the residue redissolved in dichloromethane (10 mL) and washed with brine (3 × 10 mL). The organic phases were combined, dried (magnesium sulfate) and the solvent removed under reduced pressure to give 7-methoxyquinolin-2-yl-methanesulfonate **11** (164 mg, quant.) as a yellow solid; ^{1}H NMR (400 MHz, $CDCl_3$) δ 8.22 (d, ^3J = 8.4 Hz, 1H, H^4), 7.70 (d, ^3J = 9.2 Hz, 1H, H^5), 7.55 (d, ^4J = 2.4 Hz, 1H, H^8), 7.50 (dd, ^3J = 8.4 Hz, 1H, H^3), 7.20 (d, ^3J = 9.2 Hz, ^4J = 2.4 Hz, 1H, H^6), 4.90 (s, 2H, H^9), 3.90 (s, 3H, H^{11}), 3.63 (s, 3H, H^{10}); R_f = 0.63 (dichloromethane/methanol 49:1 v/v).

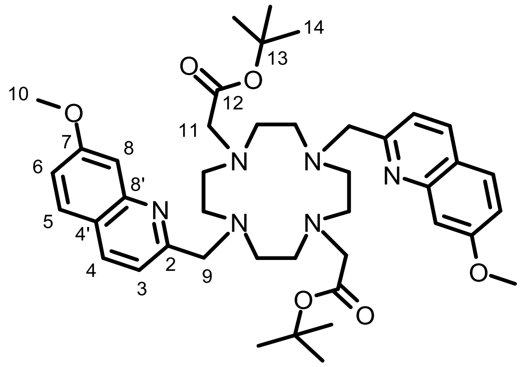

Di-tert-butyl-2,2'-(4,10-bis((7methoxyquinolin-2-yl)methyl)-1,4,7,10-tetraazacyclododecane-1,7-diyl)diacetate (12)

To 7-methoxyquinolin-2-yl-methanesulfonate **11** (172 mg, 0.643 mmol) in acetonitrile (10 mL), was added di-tert-butyl 2,2'-(1,4,7,10-tetraazacyclododecane-1,7-diyl)diacetate (109 mg, 0.27 mmol) and potassium carbonate (188 mg, 1.36 mmol) and the reaction mixture stirred at 60°C for 18 hours under a nitrogen atmosphere. The reaction mixture was centrifuged (3000 rpm for 5 minutes), the

precipitate removed and the solvent removed under reduced pressure. The resulting crude material was purified by column chromatography (silica gel, dichloromethane/methanol neat to 9:1, v/v) to give di-tert-butyl-2,2'-(4,10-bis((7-methoxyquinolin-2-yl)methyl)-1,4,7,10-tetraazacyclododecane-1,7-diyl)diacetate **12** (65 mg, 32%) as a yellow/brown oil; ^{1}H NMR (400 MHz, $CDCl_3$) δ 8.12 (d, ^{3}J = 8.4 Hz, 2H, H^{4}), 7.67 (d, ^{3}J = 9.2 Hz, 2H, H^{5}), 7.34 (d, ^{3}J = 2.4 Hz, 2H, H^{8}), 7.28 (d, ^{3}J = 8.4 Hz, 2H, H^{3}), 7.05 (dd, ^{3}J = 9.2 Hz, ^{4}J = 2.4 Hz 2H, H^{6}), 3.09 (s, 6H, H^{10}), 2.83 (s, 4H, H^{9}), 2.78 (s, 4H, H^{11}), 2.53 – 2.22 (16H, s, cyclen), 1.14 (18H, s, H^{14}); ^{13}C NMR (100 MHz, $CDCl_3$) δ 171.0 (C^{12}), 160.7 (C^{2}), 159.6 ($C^{8'}$), 149.4 (C^{7}), 137.3 (C^{4}), 128.8 (C^{5}), 122.6 ($C^{4'}$), 120.0 (C^{3}), 119.7 (C^{6}), 108.2 (C^{8}), 81.8 (C^{13}), 59.8 (C^{9}), 57.8 (C^{11}), 54.4 (C^{10}), 50.7 (cyclen CH_2), 39.5 (cyclen CH_2), 27.9 (C^{14}); *m/z* (ESI^+) 743.4476 $[M+H]^+$ ($C_{42}H_{59}N_6O_6^+$ requires 743.4491); R_f = 0.78 (dichloromethane/methanol 9:1 v/v).

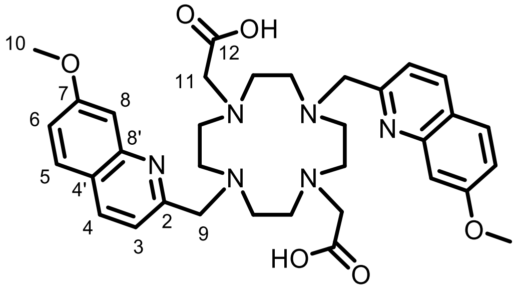

2,2'-(4,10-bis((7-methoxyquinolin-2-yl)methyl)-1,4,7,10-tetraazacyclododecane-1,7-diyl)diacetic acid (13)

Di-tert-butyl-2,2'-(4,10-bis((7methoxyquinolin-2-yl)methyl)-1,4,7,10-tetraazacyclododecane-1,7-diyl)diacetate **12** (13 mg, 0.017 mmol) was dissolved in trifluoroacetic acid (0.5 mL, 0.017 mmol) and water (1 drop), and the mixture stirred for 3 hours at room temperature. The solvent was removed under reduced pressure and the crude material was purified by preparative RP-HPLC [gradient: 0 – 100% methanol in water (0.1% formic acid), over 10 min] to give the deprotected ligand **13** (12 mg, quant.) as a white solid; ^{1}H NMR (400 MHz, $CDCl_3$) δ 8.34 (d, ^{3}J = 8.4 Hz, 2H, H^{4}), 7.84 (d, ^{3}J = 8.4 Hz, 2H, H^{3}), 7.79 (d, ^{3}J = 9.2 Hz, 2H, H^{5}), 7.22 (br. s, 2H, H^{8}), 7.17 (dd, ^{3}J = 9.2 Hz, ^{4}J= 2.4 Hz, 2H, H^{6}), 3.83 (s, 8H, H^{9} & H^{11}), 3.32 (s, 6H, H^{10}), 2.89 - 2.78 (br. m, 16H, cyclen); ^{13}C NMR (100 MHz, $CDCl_3$) δ 168.2 (C^{12}), 161.6 (C^{2}), 148.5 ($C^{8'}$), 141.5 (C^{7}), 140.4 (C^{4}), 129.9 (C^{5}), 123.3 ($C^{4'}$), 120.4 (C^{6}), 119.9 (C^{3}), 104.7 (C^{8}), 57.6 (C^{9}), 55.7 (C^{10}), 50.7 (C^{11}), 47.7 (cyclen); *m/z* (ESI^+) 631.3224 $[M+H]^+$ ($C_{34}H_{43}N_6O_6^+$ requires 631.3239); *m/z* (ESI^+) 653.3043 $[M+Na]^+$ ($C_{34}H_{42}N_6O_6Na^+$ requires 653.3058); R_f = 0.2 (dichloromethane/methanol 9:1 v/v).

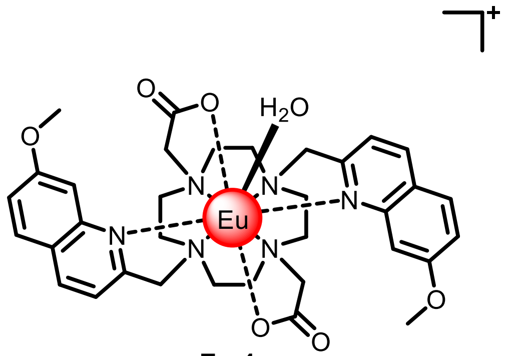

Eu.4

[Eu.4]$^+$

2,2’-(4,10-bis((7-methoxyquinolin-2-yl)methyl)-1,4,7,10-tetraazacyclododecane-1,7-diyl)diacetic acid (18 mg, 0.029 mmol) was dissolved in water (2 mL) and the pH was adjusted to 7.0, using 5 M sodium hydroxide and 5 M hydrochloric acid. Europium(III) chloride (7 mg, 0.019 mmol) was added and reaction mixture left to stir under nitrogen for 21 hours. The crude material was purified by preparative RP-HPLC [gradient: 0 – 100% methanol in water (0.1% formic acid), over 10 min; t_R = 8.48 min] to give **[Eu.4]$^+$** (1.8 mg, 13%) as a white solid; ^{1}H NMR (500 MHz, D_2O) spectral range of 56 ppm (+28.3 to -28.1 ppm), 28.3, 18.0, 15.2, 13.2, 10.8, 8.5, 7.8, 7.3, 3.9, 3.6, 3.1, 3.0, 2.6, 1.2, -2.2, -7.7, -17.4, -18.4, -27.0, -28.1, N-H signals not observed; *m/z* (ESI$^+$) 781.2197 [M]$^+$ ($C_{34}H_{40}N_6O_6^+$ requires 781.2216); R_f = 0.1 (dichloromethane/methanol 9:1 v/v); λ_{max} = 332 nm; ε_{H2O} = 10.1 mM cm^{-1}; Φ_{H2O} = 8.3% $\tau_{(H2O)}$ = 0.54 ms, $\tau_{(D2O)}$ = 1.28 ms, hydration state, q = 0.9.

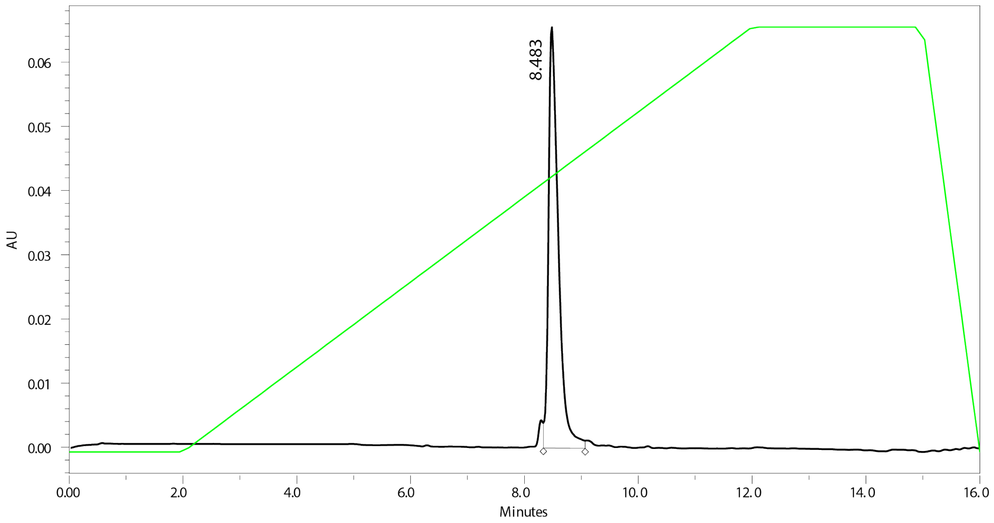

Analytical RP-HPLC trace of [**Eu.4**]$^+$; t_R = 8.48 min [gradient: 2 – 100% methanol in water (0.1% formic acid) over 10 min]

2. Spectroscopic analysis of lanthanide-based anion receptors

2.1. General considerations

All analyses were carried out in degassed 10 mM HEPES, pH 7.0 buffer. Concentrated Ln(III) complex stocks were made at ~2.5 mg mL^{-1} in methanol and the accurate concentration determined using the UV/Vis absorbance at their λ_{max} in 10 mM HEPES, pH 7.0. In all analysis, the Ln(III) complex concentration was kept constant at 8, 13, 6, 10, or 15 µM for [**Eu.1**]$^+$, [**Eu.2**]$^+$, [**Eu.3**]$^+$, [**Eu.4**]$^+$ and [**Tb.1**]$^+$ respectively, this corresponds to 0.1 absorbance. Stocks of anions were made up at 5 or 25 mM and adjusted to pH 7.0 by addition of minimal volumes of 2 M NaOH or 2 M HCl. Stocks of $MgCl_2$ were made up at 50 or 250 mM in 10 mM HEPEs, pH 7.0.

Optical spectroscopy

UV/Vis absorbance spectra were measured using a Shimadzu UV-1800 UV-spectrophotometer.

Emission spectra were recorded on an SPEX Fluoromax luminescence spectrometer using dM300 version 3.12 software. Emission spectra were obtained using a 40 µL Hellma® Analytics quartz cuvette (Art no. 111-10-K-40), unless stated otherwise. With excitation at 330 nm, and reading emission in the range 550 – 720 nm (for titrations) using an integration time of 0.5 seconds, increment of 0.5 or 1 nm and excitation and emission slits of 0.5 nm.

Overall luminescence quantum yields were measured in 10 mM HEPES buffer, by indirect excitation of the Eu(III) ion *via* the quinoline antennae using a previously reported 8-benzyloxyquinoline functionalized DO3A europium(III) complex, [**Eu.1**] (ϕ_{em}= 0.06 in water),[6,7] and rhodamine 101 in acidified ethanol as standards. Quantum yields have an estimated maximum uncertainty of ±20%.

Lifetime measurements were performed using a Camlin Photonics luminescence spectrometer with FluoroSENS version 3.4.7.2024 software. Lifetime measurements were typically obtained by indirect excitation of the Eu(III) ion *via* the quinoline antennae using a short pulse of light (at λ_{max}) followed by monitoring the integrated intensity of the light emitted at 615 nm during a fixed gate time, t_g, after a delay time, t_d. Measurements were made for a minimum of 20 delay times, covering 3 or more lifetimes. A gate time of 0.1 ms was used and the excitation and emission slits were set to 10 and 5 nm respectively. The obtained decay curves were plotted in Origin Labs 2019 (64-bit) version 9.6.0.172 and fitted to the equation:

$$I = A_0 + A_1e^{-kt}$$

Where I: intensity at time, t, following excitation; A_0: intensity when decay has ceased

A_1: pre-exponential factor; k: rate constant for the depopulation of the excited state.

Plate reader data was obtained on a BMG Labtech CLARIOstar microplate reader in black Fisherbrand™ 384-well plates, using a total volume of 40 µL per well.

Anion screening

Emission spectra of 50 µL of Ln(III) complex (6 – 13 µM) with the phosphoanions (1 mM), or none were taken in 10 mM HEPES, pH 7.0. 1 µL of 250 mM $MgCl_2$ was added, and the emission spectrum taken again. Graphs were plotted as the emission spectra themselves and as percentage differences of individual bands, or total emission with the anion compared to without the anion.

Anion binding titrations

Increasing volumes of solutions of an appropriate anion + Ln(III) complex stock in 10 mM HEPES, pH 7.0 were added to 50 µL of the Ln(III) complex (6 – 13 µM) and the emission spectrum taken after each addition. Appropriate ratios of either the ΔJ = 2/ΔJ = 1 (605 – 630/585 - 600) or Δ*J* = 2/Δ*J* = 0 (605 – 630/575 – 583) were taken, and plotted against anion concentration. This was fitted to a 1:1 binding isotherm (Eq. 1), using OriginLab, unless stated otherwise.

$$FB = \frac{\frac{1}{K_a} + [X] + [Eu] - \sqrt{(\frac{1}{K_a} + [X] + [Eu])^2 + 4[X][Eu]}}{2[Eu]}$$

FB: fraction bound, calculated by $(I-I_0)/(I_1-I_0)$, where I = intensity at [X], I_0 = initial intensity and I_1 = final intensity

[X]: total concentration of anion in solution

[Eu]: total concentration of Eu(III) complex

K_a: association constant

pH titration

A solution of [**Eu.3**]$^+$ (6 µM, 2mL) in 10 mM HEPES was adjusted to pH ~ 3 by addition of 2 M HCl and an emission spectrum taken. The pH was slowly adjusted (by ~0.25 pH units) by addition of 20, 200 or 2000 mM NaOH solution, and an emission spectrum taken at each pH. The total intensity of the Δ*J* = 2 band was plotted against the pH, and fit to a sigmoidal curve using OriginLab 2016.

NMR Spectroscopy

Structural NMR work was performed on [**Eu.1**]$^+$ (6.57 mM) in 1:1 D_2O:MeOD, pD 7.0 (500 µL). 10 µL of solution of ATP or ADP (164 mM) in D_2O, pD 7.0, was added to give 0.5 equivalents of the anion. NMR spectra were recorded on a Varian DD2-500 spectrometer with operating frequencies of 499.53 MHz for ^{1}H and 202.21 MHz for ^{31}P.

The observed chemical shifts depend both on the internuclear distance (1/r^3) and on geometry factors. Until recently, understanding of the effects of magnetic anisotropy on the observed NMR shifts in a lanthanide complex rested on the theory developed by Bleaney over 40 years ago.[8] According to Bleaney's theory, the pseudocontact shifts are described in equations (1) and (2) below,

$$\delta_{pseudo} = \frac{C_J \mu_B^2}{60\,(kT)^2}\left[\frac{(3cos^2\theta - 1)}{r^3} B_0^2 + \frac{(sin^2\theta cos2\varphi)}{r^3} B_2^2\right] \quad (1)$$

$$C_J = g_J^2 \langle J \| \alpha \| J \rangle J(J+1)(2J-1)(2J+3) \quad (2)$$

where θ, φ, and *r* define the polar coordinates and internuclear distance to the lanthanide(III) ion, C_J is the Bleaney constant, μ_B is the Bohr magneton, B_0^2 and B_2^2 are second order crystal field splitting parameters, $\langle J \| \alpha \| J \rangle$ is a numerical coefficient, *J* is the total spin orbit coupling and *g* the electron g-factor. The Bleaney constant varies with the electronic configuration of the lanthanide(III) ions, but is considered to be a property of the lanthanide only (independent of the ligand). Values of Bleaney constants for free lanthanide ions are tabulated in the literature,[8] and are often used when dealing with complexes too, under the assumption that the crystal field has a negligible effect on their value.

While Bleaney's theory has been found to conform to experimental observations in a number of systems, it contains assumptions and approximations, which may limit its applicability.

For axially symmetric complexes with a symmetry axis C_n (n>= 3), the geometric part of the right-hand term in equation (1), $(sin^2\theta cos2\varphi)$, is zero, so that part of the equation is often omitted when dealing with such complexes.

Array protocols

To each well was added 20 µL lanthanide complex solution (2× final concentration), or lanthanide complex (2× final concentration) + 10 mM $MgCl_2$, and 20 µL of an anion solution (2 mM) or buffer. The plate was then incubated for 30 minutes prior to reading. Three emission bands were read for Eu(III) complexes ΔJ = 1 (570 – 600 nm), ΔJ = 2 (601 – 631 nm) and ΔJ = 4 (675 – 710 nm)) and one time-resolved emission band (ΔJ = 2, 615 – 625 nm, integration time: 60 – 400 µs) all using λ_{exc} = 330 nm. Four emission bands were read for Tb complexes (ΔJ = 1 (479 – 507 nm), ΔJ = 2 (533 – 559 nm), ΔJ = 3 (576 – 598 nm) and ΔJ = 4 (611 – 633 nm) and one time-resolved emission band (ΔJ = 2, 510 – 550 nm, integration time: 60 – 400 µs). The values obtained for wells with no anion present were averaged over triplicate wells, all other data used was from each well individually. The percentage difference for each well for that with anion to without anion was calculated and used for statistical analysis. 9 replicates were obtained for each anion/lanthanide combination.

Microsoft Excel was used to calculate the percentage differences from no anion for each of the individual wells. Principal component analysis was carried out using IBM SPSS Statistics 23 (Factor analysis tool) in order to determine the loading values (for the first two principle components) for each of the lanthanide complex emission bands, with and without $MgCl_2$, in the combinations in Table S1. The appropriate loading values for the first two principle components were multiplied by the appropriate lanthanide complex band % emission change and summed over the anion replicate in order to calculate the first two principle components. These principle components were plotted using OriginLab 2016.

Table S1. Principle component analysis combinations calculated

**Complex**	**Anions**	**Figure showing analysis**
**[Eu.1]$^+$**, **[Eu.2]$^+$**, **[Eu.4]$^+$** and **[Tb.1]$^+$**	ATP, ADP, AMP, cAMP, GTP, GDP, GMP, CTP, CDP, CMP, UTP, UDP, UMP	S45b
**[Eu.1]$^+$**, **[Eu.2]$^+$**, **[Eu.4]$^+$** and **[Tb.1]$^+$**	ATP, ADP, AMP, cAMP, GTP, GDP, GMP, Pi	6c
**[Eu.1]$^+$**, **[Eu.2]$^+$**, **[Eu.4]$^+$** and **[Tb.1]$^+$**	ATP, ADP, AMP and cAMP	S45a
**[Eu.1]$^+$**, **[Eu.2]$^+$**, **[Eu.4]$^+$** and **[Tb.1]$^+$**	AMP, GMP, CMP and UMP	6b
**[Eu.1]$^+$**	ATP, ADP, AMP and cAMP	6a, S44a
**[Eu.2]$^+$**	ATP, ADP, AMP and cAMP	S44b
**[Eu.4]$^+$**	ATP, ADP, AMP and cAMP	S44c
**[Tb.1]$^+$**	ATP, ADP, AMP and cAMP	S44d

Mathematical description of principle component analysis

The aim of principle component analysis (PCA) is to generate linear combinations of the original data set i.e.

$X_1 = a_1A + b_1B + c_1C + \ldots$

$X_2 = a_2A + b_2B + c_2C + \ldots$

Where A, B, C etc. refer to the original data (in our analysis, this was the change in lanthanide emission band response), and a_1, b_1, c_1 etc. refer to the linear components, or loading values derived from the principle component analysis, that make X_1 then X_2 have the maximum possible variance, in the minimum possible number of equations

This equation can alternatively be written in vector formation:

$$X_1 = w_1^T a$$

Where,

$$w_1 = \begin{pmatrix} a_1 \\ b_1 \\ \ldots \\ x_1 \end{pmatrix}$$

and $\mathbf{w_1}^T$ its transpose

And, $a = \begin{pmatrix} A \\ B \\ \ldots \\ X \end{pmatrix}$

These vectors project the data onto new axes (principle component axes), with the maximum possible variance in the data.

PCA Loading values are provided in Tables S2 – S9. Analysis of the data revealed that [**Eu.4**]+ is the least important receptor for discrimination, having the smallest contribution overall to the first two principle components. It is, however, necessary for the separation of the clusters, determined by removing data from the analysis systematically. This also showed that [**Tb.1**]+ is required for the guanosine vs. adenosine separations (at least for ATP/GTP and ADP/GDP).

Table S2. Loading values for the principle components on analysis of the % change in emission responses of [**Eu.1**]+ (8 µM), [**Eu.2**]+(13 µM), [**Eu.4**]+ (10 µM) and [**Tb.1**]+ (15 µM) with the full series of nucleoside phosphate anions (NPP) anions. Conditions: 1 mM anion in 10 mM HEPES, pH 7.0

**Complex / band**	**Loading Value**	
	**PC1**	**PC2**
[Eu.1] J4	0.861	0.078
[Eu.1] J1	0.801	-0.270
[Eu.1] J2	0.899	-0.008
[Eu.1] J4 Mg	0.373	0.741
[Eu.1] J1 Mg	0.465	0.486
[Eu.1] J2 Mg	0.408	0.825
[Eu.2] J4	0.720	0.254
[Eu.2] J1	0.599	-0.454
[Eu.2] J2	0.797	-0.193
[Eu.2] J4 Mg	0.480	0.671
[Eu.2] J1 Mg	0.770	-0.139
[Eu.2] J2 Mg	0.773	0.337
[Eu.4] J4	0.478	0.273
[Eu.4] J1	0.484	-0.008
[Eu.4] J2	0.428	0.003
[Eu.4] J4 Mg	0.041	0.431
[Eu.4] J1 Mg	0.200	0.061
[Eu.4] J2 Mg	0.282	0.392
[Tb.1] J4	0.781	-0.521
[Tb.1] J3	0.767	-0.544
[Tb.1] J2	0.751	-0.550
[Tb.1] J1	0.708	-0.597
[Tb.1] J4 Mg	0.558	-0.100
[Tb.1] J3 Mg	0.552	-0.209
[Tb.1] J2 Mg	0.525	-0.275
[Tb.1] J1 Mg	0.525	-0.357
[Eu.1] TR	0.883	-0.040
[Eu.1] TR Mg	0.521	0.753
[Eu.2] TR	0.816	0.083
[Eu.2] TR Mg	0.712	0.556
[Eu.4] TR	0.869	0.121

[Eu.4] TR Mg	0.271	0.725
[Tb.1] TR	0.067	0.417
[Tb.1] TR Mg	-0.255	0.664

Table S3. Loading values for the principle components on analysis of the % change in emission responses of [**Eu.1**]$^+$ (8 µM), [**Eu.2**]$^+$(13 µM), [**Eu.4**]$^+$ (10 µM) and [**Tb.1**]$^+$ (15 µM) with ATP, ADP, AMP, cAMP, GTP, GDP, GMP and Pi. Conditions: 1 mM anion in 10 mM HEPES, pH 7.0

**Complex / band**	**Loading Value**	
	**PC1**	**PC2**
[Eu.1] J4	0.893	0.097
[Eu.1] J1	0.803	-0.089
[Eu.1] J2	0.919	0.048
[Eu.1] J4 Mg	0.597	0.414
[Eu.1] J1 Mg	0.497	0.175
[Eu.1] J2 Mg	0.626	0.574
[Eu.2] J4	0.786	0.490
[Eu.2] J1	0.690	-0.240
[Eu.2] J2	0.914	0.098
[Eu.2] J4 Mg	0.605	0.637
[Eu.2] J1 Mg	0.851	-0.045
[Eu.2] J2 Mg	0.877	0.353
[Eu.4] J4	0.430	-0.026
[Eu.4] J1	0.309	-0.411
[Eu.4] J2	0.350	-0.214
[Eu.4] J4 Mg	-0.136	0.188
[Eu.4] J1 Mg	0.074	-0.071
[Eu.4] J2 Mg	0.112	0.074
[Tb.1] J4	0.817	-0.475
[Tb.1] J3	0.797	-0.512
[Tb.1] J2	0.765	-0.531
[Tb.1] J1	0.685	-0.560
[Tb.1] J4 Mg	0.344	-0.723
[Tb.1] J3 Mg	0.316	-0.773
[Tb.1] J2 Mg	0.255	-0.834
[Tb.1] J1 Mg	0.203	-0.753
[Eu.1] TR	0.931	0.022
[Eu.1] TR Mg	0.784	0.485
[Eu.2] TR	0.890	0.261
[Eu.2] TR Mg	0.863	0.454
[Eu.4] TR	0.907	-0.075
[Eu.4] TR Mg	0.370	0.413
[Tb.1] TR	-0.003	0.735
[Tb.1] TR Mg	-0.165	0.889

Table S4. Loading values for the principle components on analysis of the % change in emission responses of [**Eu.1**]$^+$ (8 µM), [**Eu.2**]$^+$(13 µM), [**Eu.4**]$^+$ (10 µM) and [**Tb.1**]$^+$ (15 µM) with NMPs. Conditions: 1 mM anion in 10 mM HEPES, pH 7.0

**Complex / band**	**Component**	
	**PC1**	**PC2**
[Eu.1] J4	0.708	0.260
[Eu.1] J1	0.222	0.337
[Eu.1] J2	0.773	0.317
[Eu.1] J4 Mg	0.808	-0.007
[Eu.1] J1 Mg	0.722	0.312
[Eu.1] J2 Mg	0.868	0.204
[Eu.2]J4	0.277	0.773
[Eu.2]J1	0.162	0.560
[Eu.2]J2	0.190	0.756
[Eu.2]J4 Mg	0.560	0.378
[Eu.2]J1 Mg	0.371	0.271
[Eu.2]J2 Mg	0.467	0.430
[Eu.4] J4	0.353	-0.045
[Eu.4] J1	0.392	-0.054
[Eu.4] J2	0.433	-0.129
[Eu.4] J4 Mg	0.152	0.237
[Eu.4] J1 Mg	0.172	0.161
[Eu.4] J2 Mg	0.517	0.281
[Tb.1] J4	0.663	-0.578
[Tb.1] J3	0.602	-0.678
[Tb.1] J2	0.494	-0.709
[Tb.1] J1	0.453	-0.635
[Tb.1] J4 Mg	0.804	-0.447
[Tb.1] J3 Mg	0.743	-0.515
[Tb.1] J2 Mg	0.678	-0.633
[Tb.1] J1 Mg	0.628	-0.615
[Eu.1] TR	0.874	0.273
[Eu.1] TR Mg	0.927	0.159
[Eu.2] TR	0.298	0.739
[Eu.2] TR Mg	0.680	0.496
[Eu.4] TR	0.869	0.161
[Eu.4] TR Mg	0.839	0.199
[Tb.1] TR	0.002	0.417
[Tb.1] TR Mg	-0.350	0.694

Table S5. Loading values for the principle components on analysis of the % change in emission responses of [**Eu.1**]$^+$ (8 µM), [**Eu.2**]$^+$(13 µM), [**Eu.4**]$^+$ (10 µM) and [**Tb.1**]$^+$ (15 µM) with ATP, ADP, AMP and cAMP. Conditions: 1 mM anion in 10 mM HEPES, pH 7.0

**Complex / band**	**Loading Value**	
	**PC1**	**PC2**
[Eu.1] J4	0.900	0.051
[Eu.1] J1	0.885	-0.195
[Eu.1] J2	0.954	-0.022
[Eu.1] J4 Mg	0.617	0.591
[Eu.1] J1 Mg	0.444	0.297
[Eu.1] J2 Mg	0.643	0.643
[Eu.2] J4	0.844	0.441
[Eu.2] J1	0.732	-0.289
[Eu.2] J2	0.956	0.025
[Eu.2] J4 Mg	0.657	0.643
[Eu.2] J1 Mg	0.877	-0.002
[Eu.2] J2 Mg	0.900	0.320
[Eu.4] J4	0.584	0.207
[Eu.4] J1	0.358	-0.076
[Eu.4] J2	0.437	-0.108
[Eu.4] J4 Mg	-0.242	0.536
[Eu.4] J1 Mg	-0.083	0.517
[Eu.4] J2 Mg	0.063	0.634
[Tb.1] J4	0.867	-0.417
[Tb.1] J3	0.857	-0.459
[Tb.1] J2	0.837	-0.496
[Tb.1] J1	0.783	-0.514
[Tb.1] J4 Mg	0.525	-0.360
[Tb.1] J3 Mg	0.451	-0.533
[Tb.1] J2 Mg	0.381	-0.689
[Tb.1] J1 Mg	0.372	-0.648
[Eu.1] TR	0.971	-0.024
[Eu.1] TR Mg	0.781	0.552
[Eu.2] TR	0.951	0.193
[Eu.2] TR Mg	0.893	0.398
[Eu.4] TR	0.947	-0.030
[Eu.4] TR Mg	0.310	0.810
[Tb.1] TR	-0.103	0.884
[Tb.1] TR Mg	-0.161	0.901

Table S6. Loading values for the principle components on analysis of the % change in emission responses of [**Eu.1**]$^+$ (8 µM) with ATP, ADP, AMP and cAMP. Conditions: 1 mM anion in 10 mM HEPES, pH 7.0

**Emission band**	**Loading Value**	
	**PC1**	**PC2**
[Eu.1] J4	0.916	-0.317
[Eu.1] J1	0.808	-0.550
[Eu.1] J2	0.920	-0.384
[Eu.1] J4 Mg	0.783	0.484
[Eu.1] J1 Mg	0.604	0.501
[Eu.1] J2 Mg	0.845	0.509
[Eu.1] TR	0.931	-0.325
[Eu.1] TR Mg	0.918	0.304

Table S7. Loading values for the principle components on analysis of the % change in emission responses of [**Eu.2**]$^+$(13 µM) with ATP, ADP, AMP and cAMP. Conditions: 1 mM anion in 10 mM HEPES, pH 7.0

**Emission band**	**Loading Value**	
	**PC1**	**PC2**
[Eu.2] J4	0.937	-0.182
[Eu.2] J1	0.731	0.667
[Eu.2] J2	0.959	0.235
[Eu.2] J4 Mg	0.836	-0.423
[Eu.2] J1 Mg	0.922	0.287
[Eu.2] J2 Mg	0.985	-0.078
[Eu.2] TR	0.926	-0.130
[Eu.2] TR Mg	0.950	-0.271

Table S8. Loading values for the principle components on analysis of the % change in emission responses of [**Eu.4**]$^+$ (10 µM) with ATP, ADP, AMP and cAMP. Conditions: 1 mM anion in 10 mM HEPES, pH 7.0

**Emission band**	**Loading Value**	
	**PC1**	**PC2**
[Eu.4] J4	-0.200	0.650
[Eu.4] J1	-0.180	0.798
[Eu.4] J2	-0.219	0.666
[Eu.4] J4 Mg	0.681	-0.237
[Eu.4] J1 Mg	0.926	0.085
[Eu.4] J2 Mg	0.928	0.255
[Eu.4] TR	-0.115	0.833
[Eu.4] TR Mg	0.865	0.417

Table S9. Loading values for the principle components on analysis of the % change in emission responses of [**Tb.1**]$^+$ (15 µM) with ATP, ADP, AMP and cAMP. Conditions: 1 mM anion in 10 mM HEPES, pH 7.0

**Complex / band**	**Loading Value**	
	**PC1**	**PC2**
[Tb.1] J4	0.881	-0.391
[Tb.1] J3	0.898	-0.381
[Tb.1] J2	0.916	-0.349
[Tb.1] J1	0.881	-0.399
[Tb.1] J4 Mg	0.782	0.476
[Tb.1] J3 Mg	0.843	0.493
[Tb.1] J2 Mg	0.887	0.427
[Tb.1] J1 Mg	0.848	0.416
[Tb.1] TR	-0.684	0.109
[Tb.1] TR Mg	-0.752	0.116

2.2. Emission Spectra of Ln(III) complexes alone

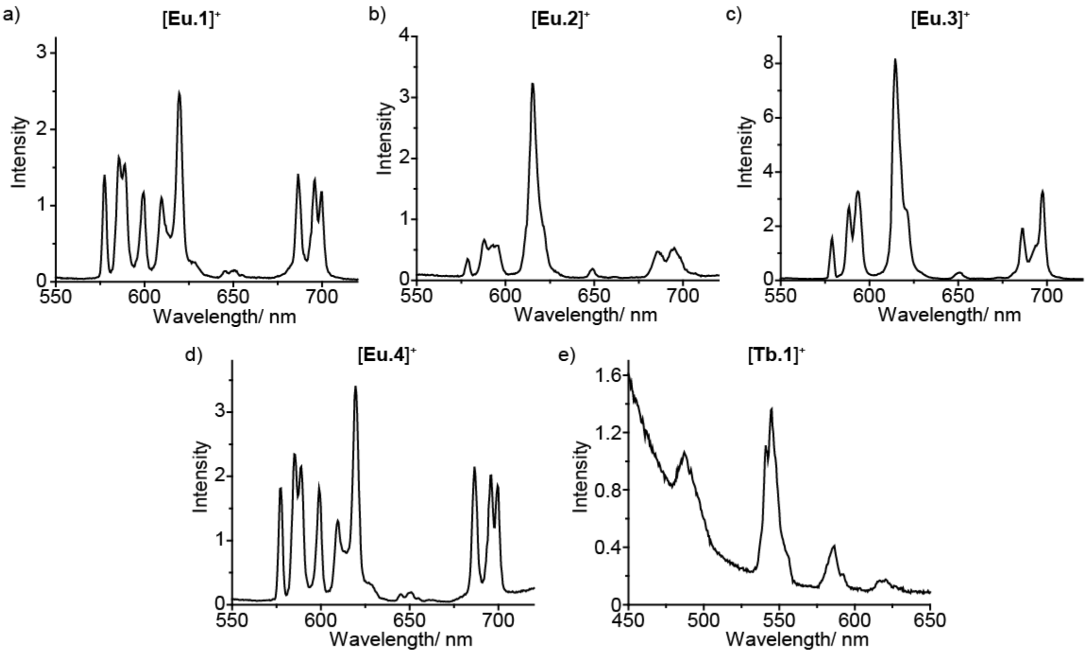

Figure S1. Emission spectra of the Ln(III) complexes in the absence of anion. a) [**Eu.1**]$^+$ (8 µM), b) [**Eu.2**]$^+$ (13 µM), c) [**Eu.3**]$^+$ (6 µM), d) [**Eu.4**]$^+$ (10 µM) and e) [**Tb.1**]$^+$ (15 µM). Conditions: 10 mM HEPES, pH 7.0, λ_{exc} = 330 nm, 0.1 absorbance.

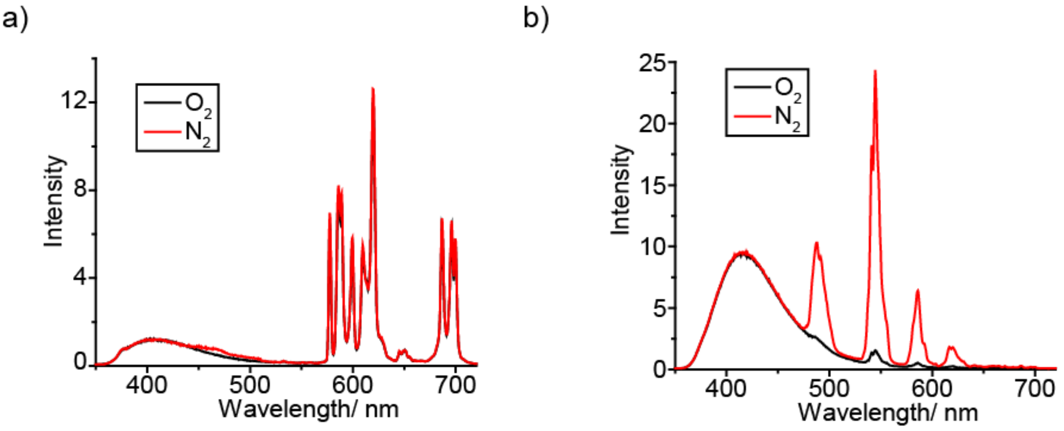

Figure S2. Change in emission spectra of [**Eu.1**]$^+$ and [**Tb.1**]$^+$ when bubbled with O_2 and N_2 gas for 5 minutes. a) [**Eu.1**]$^+$ (8 µM), b) [**Tb.1**]$^+$ (15 µM). Conditions: 10 mM HEPES, pH 7.0, λ_{exc} = 330 nm

a) b)

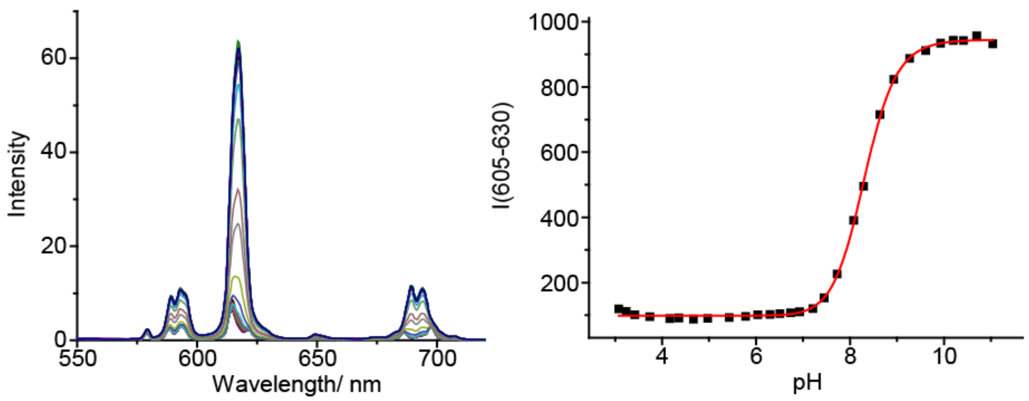

Figure S3. Effect of pH on the emission of [**Eu.3**]$^+$. a) Change in emission spectra b) Change in intensity of the Δ*J* = 2 band as a function of pH. Conditions: 6 μM [**Eu.3**]$^+$ in 10 mM HEPES, λ_{exc} = 330 nm

Table S10: Emission lifetimes of complexes **[Eu.1]**$^+$, **[Eu.2]**$^+$ and **[Eu.4]**$^+$ measured in 10 mM HEPES in the absence and presence of ATP, ADP and AMP (1 mM, unless stated otherwise). The number of inner sphere water molecules (hydration state, *q*) was determined using the modified Horrocks equation. [9]

**Sample**	**τ(H_2O) / ms**	**τ(D_2O) / ms**	***q***
**[Eu.1]**$^+$	0.48	1.39	1.2
**[Eu.1]**$^+$ + ATP	1.14	1.68	-0.03
**[Eu.1]**$^+$ + ADP	1.18	1.68	-0.06
**[Eu.1]**$^+$ + 5 mM AMP	0.84	1.64	0.29
**[Eu.2]**$^+$	0.56	1.22	0.8
**[Eu.2]**$^+$ + ATP	1.13	1.71	-0.01
**[Eu.2]**$^+$ + ADP	1.12	1.70	-0.01
**[Eu.2]**$^+$ + 5 mM AMP	0.53	1.44	0.76
**[Eu.4]**$^+$	0.53	1.44	0.94
**[Eu.4]**$^+$ + ATP	0.95	1.70	0.16
**[Eu.4]**$^+$ + ADP	0.95	1.68	0.15
**[Eu.4]**$^+$ + 5 mM AMP	0.65	1.61	0.68

2.3. Anion screening (emission spectra)

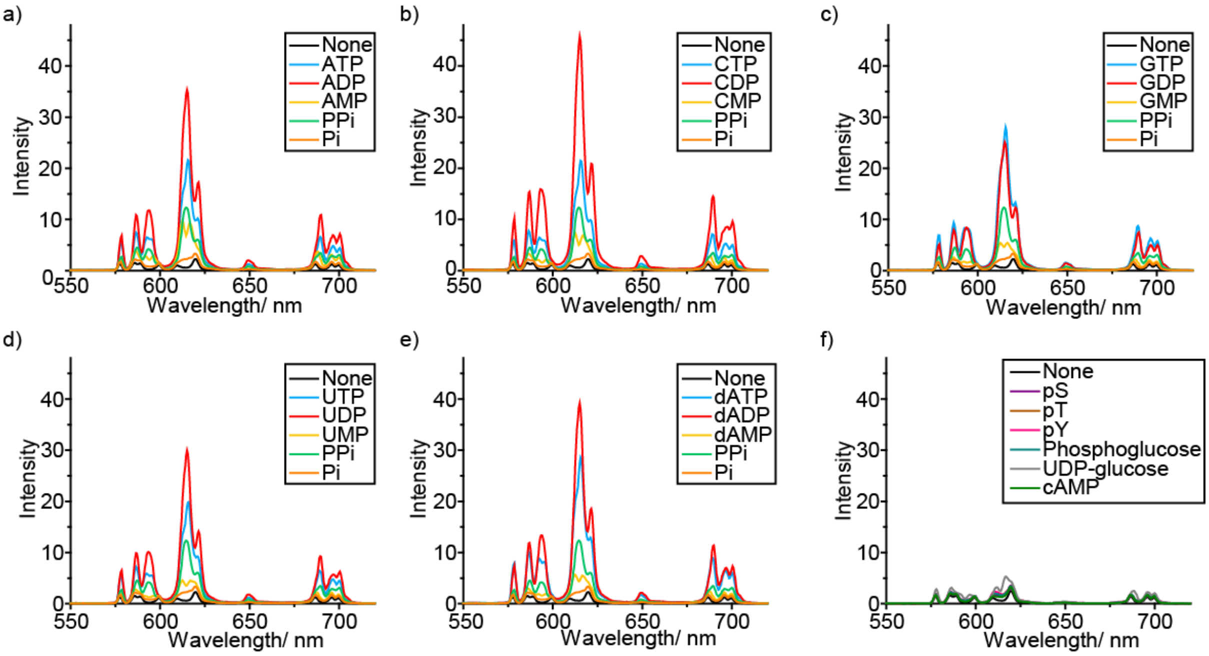

Figure S4. Effect of addition of various phosphoanions (1 mM) on the emission spectra of [**Eu.1**]$^{+}$ (8 μM) in 10 mM HEPES, pH 7.0, λ_{exc} = 330 nm

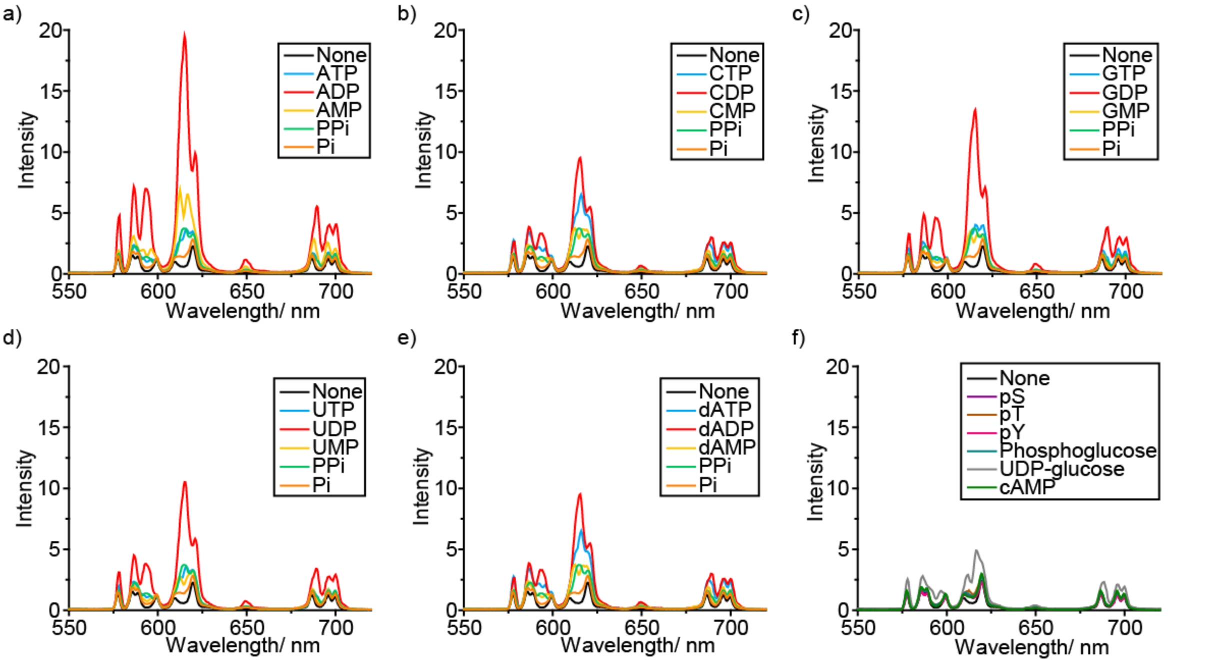

Figure S5. Effect of addition of various phosphoanions (1 mM) on the emission spectra of [**Eu.1**]$^{+}$ (8 μM) in the presence of $MgCl_2$ (5 mM), in 10 mM HEPES, pH 7.0, λ_{exc} = 330 nm

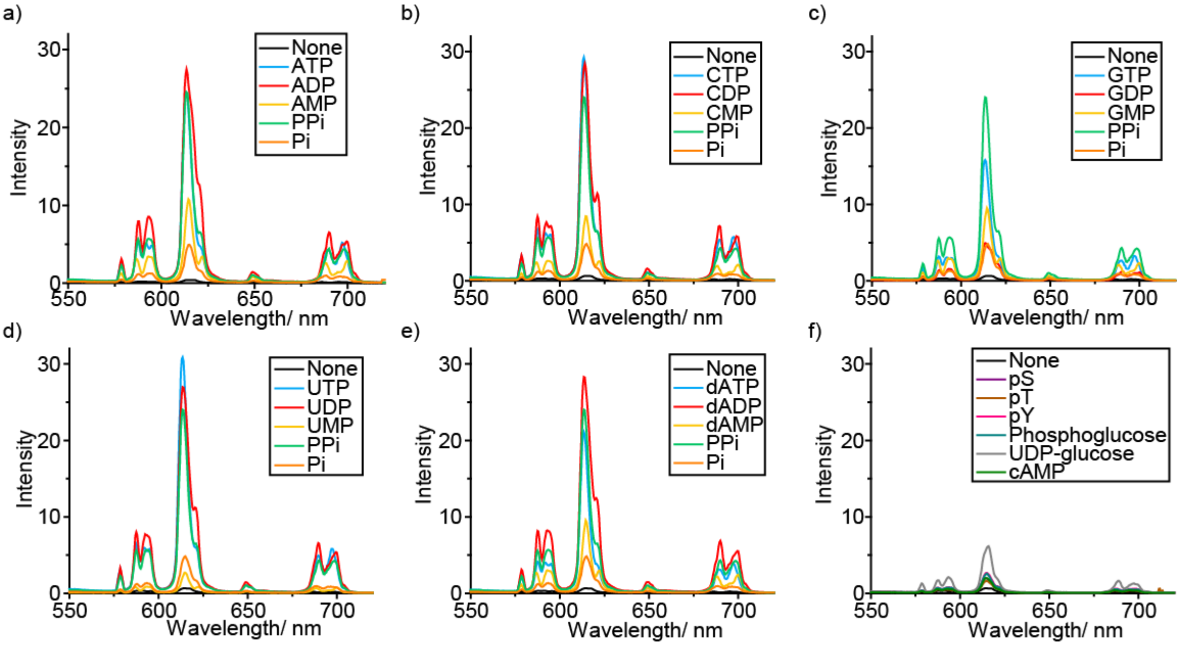

Figure S6. Effect of addition of various phosphoanions (1 mM) on the emission spectra of [**Eu.2**]$^+$ (13 μM) in 10 mM HEPES, pH 7.0, λ_{exc} = 330 nm

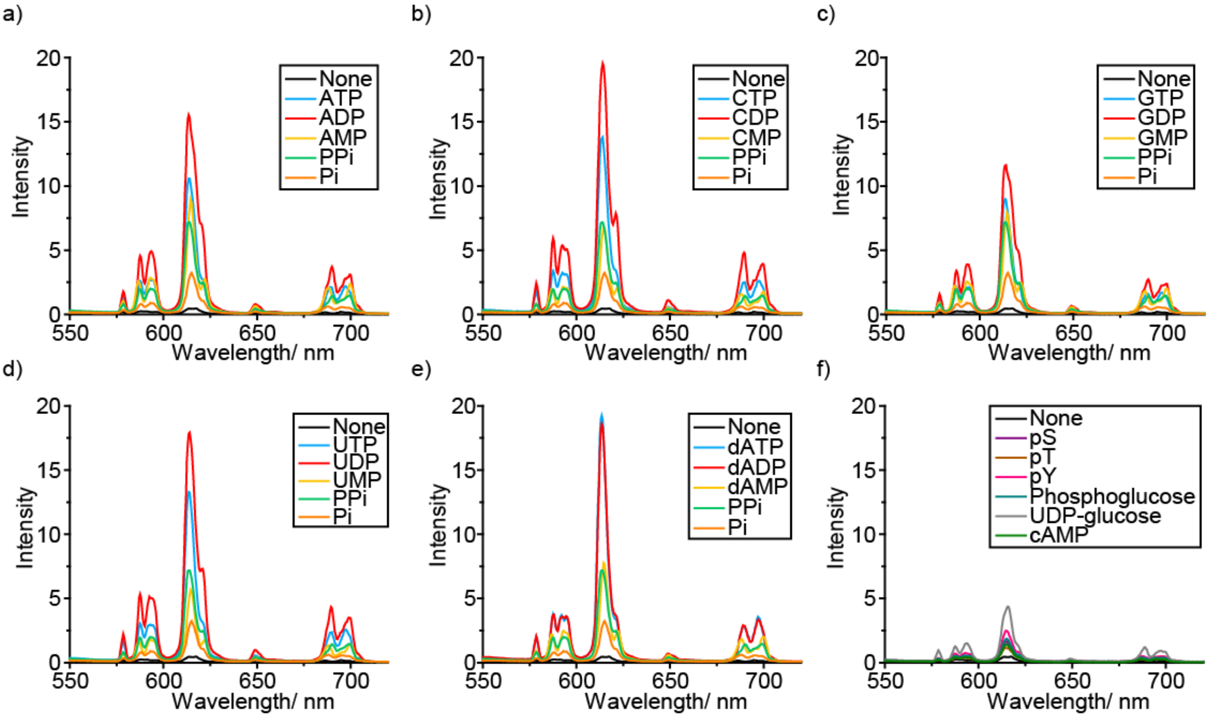

Figure S7. Effect of addition of various phosphoanions (1 mM) on the emission spectra of [**Eu.2**]$^+$ (13 μM) in the presence of $MgCl_2$ (5 mM), in 10 mM HEPES, pH 7.0, λ_{exc} = 330 nm

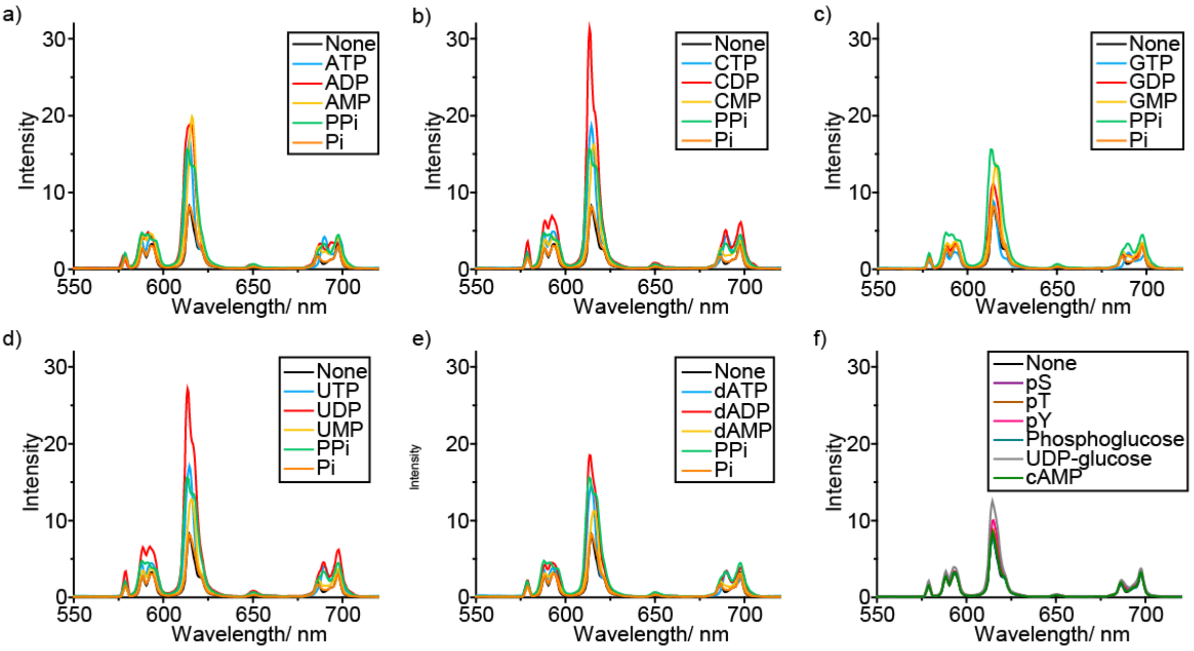

Figure S8. Effect of addition of various phosphoanions (1 mM) on the emission spectra of [**Eu.3**]$^+$ (6 μM) in 10 mM HEPES, pH 7.0, λ_{exc} = 330 nm

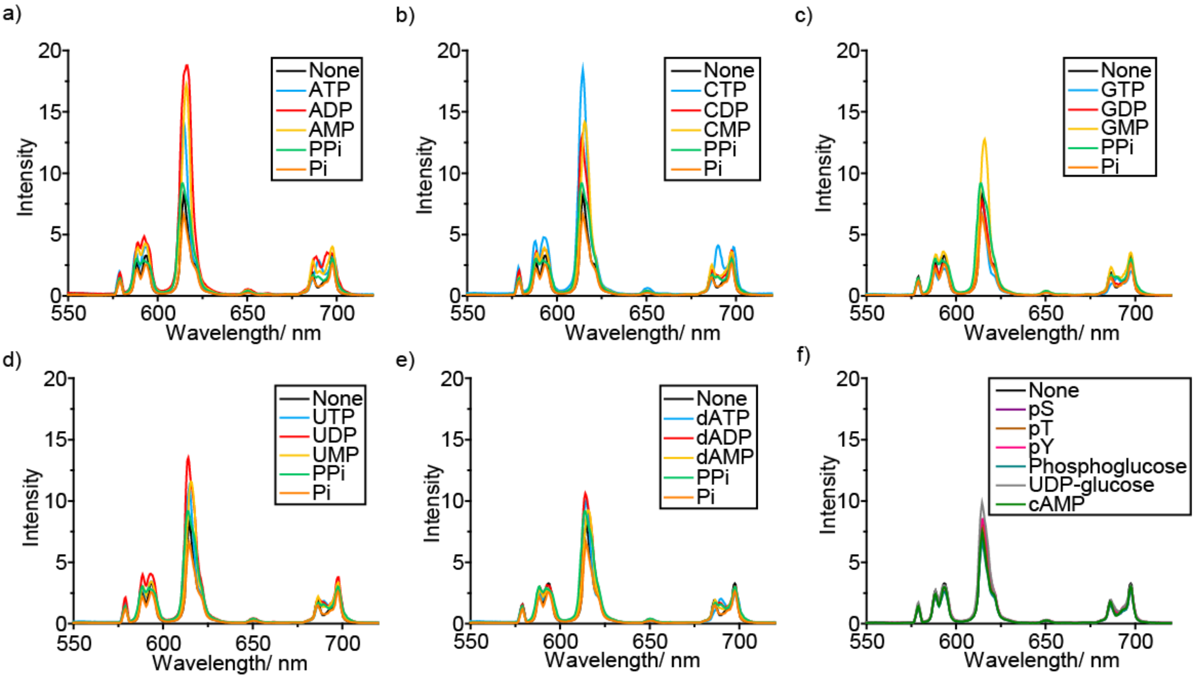

Figure S9. Effect of addition of various phosphoanions (1 mM) on the emission spectra of [**Eu.3**]$^+$ (6 μM) in the presence of $MgCl_2$ (5 mM), in 10 mM HEPES, pH 7.0, λ_{exc} = 330 nm

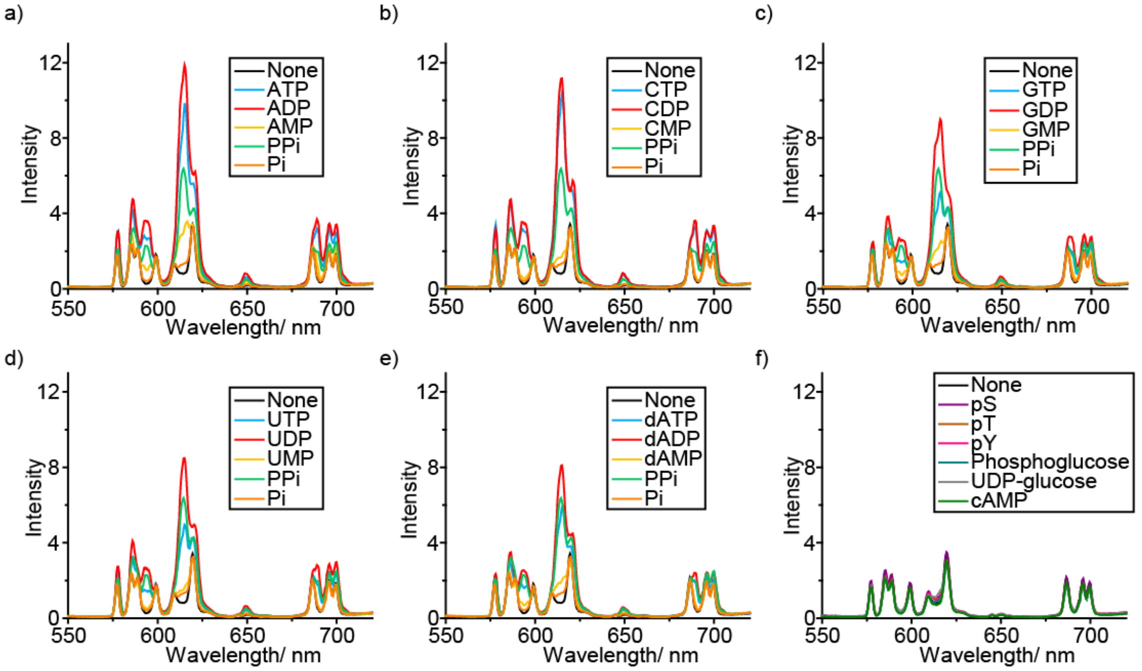

Figure S10. Effect of addition of various phosphoanions (1 mM) on the emission spectra of [**Eu.4**]$^{+}$ (10 µM) in 10 mM HEPES, pH 7.0, λ_{exc} = 330 nm

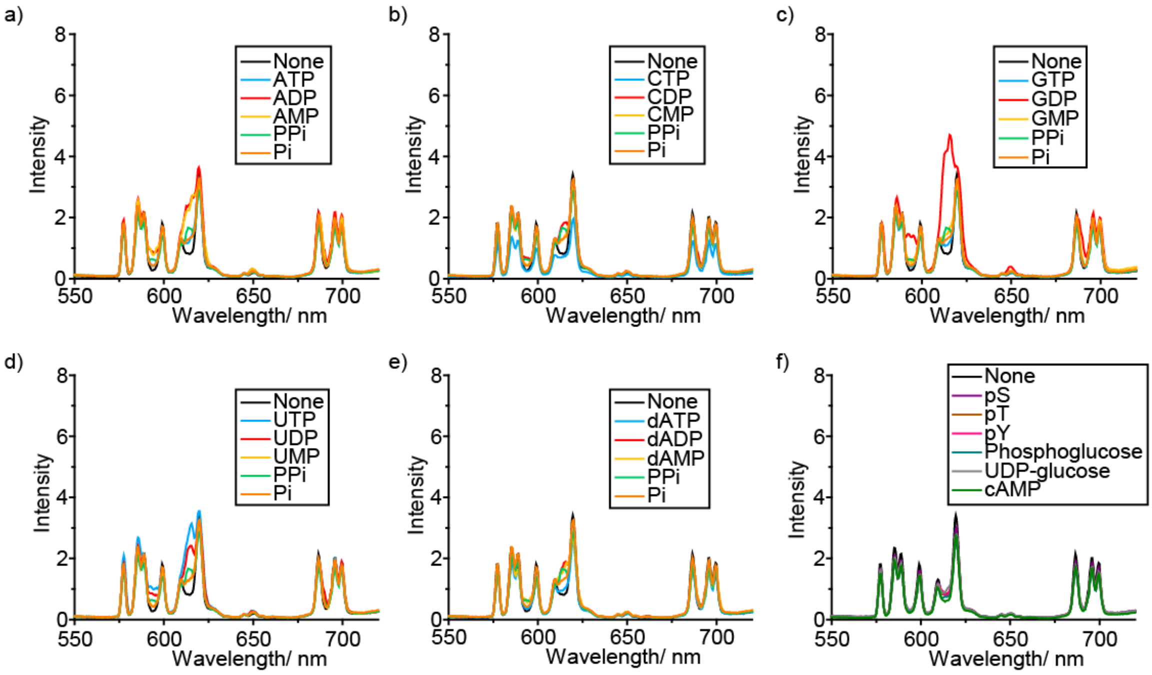

Figure S11. Effect of addition of various phosphoanions (1 mM) on the emission spectra of [**Eu.4**]$^{+}$ (10 µM) in the presence of $MgCl_2$ (5 mM), in 10 mM HEPES, pH 7.0, λ_{exc} = 330 nm

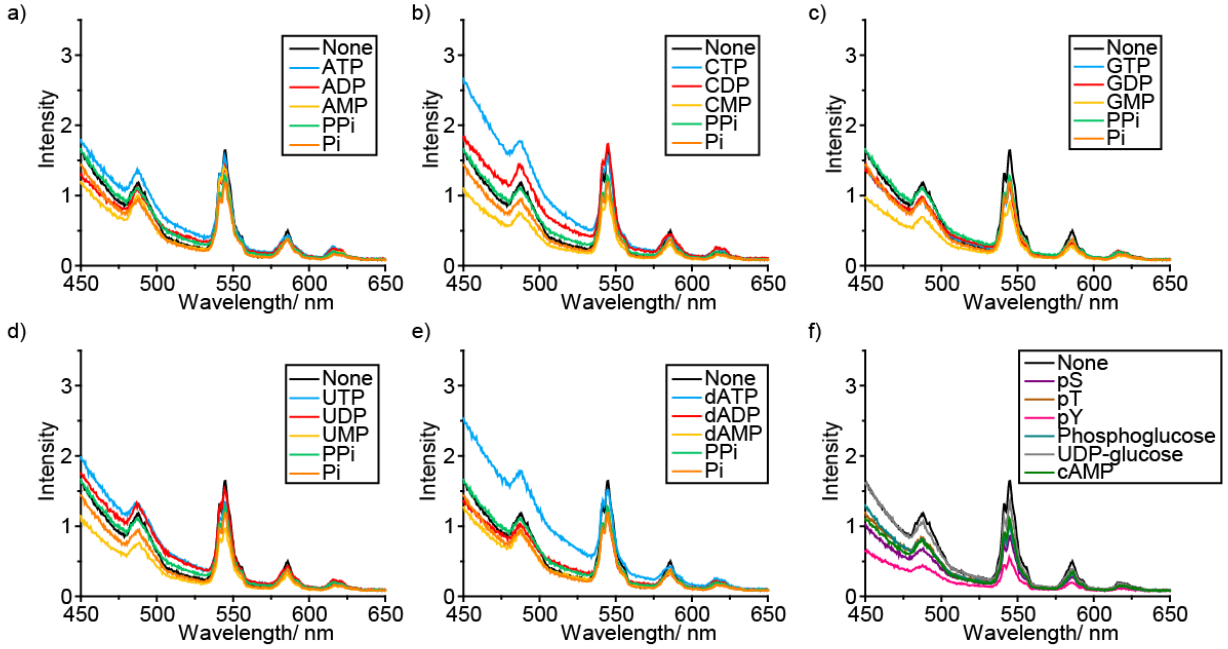

Figure S12. Effect of addition of various phosphoanions (1 mM) on the emission spectra of [**Tb.1**]$^{+}$ (15 μM) in 10 mM HEPES, pH 7.0, λ_{exc} = 330 nm

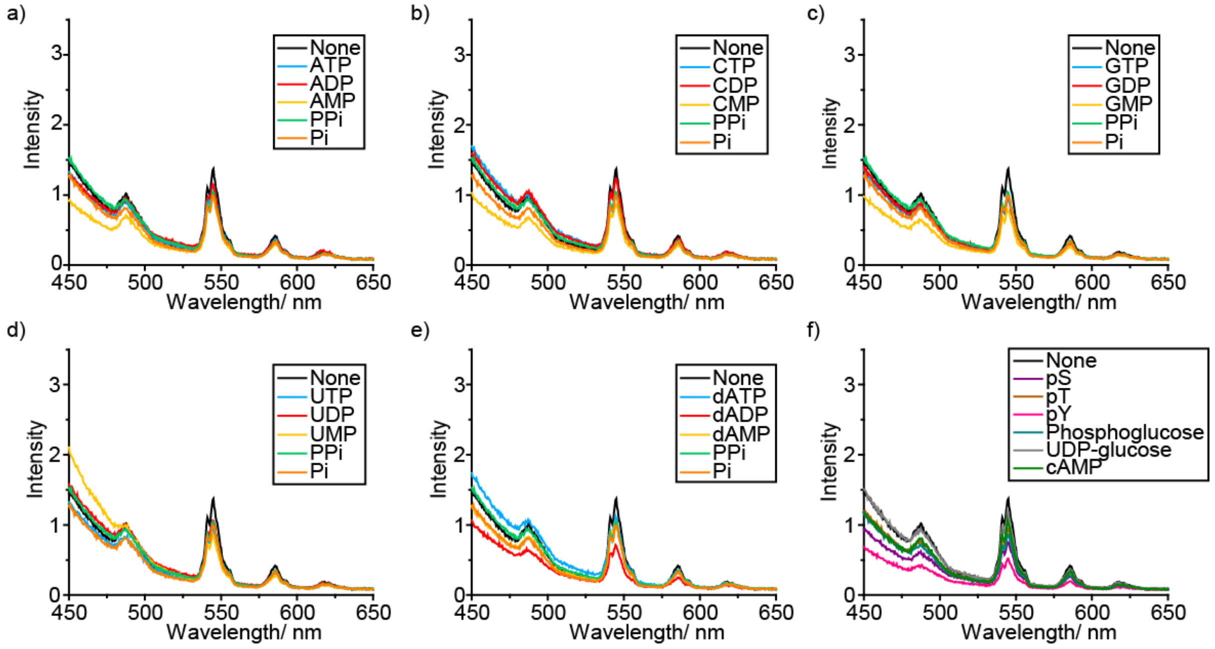

Figure S13. Effect of addition of various phosphoanions (1 mM) on the emission spectra of [**Tb.1**]$^{+}$ (15 μM) in the presence of $MgCl_2$ (5 mM), in 10 mM HEPES, pH 7.0, λ_{exc} = 330 nm

Adenosine series: comparison of emission spectral bands

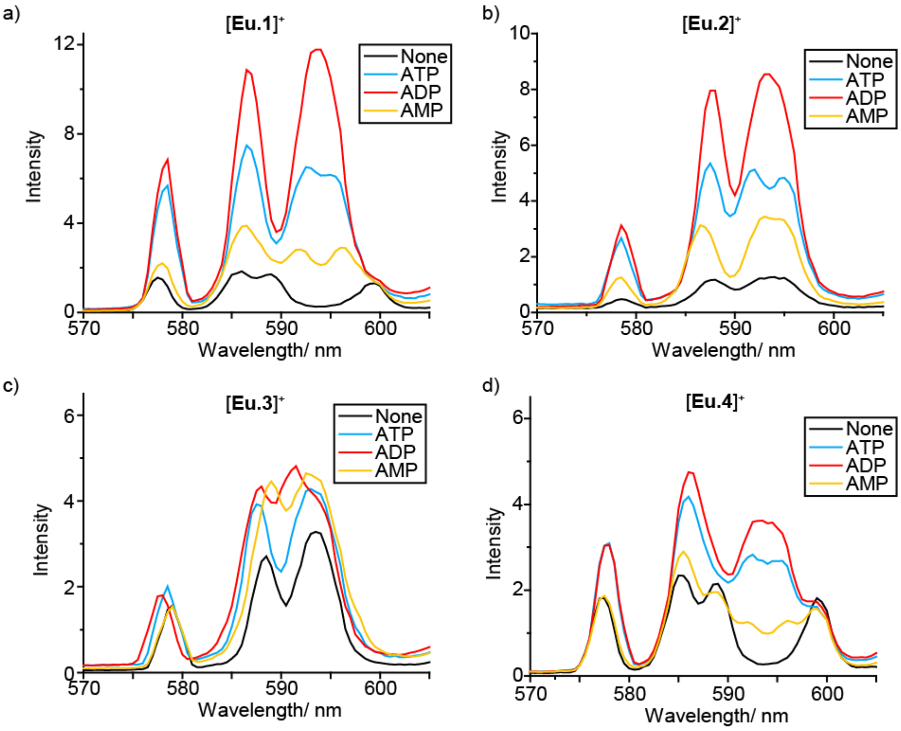

Figure S14. Effect of addition of ATP, ADP and AMP (1 mM) on the Δ*J* = 1 band (570 – 605 nm) of the emission spectra of [**Eu.1**]$^+$ (8 μM, a), [**Eu.2**]$^+$ (13 μM, b), [**Eu.3**]$^+$ (6 μM, c) and [**Eu.4**]$^+$ (10 μM, d) in 10 mM HEPES, pH 7.0, λ_{exc} = 330 nm

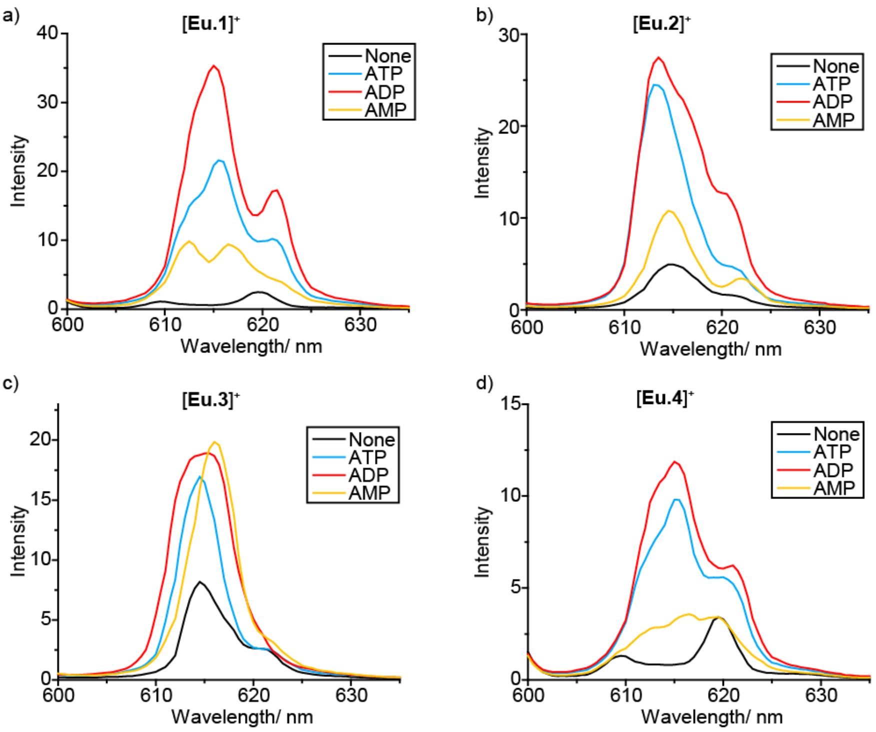

Figure S15. Effect of addition of ATP, ADP and AMP (1 mM) on the Δ*J* = 2 band (600 – 635 nm) of the emission spectra of [**Eu.1**]$^+$ (8 μM, a), [**Eu.2**]$^+$ (13 μM, b), [**Eu.3**]$^+$ (6 μM, c) and [**Eu.4**]$^+$ (10 μM, d) in 10 mM HEPES, pH 7.0, λ_{exc} = 330 nm

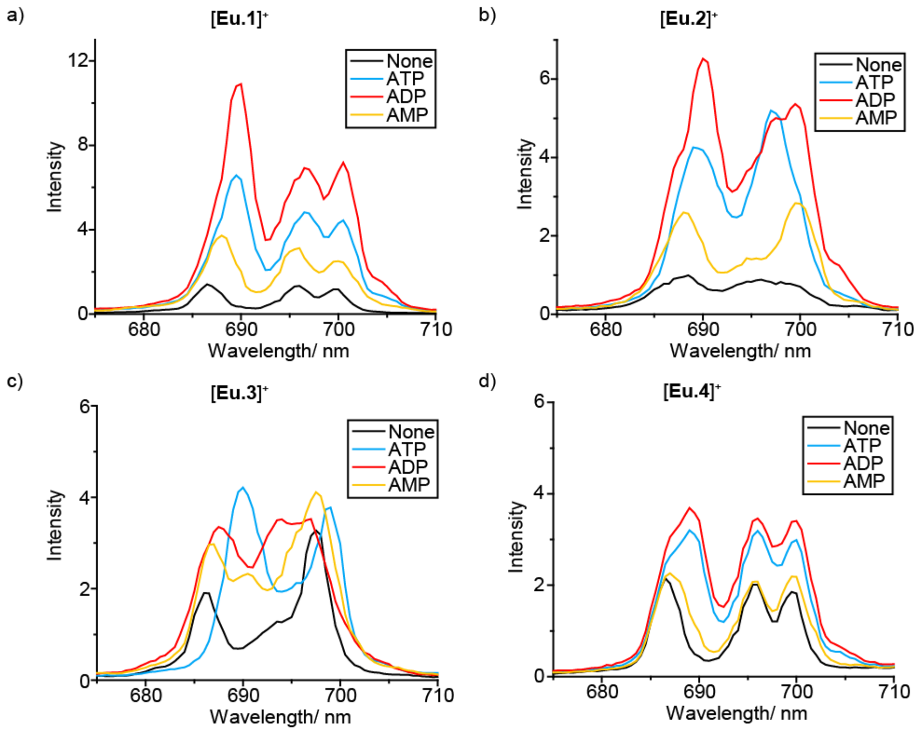

Figure S16. Effect of addition of ATP, ADP and AMP (1 mM) on the Δ*J* = 4 band (675 – 710 nm) of the emission spectra of [**Eu.1**]$^+$ (8 µM, a), [**Eu.2**]$^+$ (13 µM, b), [**Eu.3**]$^+$ (6 µM, c) and [**Eu.4**]$^+$ (10 µM, d) in 10 mM HEPES, pH 7.0, λ_{exc} = 330 nm

2.4. Anion screening (bar charts displaying changes in emission intensity)

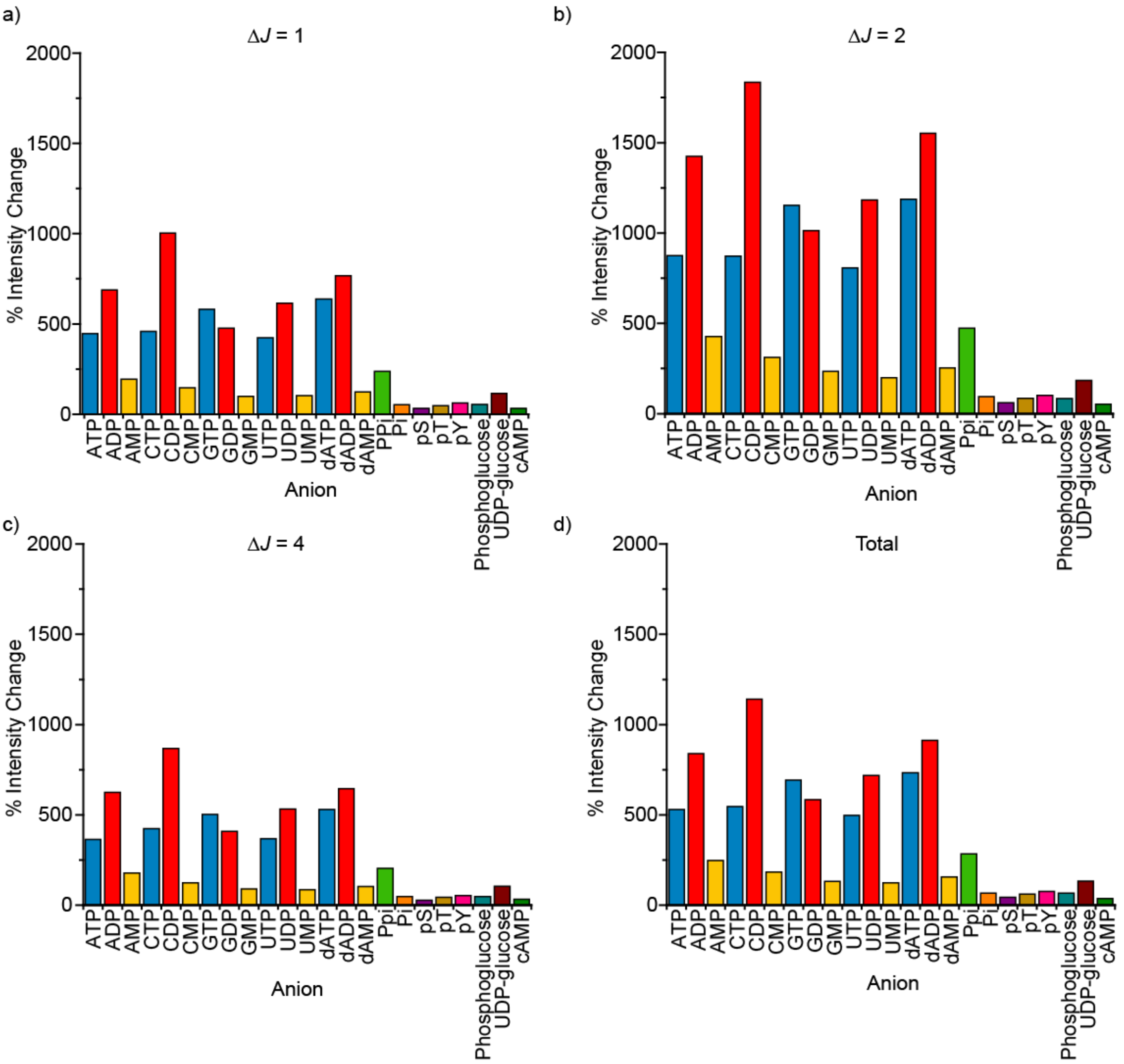

Figure S17. Effect of addition of various phosphoanions (1 mM) on the emission bands of [**Eu.1**]$^+$ (8 μM), calculated as the percentage intensity change in the presence of anion compared to without the anion. a) Δ*J* = 1 (585 – 600 nm), b) Δ*J* = 2 (605 – 630 nm), c) Δ*J* = 4 (680 – 705 nm), and d) total emission (550 – 720 nm). Conditions: 10 mM HEPES, pH 7.0, λ_{exc} = 330 nm.

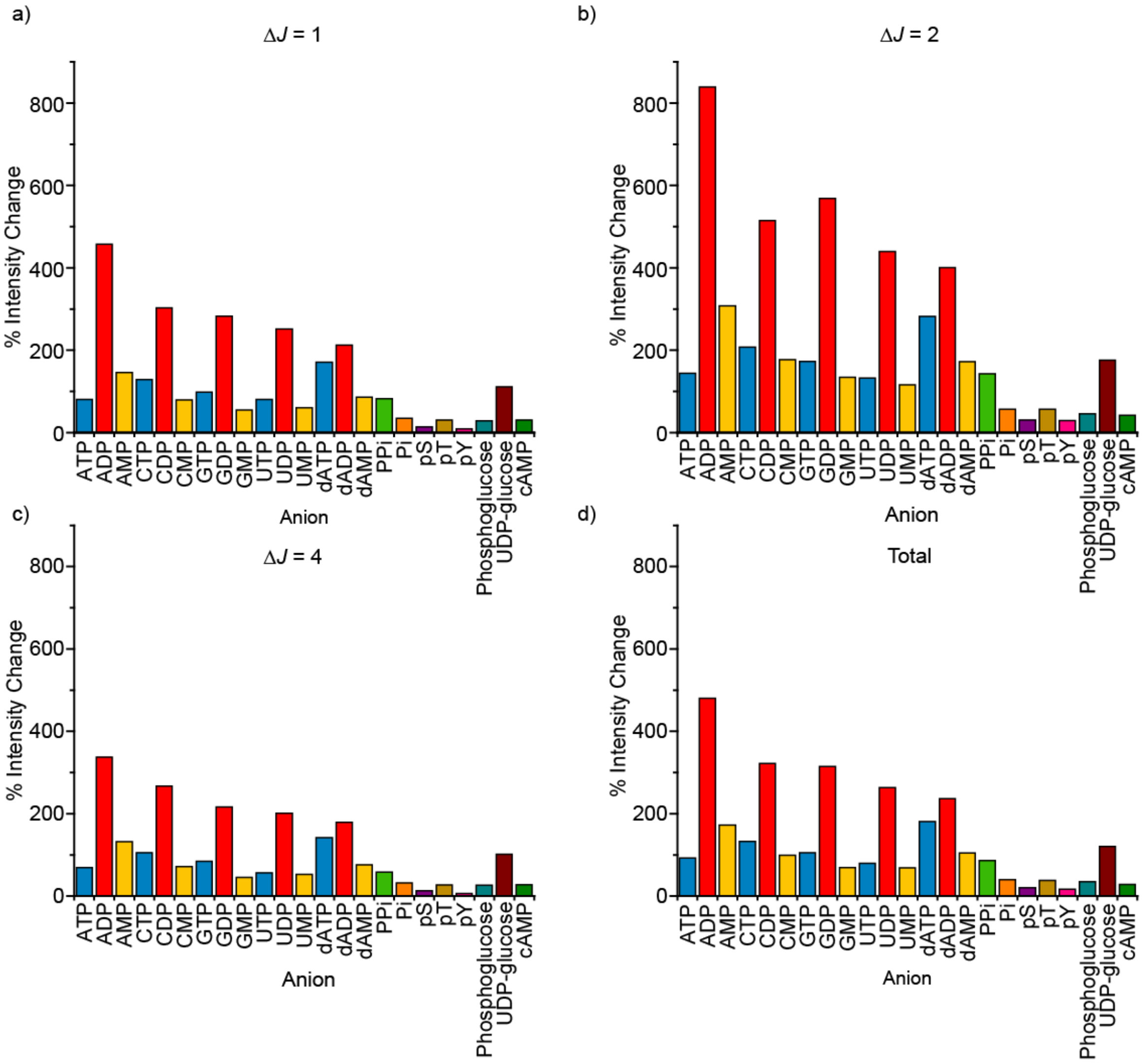

Figure S18. Effect of addition of various phosphoanions (1 mM) on the emission bands of [**Eu.1**]$^+$ (8 μM) in the presence of $MgCl_2$ (5 mM), calculated as the percentage intensity change in the presence of anion compared to without the anion. a) Δ*J* = 1 (585 – 600 nm), b) Δ*J* = 2 (605 – 630 nm), c) Δ*J* = 4 (680 – 705 nm), and d) total emission (550 – 720 nm). Conditions: 10 mM HEPES, pH 7.0, λ_{exc} = 330 nm.

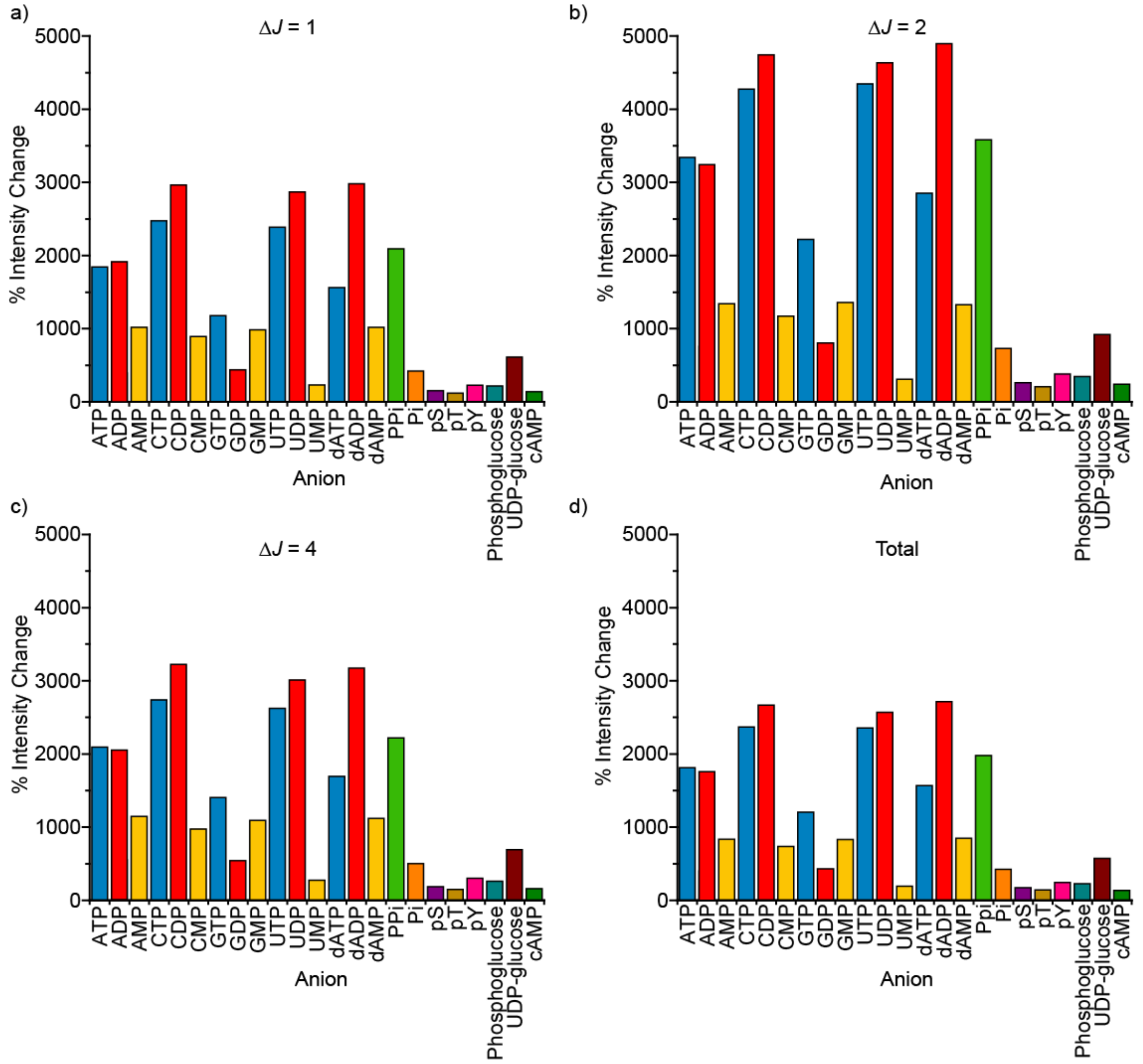

Figure S19. Effect of addition of various phosphoanions (1 mM) on the emission bands of [**Eu.2**]$^+$ (13 μM), calculated as the percentage intensity change in the presence of anion compared to without the anion. a) Δ*J* = 1 (585 – 600 nm), b) Δ*J* = 2 (605 – 630 nm), c) Δ*J* = 4 (680 – 705 nm), and d) total emission (550 – 720 nm). Conditions: 10 mM HEPES, pH 7.0, λ_{exc} = 330 nm.

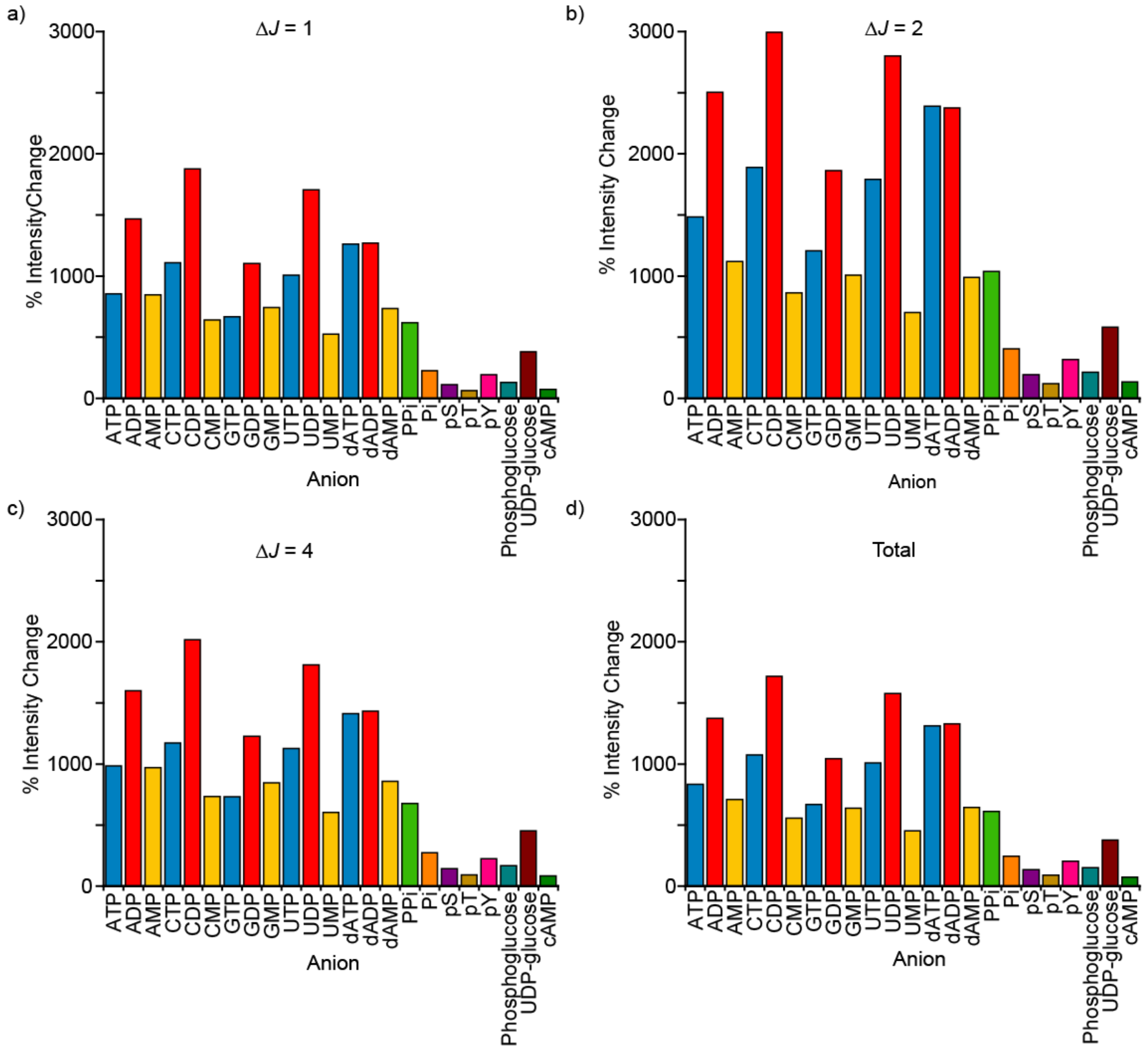

Figure S20. Effect of addition of various phosphoanions (1 mM) on the emission bands of [**Eu.2**]$^+$ (13 µM) in the presence of $MgCl_2$ (5 mM), calculated as the percentage intensity change in the presence of anion compared to without the anion. a) Δ*J* = 1 (585 – 600 nm), b) Δ*J* = 2 (605 – 630 nm), c) Δ*J* = 4 (680 – 705 nm), and d) total emission (550 – 720 nm). Conditions: 10 mM HEPES, pH 7.0, λ_{exc} = 330 nm.

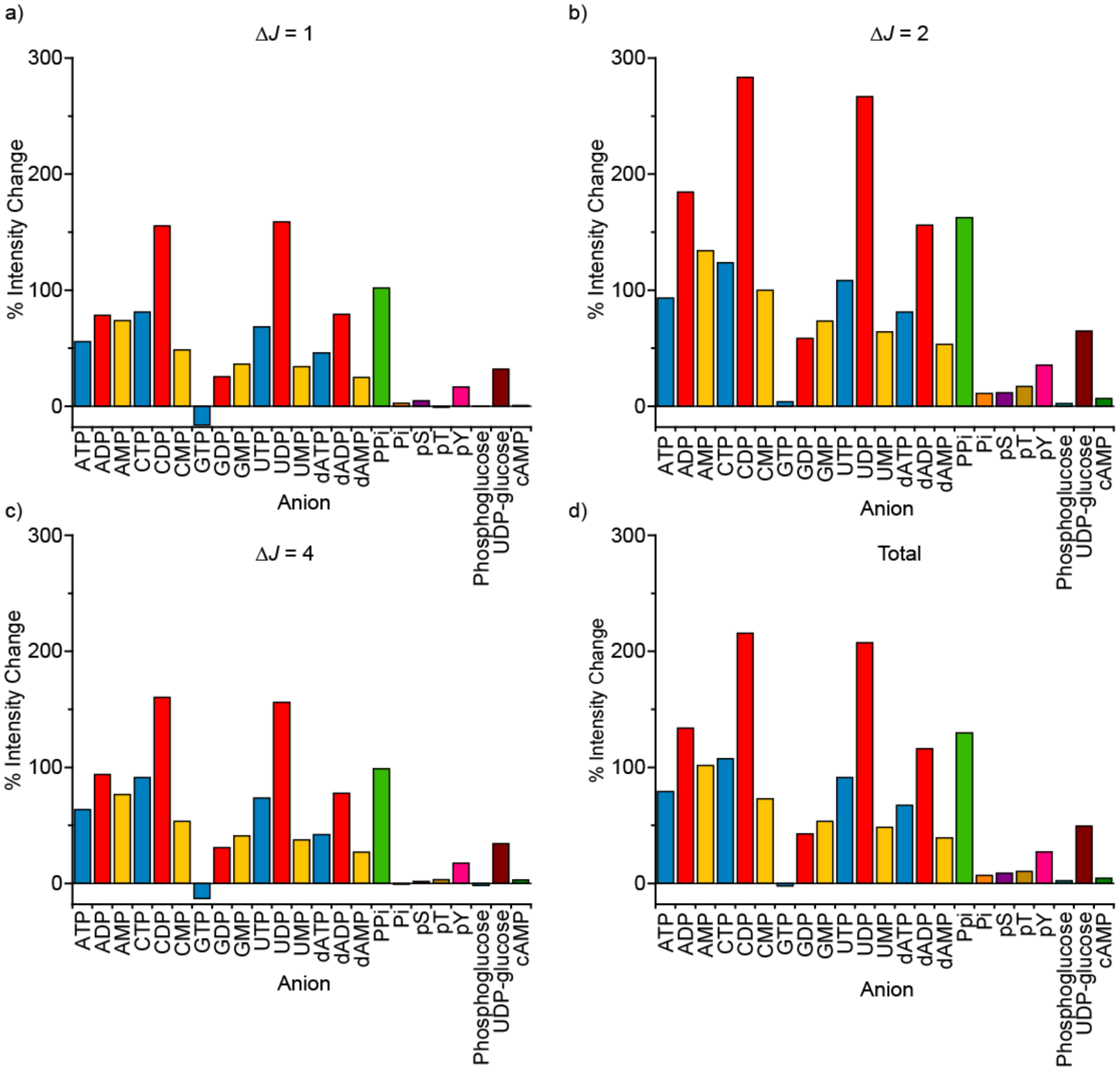

Figure S21. Effect of addition of various phosphoanions (1 mM) on the emission bands of [**Eu.3**]$^+$ (6 μM), calculated as the percentage intensity change in the presence of anion compared to without the anion. a) Δ*J* = 1 (585 – 600 nm), b) Δ*J* = 2 (605 – 630 nm), c) Δ*J* = 4 (680 – 705 nm), and d) total emission (550 – 720 nm). Conditions: 10 mM HEPES, pH 7.0, λ_{exc} = 330 nm.

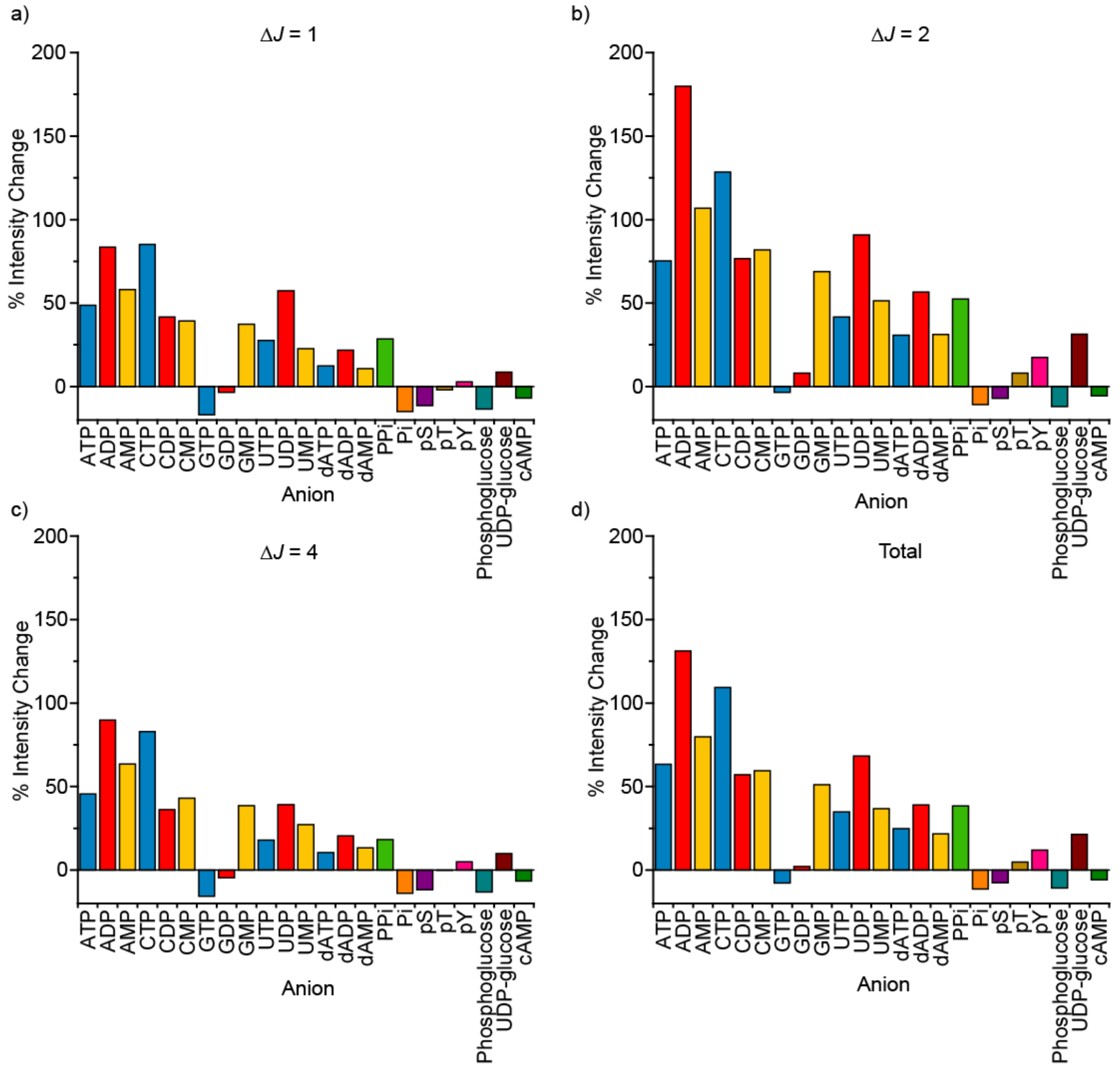

Figure S22. Effect of addition of various phosphoanions (1 mM) on the emission bands of [**Eu.3**]$^+$ (6 μM) in the presence of $MgCl_2$ (5 mM), calculated as the percentage intensity change in the presence of anion compared to without the anion. a) Δ*J* = 1 (585 – 600 nm), b) Δ*J* = 2 (605 – 630 nm), c) Δ*J* = 4 (680 – 705 nm), and d) total emission (550 – 720 nm). Conditions: 10 mM HEPES, pH 7.0, λ_{exc} = 330 nm.

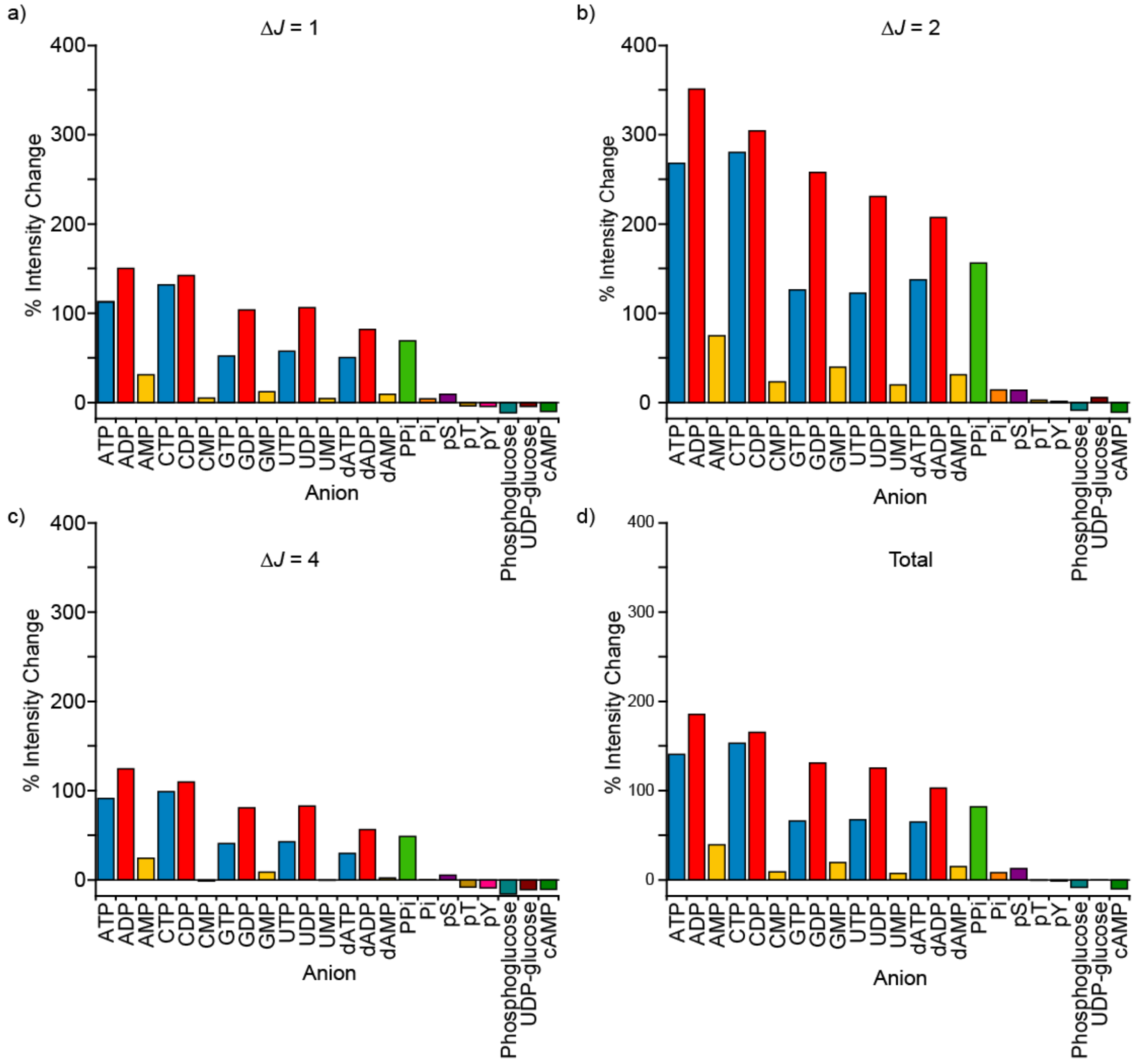

Figure S23. Effect of addition of various phosphoanions (1 mM) on the emission bands of [**Eu.4**]$^+$ (10 μM), calculated as the percentage intensity change in the presence of anion compared to without the anion. a) Δ*J* = 1 (585 – 600 nm), b) Δ*J* = 2 (605 – 630 nm), c) Δ*J* = 4 (680 – 705 nm), and d) total emission (550 – 720 nm). Conditions: 10 mM HEPES, pH 7.0, λ_{exc} = 330 nm.

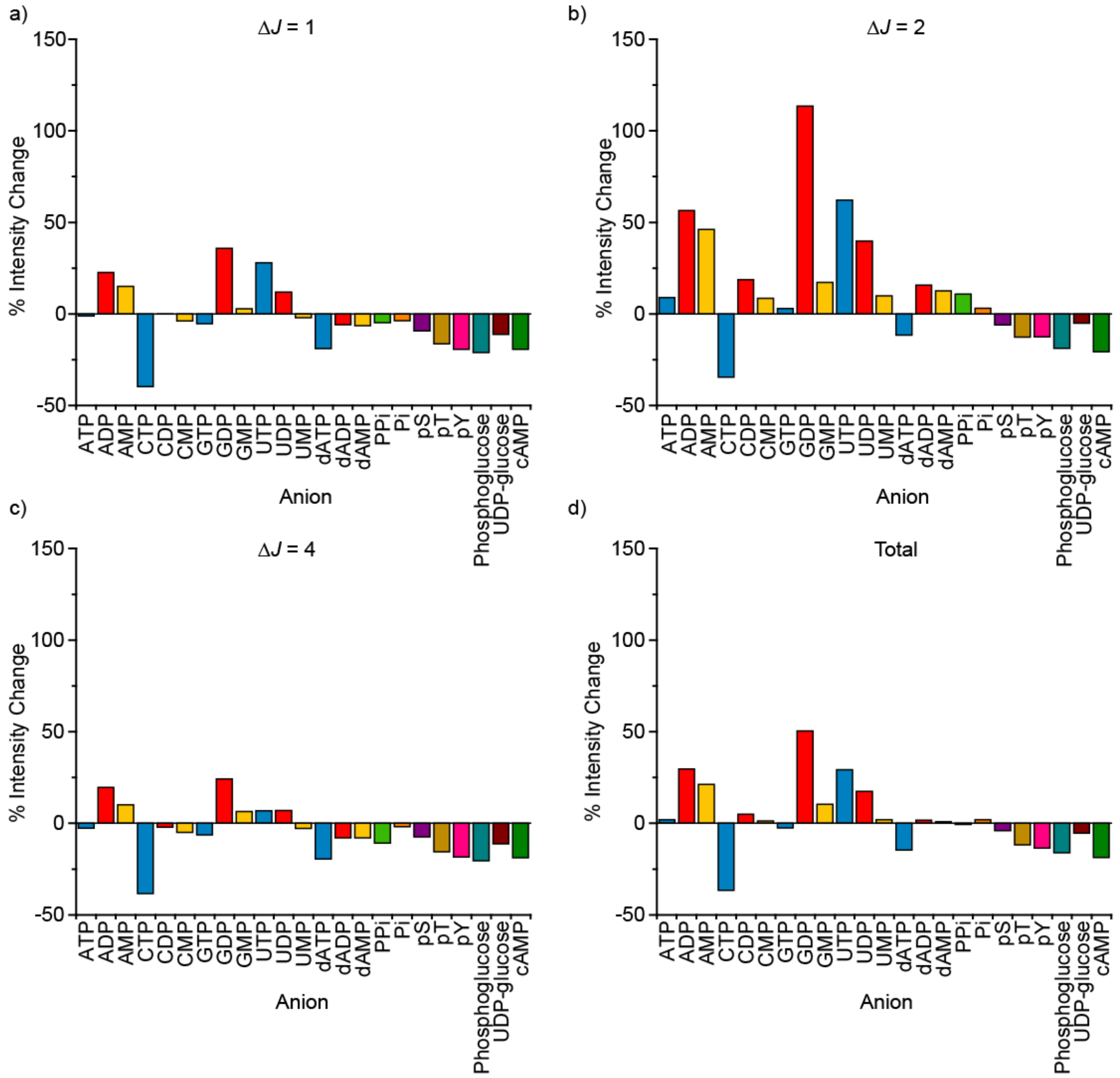

Figure S24. Effect of addition of various phosphoanions (1 mM) on the emission bands of [**Eu.4**]$^+$ (10 µM) in the presence of $MgCl_2$ (5 mM), calculated as the percentage intensity change in the presence of anion compared to without the anion. a) Δ*J* = 1 (585 – 600 nm), b) Δ*J* = 2 (605 – 630 nm), c) Δ*J* = 4 (680 – 705 nm), and d) total emission (550 – 720 nm). Conditions: 10 mM HEPES, pH 7.0, λ_{exc} = 330 nm.

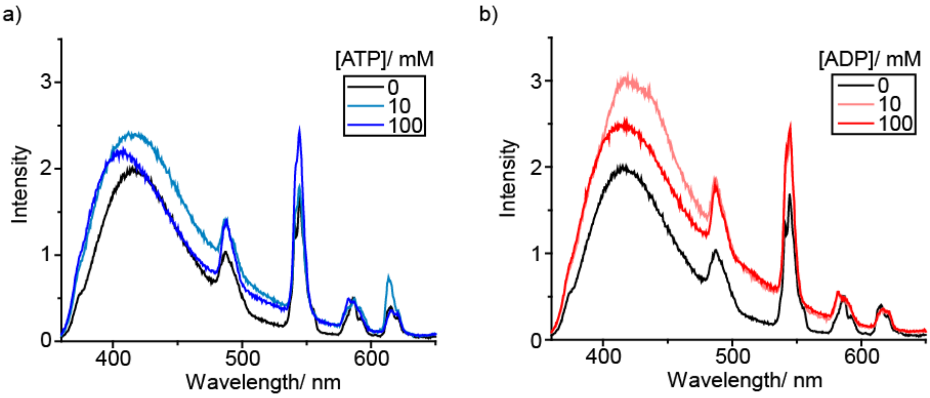

Figure S25. Effect of addition of 10 and 100 mM ATP (a) and ADP (b) on the emission spectra of [**Tb.1**]$^{+}$ (15 μM). Conditions: 10 mM HEPES, pH 7.0, λ_{exc} = 330 nm

2.5. Stability of [Eu.1]$^{+}$ and [Eu.3]$^{+}$ with and without selected anions

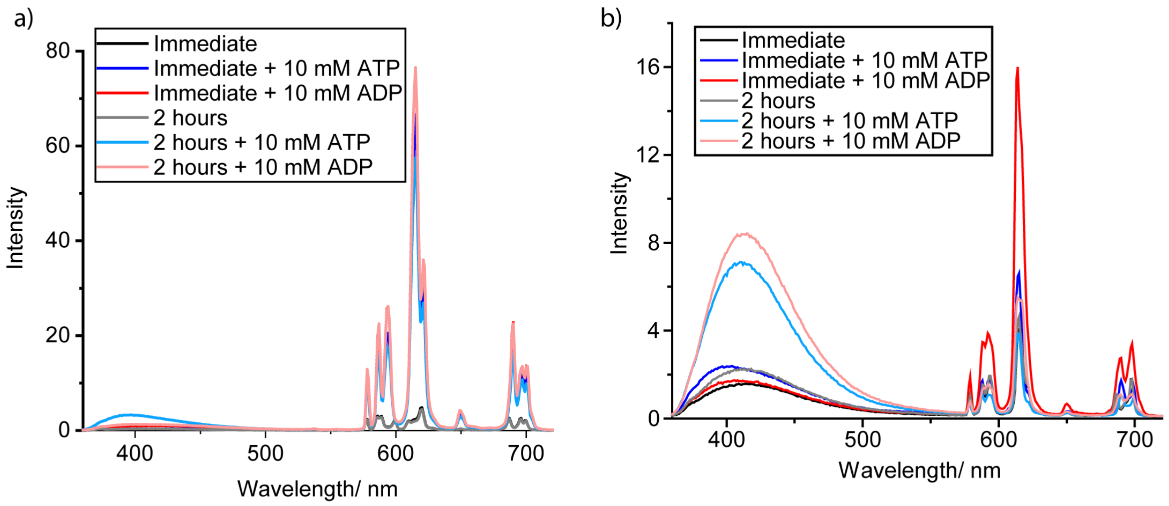

Figure S26. Effect of addition of ATP and ADP (10 mM) and 2 hour incubation on the emission of (a) [**Eu.1**]$^{+}$ and (b) **[Eu.3]**$^{+}$. Conditions: 10 mM HEPES, pH 7.0, λ_{exc} = 330 nm, [**Eu.1**]$^{+}$ (8 μM), [**Eu.3**]$^{+}$ (6 μM).

2.6. Anion binding titrations in buffered aqueous solution

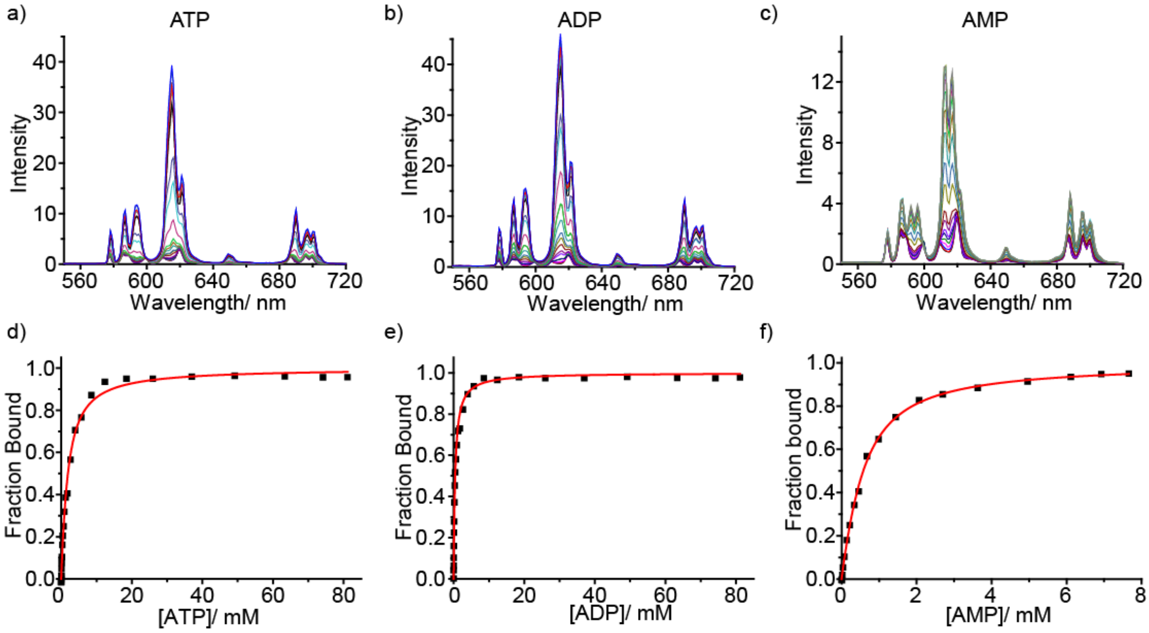

Figure S27. Titration of ATP (a, d), ADP (b, e) and AMP (c, f) into [**Eu.1**]$^{+}$ (8 µM) in the presence of $MgCl_2$ (5 mM). a), b) and c) Change in emission spectra; d), e) and f) Fit of the fraction bound (measured using the ratio of intensity at 605-630/580-600 nm) to a 1:1 binding isotherm. Conditions: 10 mM HEPES, pH 7.0, λ_{exc} = 330 nm

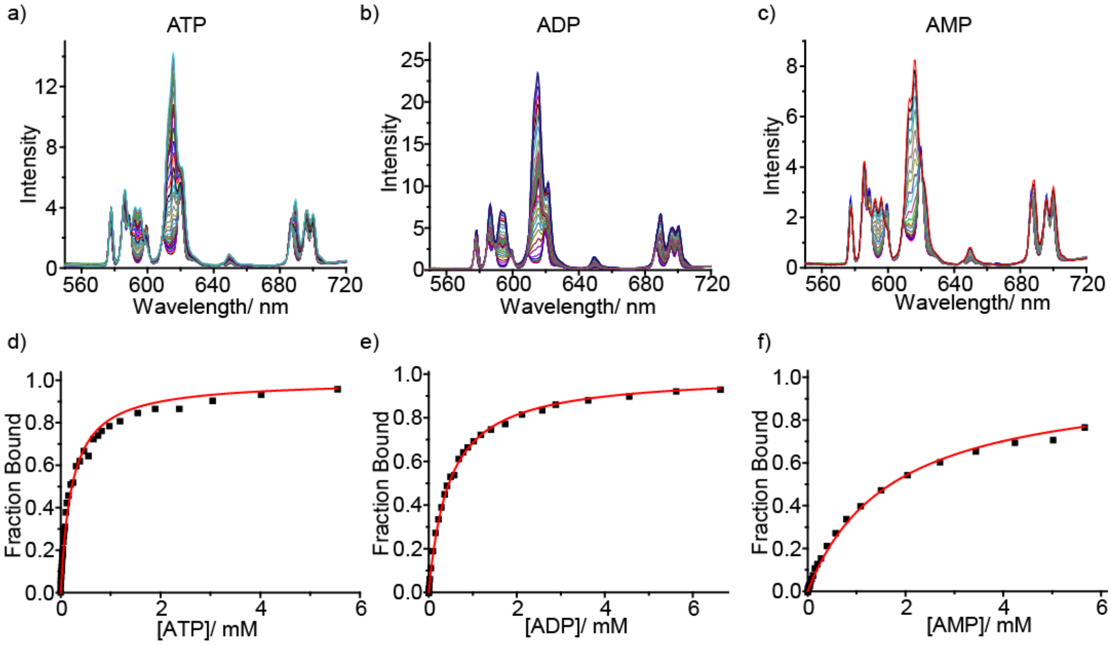

Figure S28. Titration of ATP (a, d), ADP (b, e) and AMP (c, f) into [**Eu.4**]$^{+}$ (10 µM). a), b) and c) Change in emission spectra; d), e) and f) Fit of the fraction bound (measured using the ratio of intensity at 605-630/580-600 nm) to a 1:1 binding isotherm. Conditions: 10 mM HEPES, pH 7.0, λ_{exc} = 330 nm

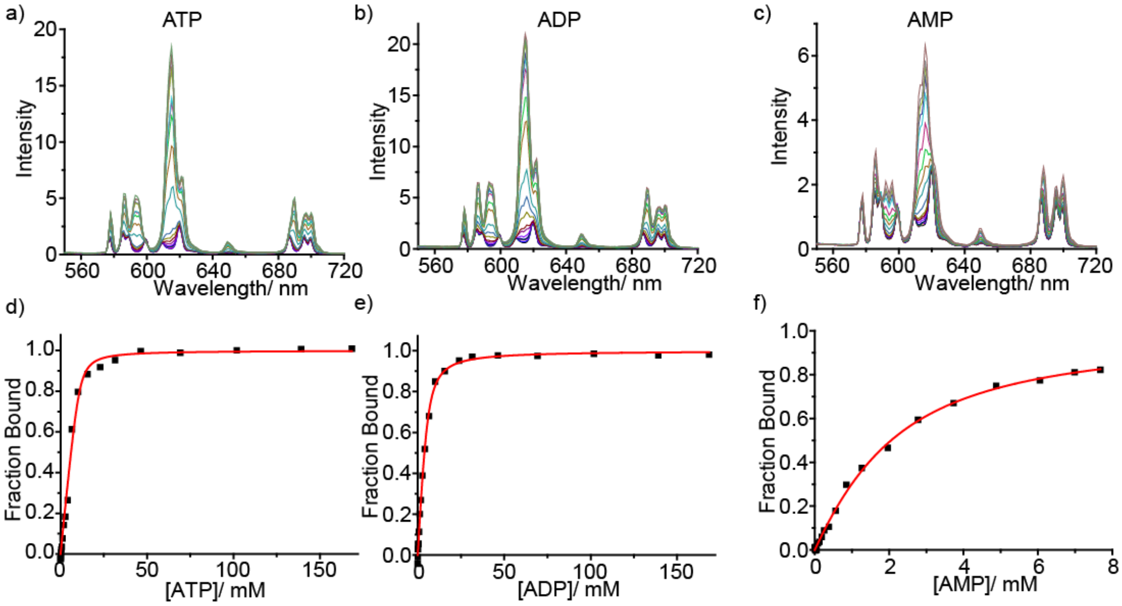

Figure S29. Titration of ATP (a, d), ADP (b, e) and AMP (c, f) into [**Eu.4**]$^+$ (10 µM) in the presence of $MgCl_2$ (5 mM). a), b) and c) Change in emission spectra; d), e) and f) Fit of the fraction bound (measured using the ratio of intensity at 605-630/580-600 nm) to a 1:1 binding isotherm. Conditions: 10 mM HEPES, pH 7.0, λ_{exc} = 330 nm

Data obtained from fitting of the titration of [Eu.1]$^+$ with ATP to a 2:1 (and 1:1) binding model using Bindfit, can be accessed here:

2:1 host-guest model:

http://app.supramolecular.org/bindfit/view/99da6060-9983-4e28-ba52-d4f474ce7673

1:1 host-guest model:

http://app.supramolecular.org/bindfit/view/19a32a56-3ce5-4fd6-9a31-d0d9f8331a1b

2.7. X-ray crystallography

Crystal data, atomic coordinates, geometry, etc, are given in the tables at the end of this document. The structure was solved[10] and refined[11] routinely except as detailed in the following text. $C_{37}H_{61}EuN_8O_{15}\cdot 7(H_2O)$. Half of the molecule comprises the asymmetric unit because the molecule is located on a two-fold axis running through Eu(1) and O(4). One unique water molecule of crystallisation was well-behaved and located in a general position with H atoms located. Another water molecule was located on a special position, but H atoms could not be located. The remaining three unique water molecules were modelled as having the oxygen atom disordered over two

positions with H atoms not located. The formate ion is disordered with C(19) and O(5) modelled as disordered over two, equally-occupied, sets of positions determined by the symmetry.

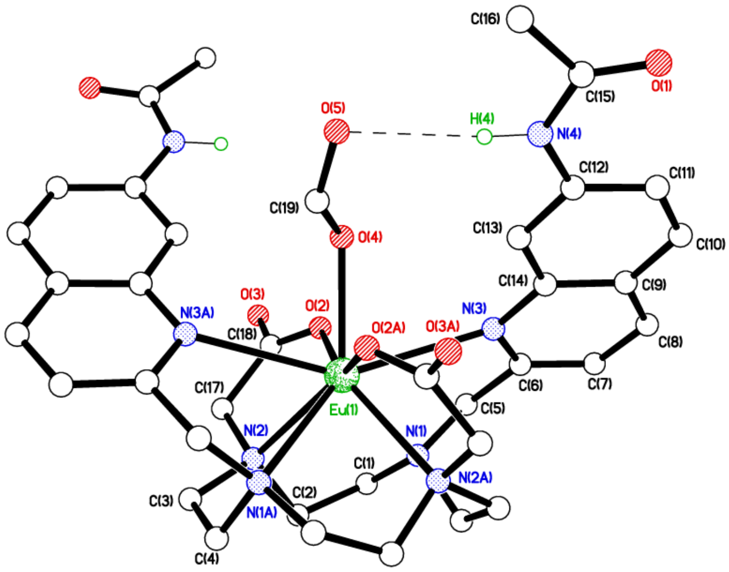

Figure S30. Molecular structure of [**Eu.1**]$^{+}$ showing the atomic numbering scheme.

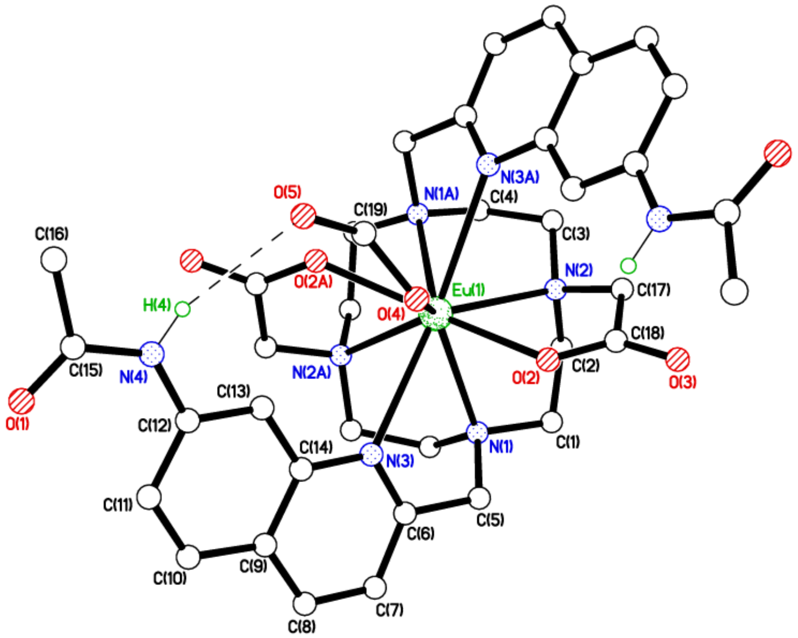

Figure S31. Alternative view of the molecular structure of [**Eu.1**]$^{+}$.

Packing: Pairs of molecules embrace and there are supramolecular contacts via N–H···O hydrogen bonds to the terminal formate O(5) atom. Thus atom O(5) accepts two H bonds, one intramolecular and one intermolecular. Neighbouring pairs of embraced molecules also form two pairs of rather ungainly π···π interactions between their aromatic ring systems. These are not quite parallel so the

inter-atom separations vary between *ca*. 3.38 Å at the short end between C(10) and C(11′), to values greater than 4 Å where the ring systems diverge. Pairs of embraced molecules are then linked into layers and these are linked via channels/zones of largely disordered water molecules, which H-bond with each other and suitably located donors and acceptors on the Eu complexes.

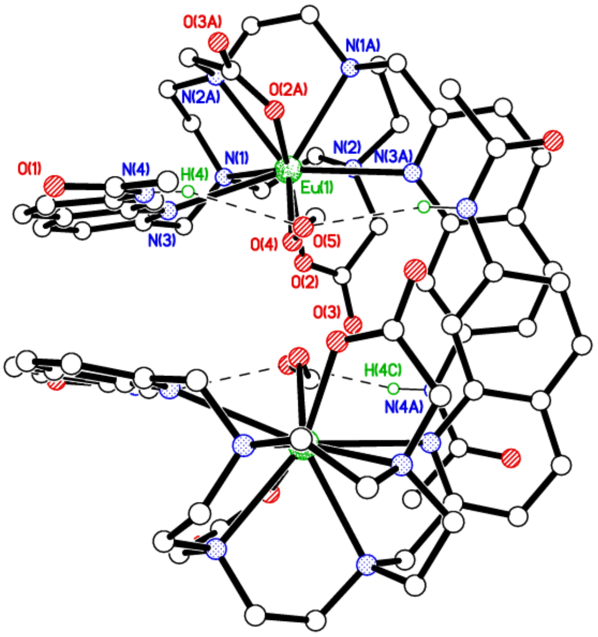

Figure S32. Two embraced molecules of [Eu.1]$^+$ with pairs of H-bonded N–H···O (formate) interactions and pairs of perpendicular, off-set, π···π interactions (edge-on, left and plan-view, right).

2.7. NMR Spectra

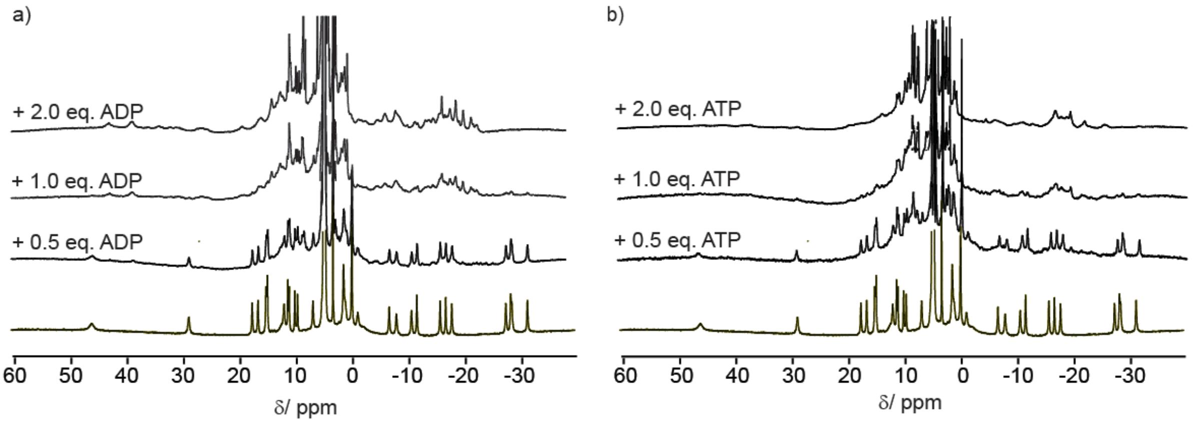

Figure S33. ^{1}H NMR spectra of [**Eu.1**]$^+$ (6.57 mM) with added ADP (a) and ATP (b). Conditions: 1:1 D_2O:MeOD, pD 7.0, 499.53 MHz

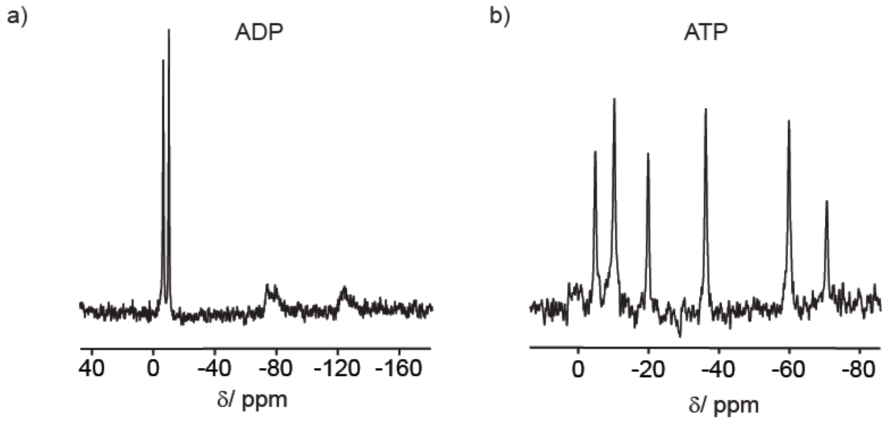

Figure S34. ^{31}P NMR spectra of [**Eu.1**]$^{+}$ (6.57 mM) with (a) added ADP (6.57 mM), and (b) ATP (6.57 mM). Conditions: 1:1 D_2O:MeOD, pD 7.0, 202.21 MHz.

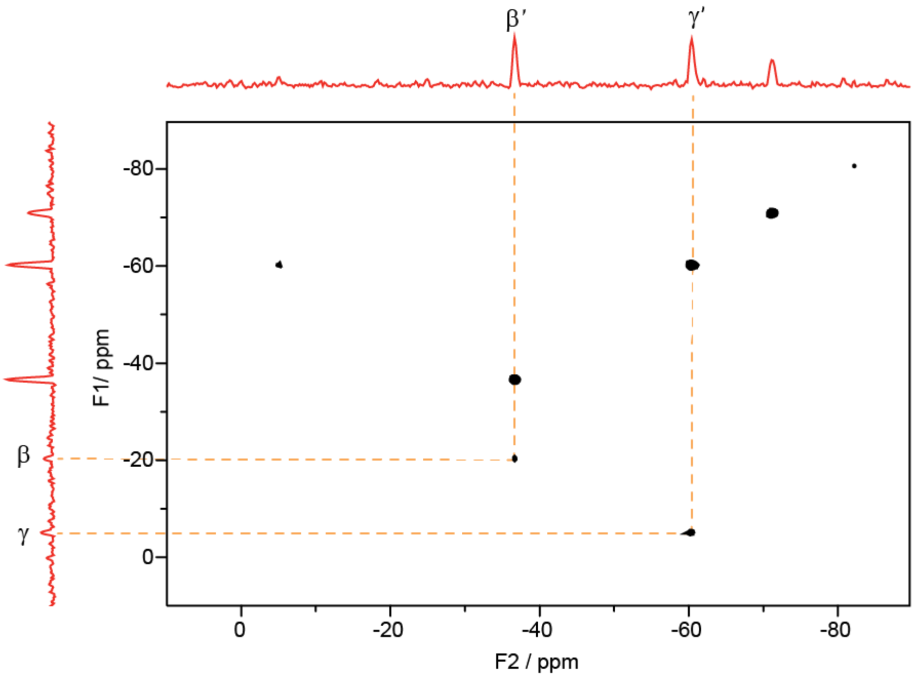

Figure S35. ^{31}P EXSY NMR spectrum of [**Eu.1**]$^{+}$ (6.57 mM) with ATP (6.57 mM). Conditions: 1:1 D_2O:MeOD, pD 7.0, 202.21 MHz. Mixing time = 5 ms.

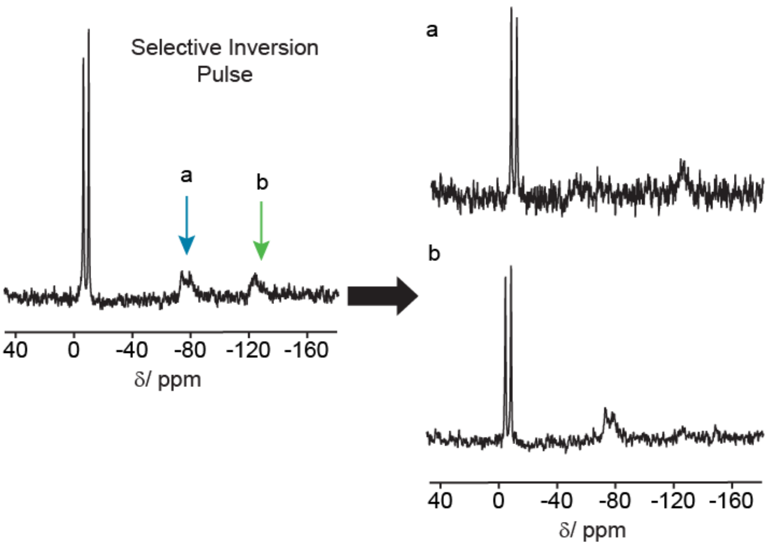

Figure S36A. Selective inversion ^{31}P NMR spectra of ADP (6.57 mM) and [**Eu.1**]$^{+}$ (6.57 mM) in 1:1 D_2O:MeOD, pD 7.0, 202.21 MHz.

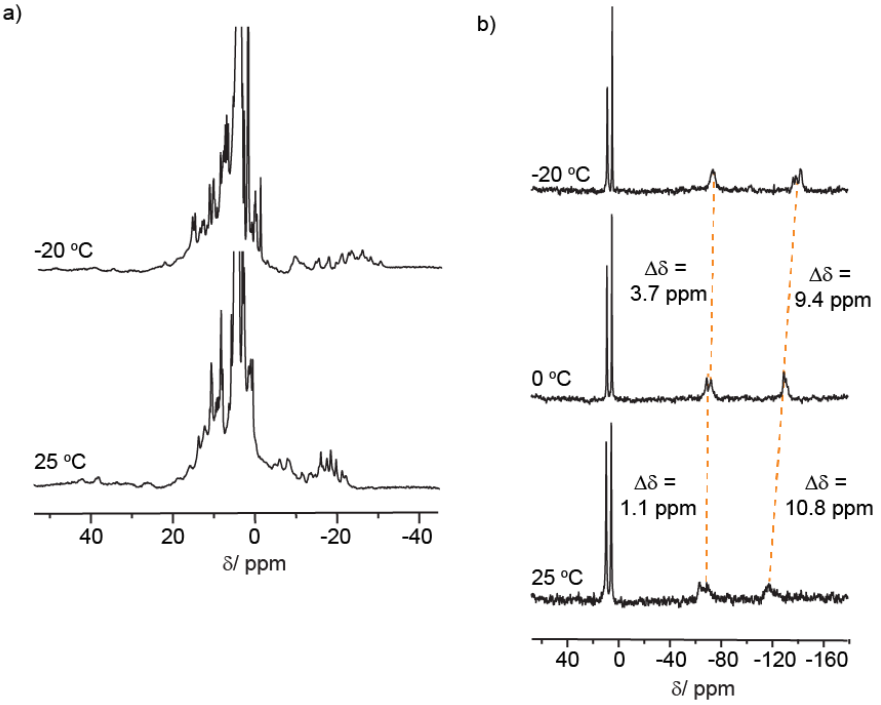

Figure S36B. ^{1}H (499.53 MHz) and ^{31}P NMR (202.21 MHz) of ADP (6.57 mM) and [**Eu.1**]$^{+}$ (6.57 mM) at different temperatures in 1:1 D_2O:MeOD, pD 7.0. a) ^{1}H NMR, b) ^{31}P NMR, showing the change in chemical shifts of the two bound ADP ^{31}P signals

2.8. Computational Methods

Density functional theory (DFT) calculations were performed with the hybrid meta-generalized gradient approximations (meta-GGA) TPSSh functional,[12] and the dispersion corrected B3LYP functional within the Gaussian 16 package (Revision E.01).[13] Both functionals have been previously used to model Ln(III) complexes, with the former suggested to provided more accurate geometries.[14–16] Considering that *f* orbitals do not play a major role in Eu−ligand bonds,[17] the large-core quasi-relativistic ECP (LCRECP), having 46 + $4f^n$ electrons, was used to describe the metal centre.[18] Calculations were therefore conducted on a pseudo singlet state configuration. LCRECP calculations have been shown to provide good results in DFT studies that focus on the structure, dynamics, and estimates of relative energies of Ln(III) complexes.[19–21] The 6-31G(d) basis set was used for all other atoms. Free energies were evaluated at 25 °C and corrected to a standard liquid state of 1 mol/L. In all cases, vibrational entropies were obtained using a quasi-harmonic approximation, treating vibrational modes below 100 cm^{-1} as free rotors and as rigid rotors above this cut-off, as first proposed by Grimme[22] and implemented in Python.[23] Solvent effects (water) were evaluated by using the polarizable continuum model (PCM) as implemented in Gaussian 16.

$[Eu.1]^+$ bound to water and formate. Using the crystallographic data for [**Eu.1**]$^+$, we modelled this complex bound to a single water molecule and to formate, which was present in the crystal structure (Fig. S37). Inclusion of solvent led to distances closer to those observed in the crystal structure. Upon optimisation, the formate anion appeared to bind more strongly to the metal centre compared with water, ($d_{Eu\text{-}o}$ = 2.37 vs 2.49 Å). It also indicates a weakening of the interaction between the metal centre and the four nitrogen atoms from the macrocyclic ring ($d_{Eu\text{-}N}$= 2.71 – 2.77 vs 2.67 Å for water-bound), with the distances between the metal centre and the two nitrogen atoms from the quinoline groups remaining almost constant. It is also apparent that, upon optimization, the hydrogen bonding interaction between one of the amide groups and formate is stronger ($d_{NH\text{--}O}$ = 1.87 vs 2.47 Å).

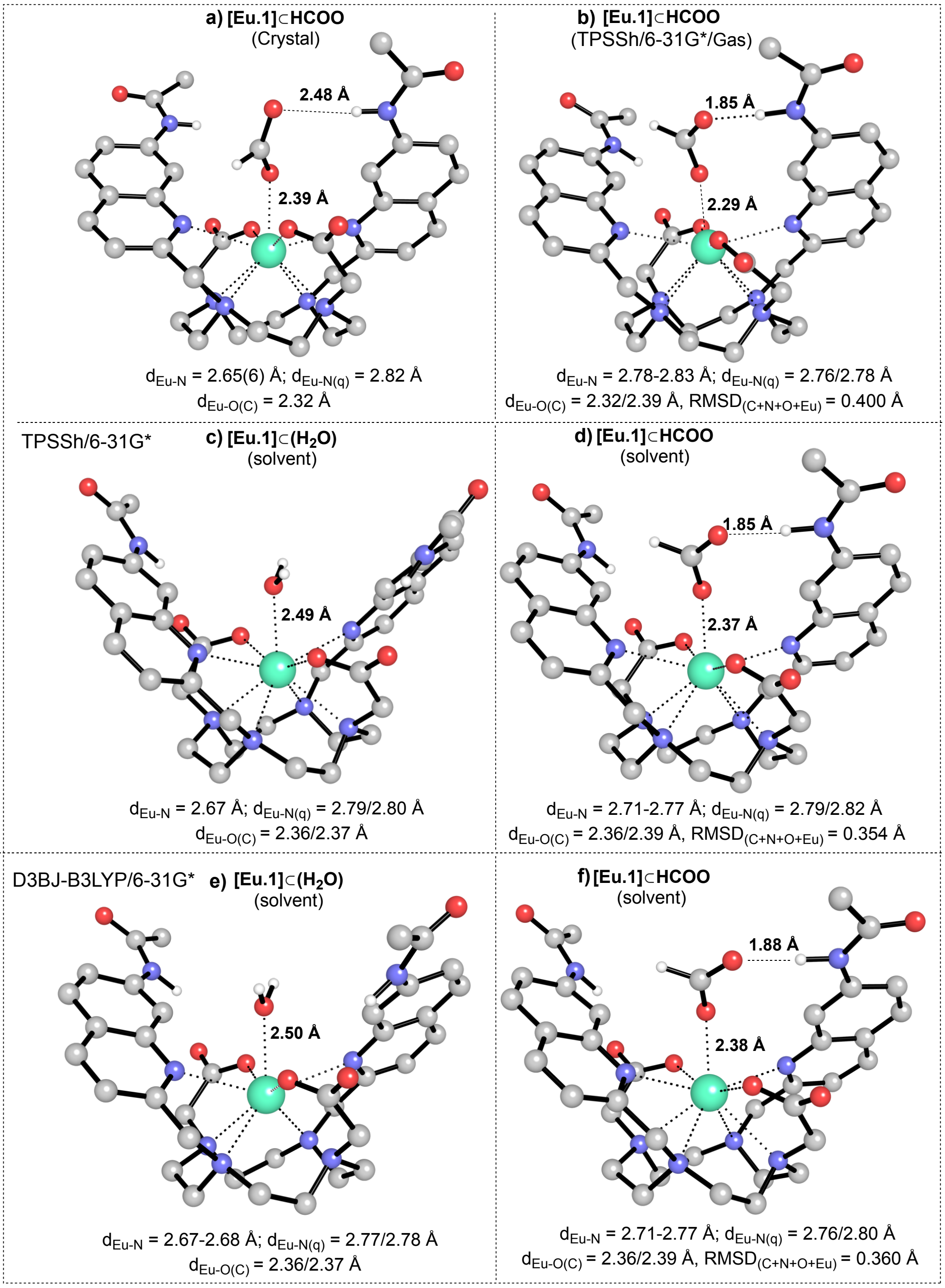

Figure S37. a) X-ray crystal structure of the [**Eu.1**]$^{+}$ ⊂HCOO complex. Geometries of the [**Eu.1**]$^{+}$⊂HCOO and [**Eu.1**]$^{+}$⊂H_2O complexes Optimised at the (b) TPSSh/6-31G* (c & d) PCM(H_2O)-TPSSh/6-31G* and (e & f) PCM(H_2O)-D3BJ-B3LYP/6-31G* level of theory. Hydrogens have been removed for clarity, except those that form H-bonds with the anion, for which distances are reported. Relevant distances between the metal centre and its first coordination shell are shown at the bottom of each figure [in Å].

[Eu.1]+ complex bound to nucleoside phosphate anions

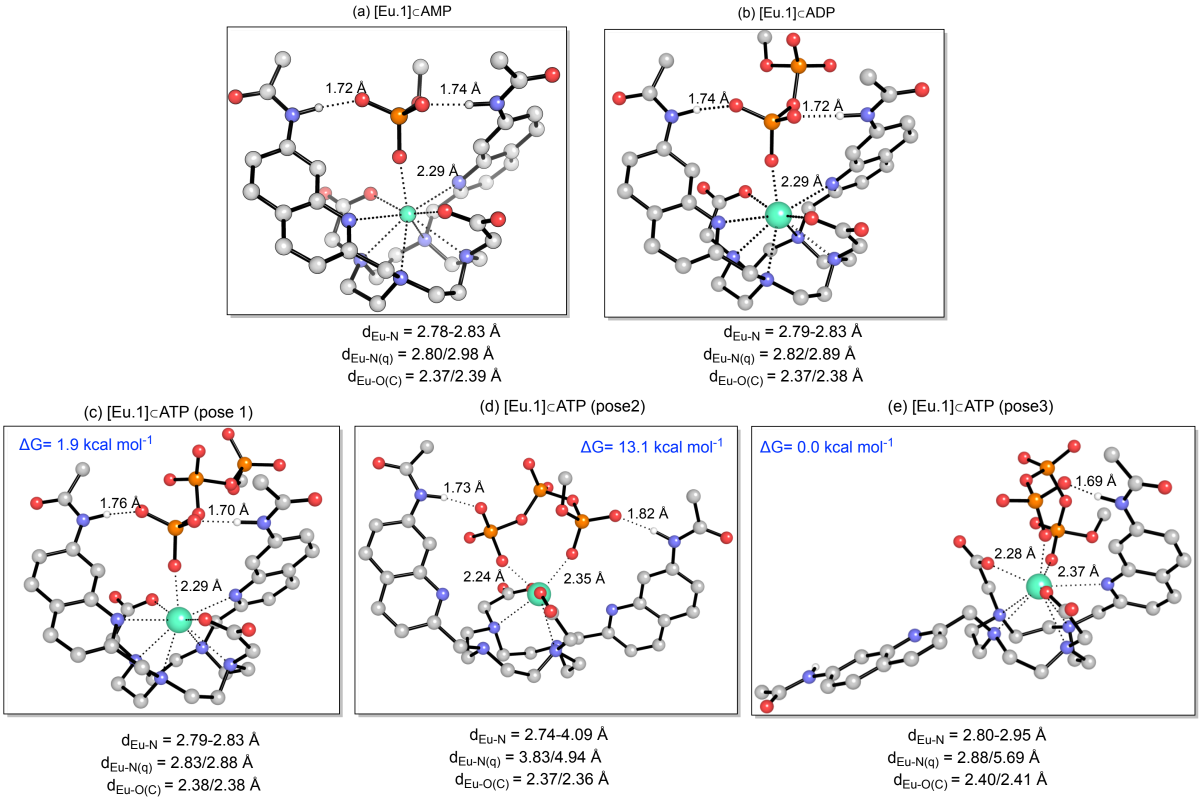

Figure S38. Optimised geometries, at the PCM(H_2O)-TPSSh/6-31G* level of theory, for the (a) [**Eu.1**]⊂AMP and (b) [**Eu.1**]⊂ADP monocoordinated. Three different binding modes were tested for the [**Eu.1**]⊂ATP complex: c) monocoordinated (pose1); (d) bicoordinated with quinoline groups moved away from the coordination shell (pose2) and (e)bicoordinated with only one quinoline group moved away (pose3). The relative energy of these species was quantified at the PCM(H_2O)-M06-2X/6-311+G**//PCM(H_2O)-TPSSh/6-31G* level of theory. Hydrogen atoms have been removed for clarity, except those that form H-bond with substrate, for which distances are reported. Relevant distances between the metal centre and its first coordination shell are shown at the bottom of each structure [in Å].

2.9. Mass spectral data of [Eu.1]$^+$ bound to ATP and ADP

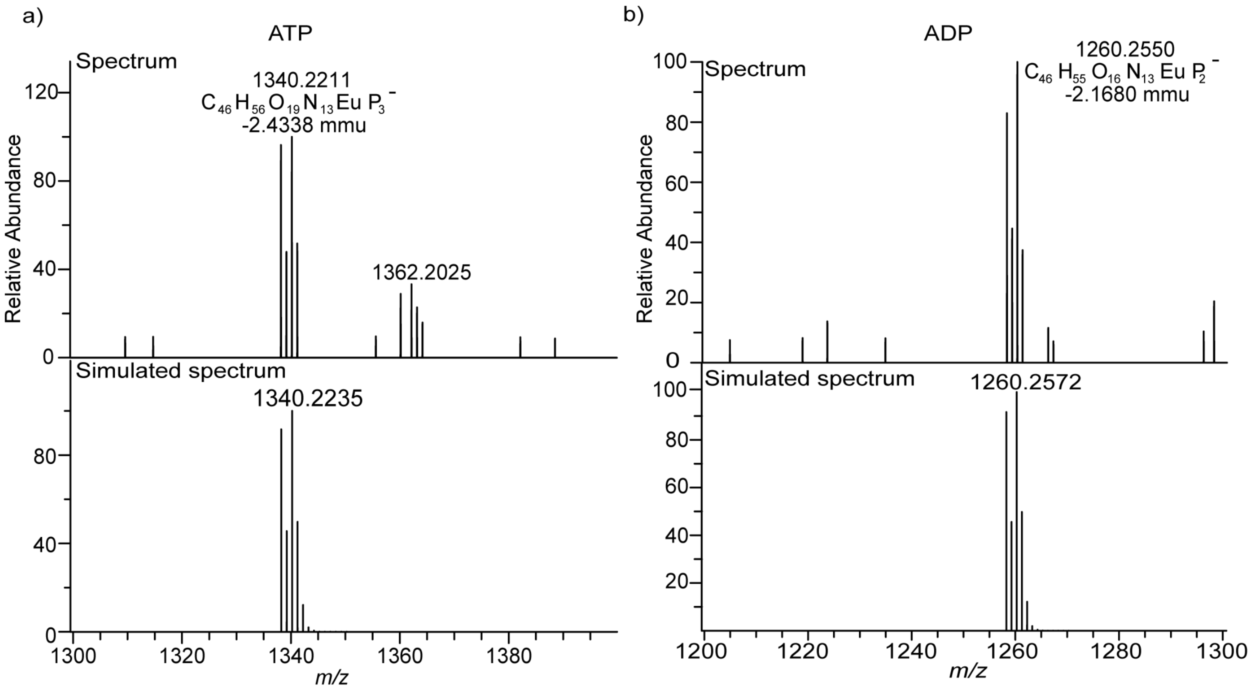

Figure S39: High resolution mass spectra of [**Eu.1**]$^+$ complexed to (a) ATP and (b) ADP, showing the formation of 1:1 adducts, in both cases.

2.10. Array data

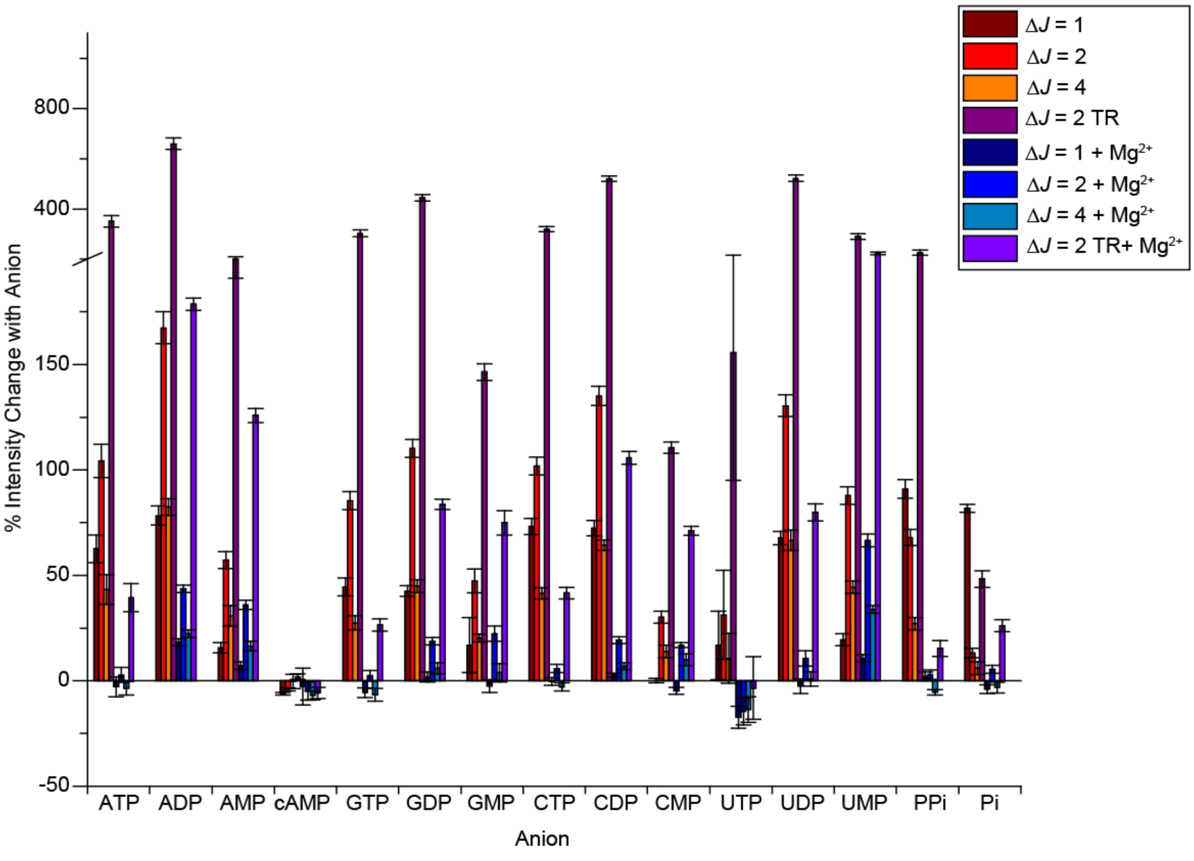

Figure S40. Percentage changes in emission intensity for [**Eu.1**]$^+$ (8 μM) for the three emission bands (ΔJ = 1 (570 – 600 nm), ΔJ = 2 (601 – 631 nm) and ΔJ = 4 (675 – 710 nm)) and the time-resolved ΔJ = 2 emission (615 – 625 nm) with and without $MgCl_2$ (5 mM) with various phosphoanions (1 mM) in 10 mM HEPES, pH 7.0, λ_{exc} = 330 nm

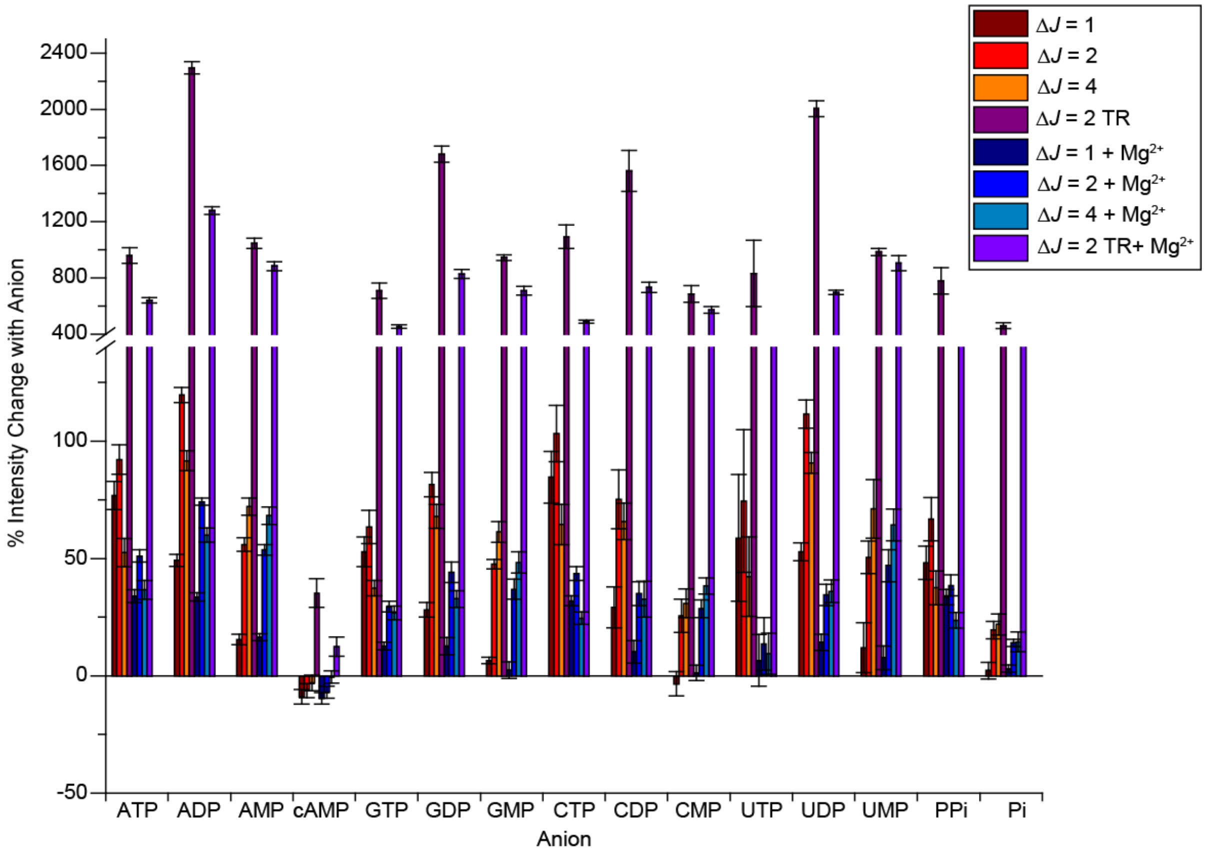

Figure S41. Percentage changes in emission intensity for [**Eu.2**]$^+$ (13 μM) for the three emission bands (ΔJ = 1 (570 – 600 nm), ΔJ = 2 (601 – 631 nm) and ΔJ = 4 (675 – 710 nm)) and the time-resolved ΔJ = 2 emission (615 – 625 nm) with and without $MgCl_2$ (5 mM) with various phosphoanions (1 mM) in 10 mM HEPES, pH 7.0, λ_{exc} = 330 nm

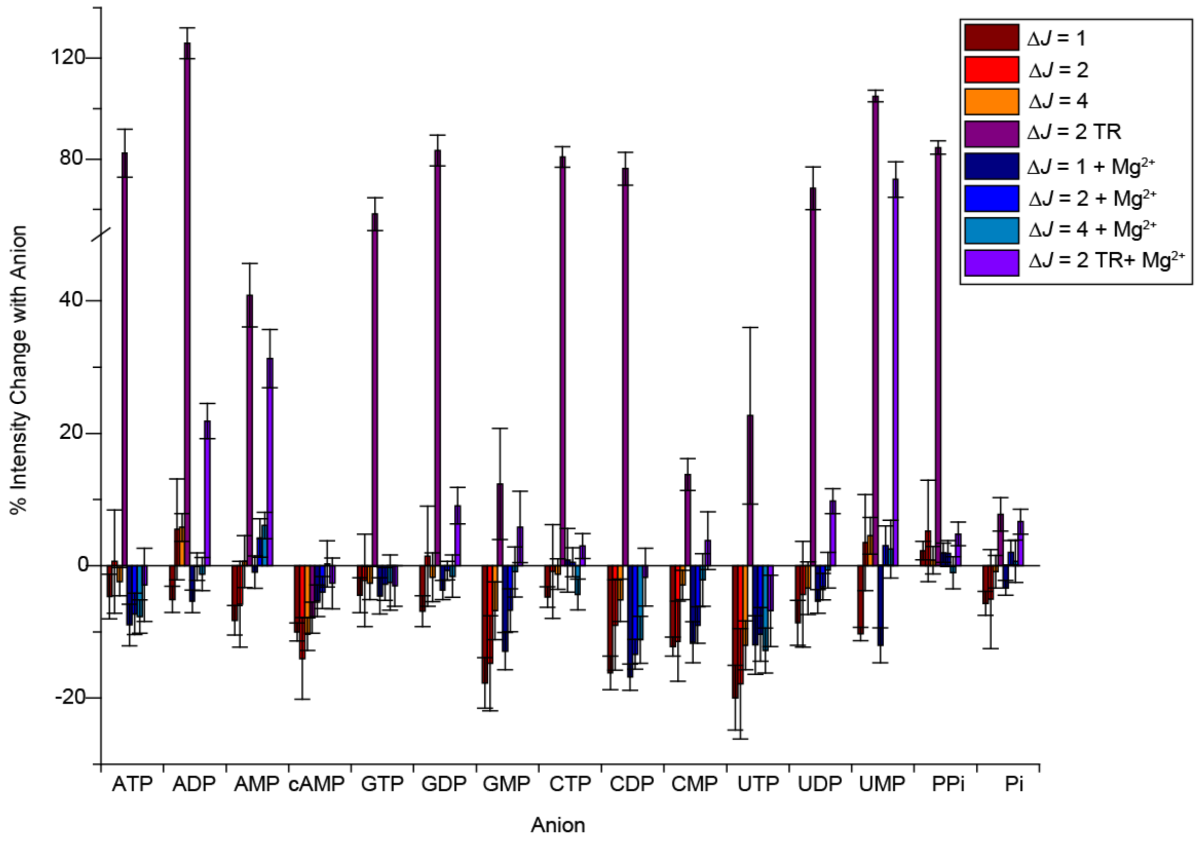

Figure S42. Percentage changes in emission intensity for [**Eu.4**]$^+$ (10 µM) for the three emission bands (ΔJ = 1 (570 – 600 nm), ΔJ = 2 (601 – 631 nm) and ΔJ = 4 (675 – 710 nm)) and the time-resolved ΔJ = 2 emission (615 – 625 nm) with and without $MgCl_2$ (5 mM) with various phosphoanions (1 mM) in 10 mM HEPES, pH 7.0, λ_{exc} = 330 nm

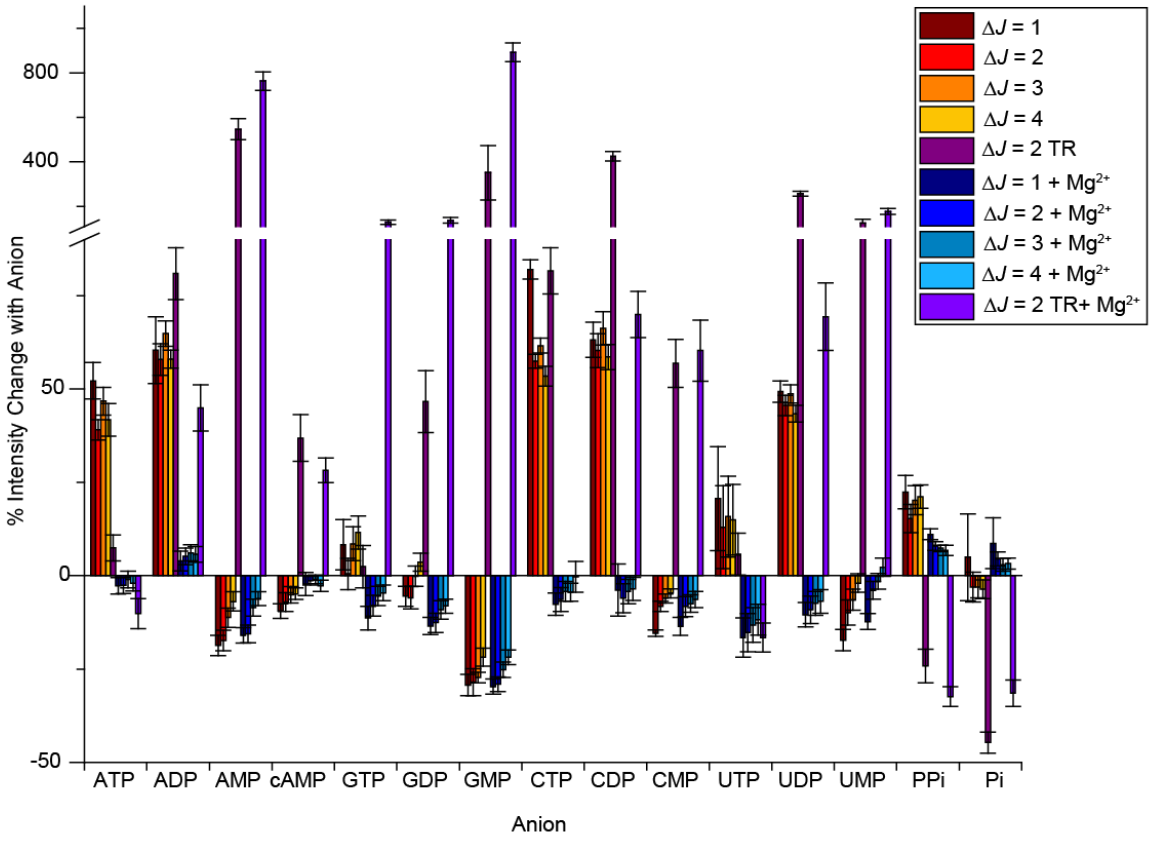

Figure S43. Percentage changes in emission intensity for [**Tb.1**]$^{+}$ (15 µM) for the four emission bands (ΔJ = 1 (479 – 507 nm), ΔJ = 2 (533 – 559 nm), ΔJ = 3 (576 – 598 nm) and ΔJ = 4 (611 – 633 nm)) and the time-resolved ΔJ = 2 emission (510 – 550 nm) with and without $MgCl_2$ (5 mM) with various phosphoanions (1 mM) in 10 mM HEPES, pH 7.0, λ_{exc} = 330 nm

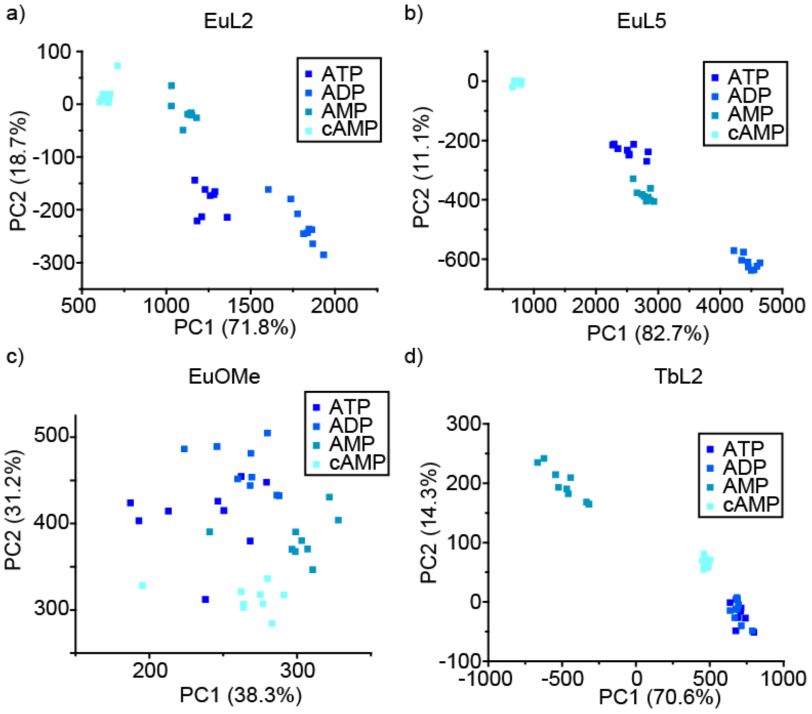

Figure S44. Principle component analysis score plot of % change in emission intensity for the individual complexes with ATP, ADP, AMP and cAMP (all 1 mM). a) [**Eu.1**]$^+$ (8 μM), b) [**Eu.2**]$^+$ (13 μM), c) [**Eu.4**]$^+$ (10 μM) and d) [**Tb.1**]$^+$ (15 μM). Conditions: 1 mM anion in 10 mM HEPES, pH 7.0

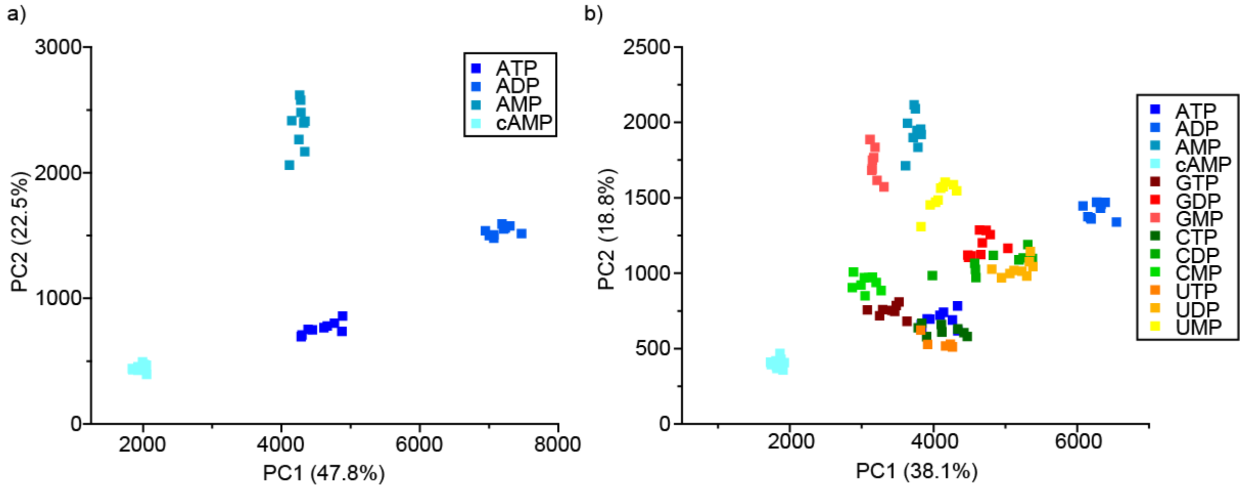

Figure S45. Principle component analysis score plot of % change in emission intensity for [**Eu.1**]$^+$ (8 μM), [**Eu.2**]$^+$ (13 μM), [**Eu.4**]$^+$ (10 μM) and [**Tb.1**]$^+$ (15 μM) with different anions, a) ATP, ADP, AMP, cAMP and Pi, b) The full series of nucleoside phosphate anions. Conditions: 1 mM anion in 10 mM HEPES, pH 7.0

3.0. Crystallographic Data Tables for $[Eu.1]^{+}·7H_2O$

Crystal data

$C_{37}H_{61}EuN_8O_{15}·7(H_2O)$	D_x = 1.654 Mg m^{-3}
M_r = 1009.89	Mo $K\alpha$ radiation, λ = 0.71073 Å
Orthorhombic, *Fddd*	Cell parameters from 25634 reflections
a = 18.1194 (3) Å	θ = 2.0–30.9°
b = 20.3557 (3) Å	μ = 1.63 mm^{-1}
c = 43.9834 (7) Å	T = 100 K
V = 16222.5 (4) Å^3	Prism, colourless
Z = 16	0.18 × 0.13 × 0.12 mm^3
F(000) = 8352	

Data collection

Rigaku FRE+ equipped with VHF Varimax confocal mirrors and an AFC12 goniometer and HyPix 6000 detector diffractometer	4666 independent reflections
Radiation source: Rotating-anode X-ray tube	4373 reflections with $I > 2\sigma(I)$
Confocal mirrors, VHF Varimax monochromator	R_{int} = 0.025
Detector resolution: 10 pixels mm^{-1}	θ_{max} = 27.5°, θ_{min} = 1.6°
profile data from ω–scans	h = -23→23
Absorption correction: gaussian *CrysAlis PRO* 1.171.39.46b (Rigaku Oxford Diffraction, 2018) Numerical absorption correction based on gaussian integration over a multifaceted crystal model. Empirical absorption correction using spherical harmonics, implemented in SCALE3 ABSPACK scaling algorithm.	k = -26→19
T_{min} = 0.319, T_{max} = 1.000	l = -56→57
46525 measured reflections	

Refinement

Refinement on F^2	Primary atom site location: iterative
Least-squares matrix: full	Secondary atom site location: difference Fourier map
$R[F^2 > 2\sigma(F^2)]$ = 0.046	Hydrogen site location: mixed
$wR(F^2)$ = 0.115	H atoms treated by a mixture of independent and constrained refinement
S = 1.30	$w = 1/[\sigma^2(F_o^2) + (0.0303P)^2 + 296.745P]$ where $P = (F_o^2 + 2F_c^2)/3$
4666 reflections	$(\Delta/\sigma)_{max}$ = 0.001
318 parameters	$\Delta\rangle_{max}$ = 1.48 e Å^{-3}

57 restraints	Δ〉min = -0.93 e Å^{-3}

Fractional atomic coordinates and isotropic or equivalent isotropic displacement parameters ($Å^2$) for ([Eu.1]$^{+}$·7H_2O*)*

	x	*y*	*z*	U_{iso}*/U_{eq}	Occ. (<1)
Eu1	0.375000	0.57903 (2)	0.375000	0.02350 (10)	
N1	0.4662 (2)	0.65879 (18)	0.34524 (8)	0.0251 (7)	
C1	0.5170 (3)	0.6915 (2)	0.36696 (10)	0.0279 (9)	
H1A	0.540547	0.729522	0.356852	0.034*	
H1B	0.556404	0.660315	0.372858	0.034*	
C2	0.4774 (3)	0.7147 (2)	0.39515 (11)	0.0295 (9)	
H2A	0.512624	0.738739	0.408281	0.035*	
H2B	0.437792	0.745689	0.389219	0.035*	
N2	0.4446 (2)	0.65957 (18)	0.41270 (8)	0.0254 (8)	
C3	0.3956 (2)	0.6890 (2)	0.43619 (10)	0.0277 (9)	
H3A	0.420701	0.727145	0.445580	0.033*	
H3B	0.386069	0.656237	0.452353	0.033*	
C4	0.3224 (3)	0.7112 (2)	0.42248 (11)	0.0291 (9)	
H4A	0.289581	0.726192	0.439071	0.035*	
H4B	0.331646	0.749189	0.408944	0.035*	
N4	0.2273 (4)	0.3782 (3)	0.30133 (11)	0.0638 (18)	
H4	0.225299	0.380195	0.321301	0.077*	
C5	0.5114 (2)	0.6176 (2)	0.32479 (10)	0.0285 (9)	
H5C	0.542356	0.587928	0.337264	0.034*	
H5D	0.544802	0.646309	0.312965	0.034*	
N3	0.4120 (2)	0.54182 (19)	0.31509 (8)	0.0271 (8)	
C6	0.4666 (3)	0.5770 (2)	0.30317 (10)	0.0276 (9)	
C7	0.4852 (3)	0.5778 (2)	0.27220 (11)	0.0306 (10)	
H7	0.525000	0.604022	0.265071	0.037*	
C8	0.4450 (3)	0.5401 (2)	0.25244 (10)	0.0304 (10)	
H8	0.455721	0.540779	0.231297	0.036*	
C9	0.3880 (2)	0.5005 (2)	0.26364 (10)	0.0246 (9)	
C10	0.3447 (3)	0.4593 (2)	0.24479 (10)	0.0278 (9)	
H10	0.353205	0.459325	0.223477	0.033*	
C11	0.2913 (3)	0.4198 (2)	0.25640 (10)	0.0295 (9)	
H11	0.262804	0.392544	0.243368	0.035*	
C12	0.2786 (3)	0.4198 (2)	0.28792 (11)	0.0330 (10)	
C13	0.3178 (3)	0.4606 (2)	0.30687 (10)	0.0322 (10)	
H13	0.306933	0.461060	0.328002	0.039*	

C14	0.3733 (3)	0.5015 (2)	0.29550 (10)	0.0249 (8)	
C15	0.1792 (4)	0.3347 (3)	0.28827 (14)	0.0468 (14)	
O1	0.1731 (2)	0.32659 (18)	0.26202 (9)	0.0418 (9)	
C16	0.1327 (6)	0.2966 (5)	0.31061 (18)	0.106 (4)	
H16A	0.085327	0.319054	0.313455	0.159*	
H16B	0.158528	0.293864	0.330146	0.159*	
H16C	0.123984	0.252259	0.302748	0.159*	
C17	0.5037 (2)	0.6215 (3)	0.42769 (10)	0.0300 (9)	
H17A	0.484087	0.602652	0.446804	0.036*	
H17B	0.544440	0.651693	0.433106	0.036*	
C18	0.5347 (2)	0.5657 (2)	0.40808 (10)	0.0277 (9)	
O2	0.49486 (19)	0.54675 (16)	0.38618 (7)	0.0308 (7)	
O3	0.59435 (19)	0.54171 (18)	0.41556 (8)	0.0366 (8)	
O4	0.375000	0.4617 (2)	0.375000	0.0520 (15)	
C19	0.3061 (9)	0.4306 (8)	0.3831 (5)	0.089 (4)	0.5
H19	0.281018	0.455025	0.398353	0.107*	0.5
O5	0.2663 (5)	0.375000	0.375000	0.082 (2)	
O6	0.0712 (3)	0.2265 (2)	0.24892 (11)	0.0553 (11)	
H6A	0.098 (4)	0.258 (3)	0.2534 (19)	0.083*	
H6B	0.089 (5)	0.199 (3)	0.2374 (16)	0.083*	
O7	0.125000	0.125000	0.2092 (2)	0.087 (3)	
O8	0.141 (3)	0.4237 (13)	0.3591 (7)	0.099 (7)	0.56 (7)
O8X	0.110 (3)	0.4205 (16)	0.3500 (11)	0.093 (8)	0.44 (7)
O9	-0.125000	0.375000	0.3555 (4)	0.074 (4)	0.75 (3)
O9X	-0.125000	0.375000	0.375000	0.040 (7)	0.50 (5)
O10	-0.0144 (12)	0.3800 (9)	0.3264 (8)	0.083 (8)	0.258 (14)
O10X	-0.0152 (15)	0.3659 (9)	0.3537 (9)	0.094 (8)	0.242 (14)

Geometric parameters (Å, º) for ([Eu.1]$^+$·7H_2O*)*

Eu1—O2^i	2.322 (3)	N3—C14	1.381 (6)
Eu1—O2	2.322 (3)	C6—C7	1.403 (6)
Eu1—O4	2.388 (5)	C7—C8	1.370 (7)
Eu1—N2^i	2.651 (4)	C7—H7	0.9500
Eu1—N2	2.651 (4)	C8—C9	1.399 (6)
Eu1—N1^i	2.660 (4)	C8—H8	0.9500
Eu1—N1	2.660 (4)	C9—C10	1.417 (6)
Eu1—N3	2.823 (4)	C9—C14	1.427 (6)
Eu1—N3^i	2.823 (4)	C10—C11	1.358 (7)
N1—C5	1.478 (6)	C10—H10	0.9500
N1—C1	1.484 (5)	C11—C12	1.406 (7)

N1—C4^{i}	1.495 (6)	C11—H11	0.9500
C1—C2	1.508 (6)	C12—C13	1.374 (7)
C1—H1A	0.9900	C13—C14	1.398 (6)
C1—H1B	0.9900	C13—H13	0.9500
C2—N2	1.486 (6)	C15—O1	1.172 (7)
C2—H2A	0.9900	C15—C16	1.509 (9)
C2—H2B	0.9900	C16—H16A	0.9800
N2—C17	1.477 (6)	C16—H16B	0.9800
N2—C3	1.488 (6)	C16—H16C	0.9800
C3—C4	1.525 (6)	C17—C18	1.532 (6)
C3—H3A	0.9900	C17—H17A	0.9900
C3—H3B	0.9900	C17—H17B	0.9900
C4—H4A	0.9900	C18—O3	1.232 (6)
C4—H4B	0.9900	C18—O2	1.264 (6)
N4—C15	1.368 (7)	O4—C19	1.444 (12)
N4—C12	1.389 (6)	O4—C19^{i}	1.444 (12)
N4—H4	0.8800	C19—O5	1.389 (12)
C5—C6	1.498 (6)	C19—H19	0.9500
C5—H5C	0.9900	O6—H6A	0.83 (2)
C5—H5D	0.9900	O6—H6B	0.83 (2)
N3—C6	1.330 (6)	O9—O9^{ii}	1.71 (4)
O2^{i}—Eu1—O2	147.11 (17)	H4A—C4—H4B	107.7
O2^{i}—Eu1—O4	73.56 (8)	C15—N4—C12	130.0 (5)
O2—Eu1—O4	73.56 (8)	C15—N4—H4	115.0
O2^{i}—Eu1—N2^{i}	66.28 (11)	C12—N4—H4	115.0
O2—Eu1—N2^{i}	138.80 (12)	N1—C5—C6	113.5 (4)
O4—Eu1—N2^{i}	128.21 (8)	N1—C5—H5C	108.9
O2^{i}—Eu1—N2	138.80 (11)	C6—C5—H5C	108.9
O2—Eu1—N2	66.28 (11)	N1—C5—H5D	108.9
O4—Eu1—N2	128.20 (8)	C6—C5—H5D	108.9
N2^{i}—Eu1—N2	103.59 (16)	H5C—C5—H5D	107.7
O2^{i}—Eu1—N1^{i}	72.31 (12)	C6—N3—C14	116.9 (4)
O2—Eu1—N1^{i}	130.49 (11)	C6—N3—Eu1	113.6 (3)
O4—Eu1—N1^{i}	127.61 (8)	C14—N3—Eu1	128.4 (3)
N2^{i}—Eu1—N1^{i}	68.59 (12)	N3—C6—C7	124.6 (4)
N2—Eu1—N1^{i}	67.05 (11)	N3—C6—C5	116.7 (4)
O2^{i}—Eu1—N1	130.49 (11)	C7—C6—C5	118.7 (4)
O2—Eu1—N1	72.31 (12)	C8—C7—C6	118.8 (4)
O4—Eu1—N1	127.61 (8)	C8—C7—H7	120.6

N2^{i}—Eu1—N1	67.05 (11)	C6—C7—H7	120.6
N2—Eu1—N1	68.60 (12)	C7—C8—C9	119.5 (4)
N1^{i}—Eu1—N1	104.79 (17)	C7—C8—H8	120.3
O2^{i}—Eu1—N3	87.04 (11)	C9—C8—H8	120.3
O2—Eu1—N3	84.24 (11)	C8—C9—C10	122.9 (4)
O4—Eu1—N3	74.43 (8)	C8—C9—C14	118.5 (4)
N2^{i}—Eu1—N3	72.24 (11)	C10—C9—C14	118.6 (4)
N2—Eu1—N3	129.57 (11)	C11—C10—C9	121.7 (4)
N1^{i}—Eu1—N3	140.40 (11)	C11—C10—H10	119.1
N1—Eu1—N3	63.70 (11)	C9—C10—H10	119.1
O2^{i}—Eu1—N3^{i}	84.24 (11)	C10—C11—C12	119.2 (4)
O2—Eu1—N3^{i}	87.04 (11)	C10—C11—H11	120.4
O4—Eu1—N3^{i}	74.43 (8)	C12—C11—H11	120.4
N2^{i}—Eu1—N3^{i}	129.57 (11)	C13—C12—N4	117.2 (4)
N2—Eu1—N3^{i}	72.24 (11)	C13—C12—C11	120.9 (4)
N1^{i}—Eu1—N3^{i}	63.70 (11)	N4—C12—C11	121.9 (4)
N1—Eu1—N3^{i}	140.41 (11)	C12—C13—C14	121.0 (4)
N3—Eu1—N3^{i}	148.86 (15)	C12—C13—H13	119.5
C5—N1—C1	107.6 (3)	C14—C13—H13	119.5
C5—N1—C4^{i}	110.3 (4)	N3—C14—C13	119.7 (4)
C1—N1—C4^{i}	107.8 (3)	N3—C14—C9	121.7 (4)
C5—N1—Eu1	107.3 (2)	C13—C14—C9	118.6 (4)
C1—N1—Eu1	110.0 (3)	O1—C15—N4	124.4 (5)
C4^{i}—N1—Eu1	113.7 (3)	O1—C15—C16	121.1 (6)
N1—C1—C2	112.0 (4)	N4—C15—C16	114.5 (6)
N1—C1—H1A	109.2	C15—C16—H16A	109.5
C2—C1—H1A	109.2	C15—C16—H16B	109.5
N1—C1—H1B	109.2	H16A—C16—H16B	109.5
C2—C1—H1B	109.2	C15—C16—H16C	109.5
H1A—C1—H1B	107.9	H16A—C16—H16C	109.5
N2—C2—C1	112.3 (4)	H16B—C16—H16C	109.5
N2—C2—H2A	109.1	N2—C17—C18	113.8 (4)
C1—C2—H2A	109.1	N2—C17—H17A	108.8
N2—C2—H2B	109.1	C18—C17—H17A	108.8
C1—C2—H2B	109.1	N2—C17—H17B	108.8
H2A—C2—H2B	107.9	C18—C17—H17B	108.8
C17—N2—C2	109.8 (4)	H17A—C17—H17B	107.7
C17—N2—C3	109.5 (3)	O3—C18—O2	125.7 (4)
C2—N2—C3	107.2 (3)	O3—C18—C17	117.7 (4)
C17—N2—Eu1	107.4 (3)	O2—C18—C17	116.5 (4)

C2—N2—Eu1	109.4 (3)	O3—C18—Eu1	159.1 (4)
C3—N2—Eu1	113.6 (3)	O2—C18—Eu1	34.5 (2)
N2—C3—C4	111.3 (4)	C17—C18—Eu1	82.2 (2)
N2—C3—H3A	109.4	C18—O2—Eu1	127.5 (3)
C4—C3—H3A	109.4	C19—O4—C19^{i}	128.0 (16)
N2—C3—H3B	109.4	C19—O4—Eu1	116.0 (8)
C4—C3—H3B	109.4	C19^{i}—O4—Eu1	116.0 (8)
H3A—C3—H3B	108.0	O5—C19—O4	138.0 (17)
N1^{i}—C4—C3	113.7 (4)	O5—C19—H19	111.0
N1^{i}—C4—H4A	108.8	O4—C19—H19	111.0
C3—C4—H4A	108.8	C19—O5—C19^{iii}	117.3 (18)
N1^{i}—C4—H4B	108.8	H6A—O6—H6B	116 (8)
C3—C4—H4B	108.8		
C5—N1—C1—C2	-159.6 (4)	C15—N4—C12—C11	-3.5 (11)
C4^{i}—N1—C1—C2	81.3 (4)	C10—C11—C12—C13	2.2 (8)
Eu1—N1—C1—C2	-43.1 (4)	C10—C11—C12—N4	-176.9 (5)
N1—C1—C2—N2	62.6 (5)	N4—C12—C13—C14	176.6 (5)
C1—C2—N2—C17	71.9 (5)	C11—C12—C13—C14	-2.5 (8)
C1—C2—N2—C3	-169.3 (4)	C6—N3—C14—C13	176.6 (4)
C1—C2—N2—Eu1	-45.8 (4)	Eu1—N3—C14—C13	-16.3 (6)
C17—N2—C3—C4	-163.2 (4)	C6—N3—C14—C9	-3.8 (6)
C2—N2—C3—C4	77.8 (4)	Eu1—N3—C14—C9	163.3 (3)
Eu1—N2—C3—C4	-43.2 (4)	C12—C13—C14—N3	-179.7 (5)
N2—C3—C4—N1^{i}	52.8 (5)	C12—C13—C14—C9	0.7 (7)
C1—N1—C5—C6	175.2 (4)	C8—C9—C14—N3	2.2 (6)
C4^{i}—N1—C5—C6	-67.4 (5)	C10—C9—C14—N3	-178.2 (4)
Eu1—N1—C5—C6	56.9 (4)	C8—C9—C14—C13	-178.2 (4)
C14—N3—C6—C7	2.7 (7)	C10—C9—C14—C13	1.4 (6)
Eu1—N3—C6—C7	-166.3 (4)	C12—N4—C15—O1	-1.7 (13)
C14—N3—C6—C5	-176.4 (4)	C12—N4—C15—C16	178.6 (8)
Eu1—N3—C6—C5	14.6 (5)	C2—N2—C17—C18	-89.0 (5)
N1—C5—C6—N3	-50.1 (6)	C3—N2—C17—C18	153.7 (4)
N1—C5—C6—C7	130.8 (4)	Eu1—N2—C17—C18	29.9 (4)
N3—C6—C7—C8	0.1 (7)	N2—C17—C18—O3	163.4 (4)
C5—C6—C7—C8	179.1 (4)	N2—C17—C18—O2	-19.2 (6)
C6—C7—C8—C9	-1.8 (7)	N2—C17—C18—Eu1	-23.1 (3)
C7—C8—C9—C10	-178.9 (4)	O3—C18—O2—Eu1	170.3 (3)
C7—C8—C9—C14	0.6 (7)	C17—C18—O2—Eu1	-6.8 (6)
C8—C9—C10—C11	177.8 (4)	C19^{i}—O4—C19—O5	-35.7 (17)

C14—C9—C10—C11	-1.7 (7)	Eu1—O4—C19—O5	144.3 (17)
C9—C10—C11—C12	0.0 (7)	O4—C19—O5—C19^{iii}	33.4 (16)
C15—N4—C12—C13	177.4 (7)		

Symmetry codes: (i) -*x*+3/4, *y*, -*z*+3/4; (ii) -*x*-1/4, *y*, -*z*+3/4; (iii) *x*, -*y*+3/4, -*z*+3/4.

Document origin: *publCIF* [Westrip, S. P. (2010). *J. Apply. Cryst.*, **43**, 920-925].

Computing details

Data collection: *CrysAlis PRO* 1.171.39.46b (Rigaku OD, 2018); cell refinement: *CrysAlis PRO* 1.171.39.46b (Rigaku OD, 2018); data reduction: *CrysAlis PRO* 1.171.39.46b (Rigaku OD, 2018); program(s) used to solve structure: SHELXT 2014/5 (Sheldrick, 2014); program(s) used to refine structure: *SHELXL2018*/1 (Sheldrick, 2018); molecular graphics: Bruker *SHELXTL*; software used to prepare material for publication: Bruker *SHELXTL*.

Special details

Geometry. All esds (except the esd in the dihedral angle between two l.s. planes) are estimated using the full covariance matrix. The cell esds are taken into account individually in the estimation of esds in distances, angles and torsion angles; correlations between esds in cell parameters are only used when they are defined by crystal symmetry. An approximate (isotropic) treatment of cell esds is used for estimating esds involving l.s. planes.

Table 1

Experimental details

Crystal data	
Chemical formula	$C_{37}H_{61}EuN_8O_{15}\cdot 7(H_2O)$
M_r	1009.89
Crystal system, space group	Orthorhombic, *Fddd*
Temperature (K)	100
a, *b*, *c* (Å)	18.1194 (3), 20.3557 (3), 43.9834 (7)
V (Å^3)	16222.5 (4)
Z	16
Radiation type	Mo *K*α
μ (mm^{-1})	1.63
Crystal size (mm^3)	0.18 × 0.13 × 0.12
Data collection	
Diffractometer	Rigaku FRE+ equipped with VHF Varimax confocal mirrors and an AFC12 goniometer and HyPix 6000 detector diffractometer
Absorption correction	Gaussian *CrysAlis PRO* 1.171.39.46b (Rigaku Oxford Diffraction, 2018) Numerical absorption correction based on gaussian integration over a

	multifaceted crystal model Empirical absorption correction using spherical harmonics, implemented in SCALE3 ABSPACK scaling algorithm.
T_{min}, T_{max}	0.319, 1.000
No. of measured, independent and observed [$I > 2\sigma(I)$] reflections	46525, 4666, 4373
R_{int}	0.025
(sin θ/λ)$_{max}$ (Å^{-1})	0.649
Refinement	
$R[F^2 > 2\sigma(F^2)]$, $wR(F^2)$, S	0.046, 0.115, 1.30
No. of reflections	4666
No. of parameters	318
No. of restraints	57
H-atom treatment	H atoms treated by a mixture of independent and constrained refinement
	$w = 1/[\sigma^2(F_o^2) + (0.0303P)^2 + 296.745P]$ where $P = (F_o^2 + 2F_c^2)/3$
$\Delta\rangle_{max}$, $\Delta\rangle_{min}$ (e Å^{-3})	1.48, -0.93

Computer programs: *CrysAlis PRO* 1.171.39.46b (Rigaku OD, 2018), SHELXT 2014/5 (Sheldrick, 2014), *SHELXL2018*/1 (Sheldrick, 2018), Bruker *SHELXTL*.

Table 2

Hydrogen-bond geometry (Å, º) for ($[Eu.1]^+\cdot 7H_2O$*)*

D—H···*A*	*D*—H	H···*A*	*D*···*A*	*D*—H···*A*
C2—H2*A*···O8*X*^{i}	0.99	2.58	3.36 (4)	135
C3—H3*B*···N3^{ii}	0.99	2.60	3.173 (6)	117
N4—H4···O5	0.88	2.48	3.317 (5)	160
N4—H4···O8	0.88	2.43	3.13 (3)	137
N4—H4···O8*X*	0.88	2.58	3.14 (4)	122
C5—H5*C*···O2	0.99	2.46	3.075 (6)	120
C11—H11···O1	0.95	2.26	2.871 (6)	121
C13—H13···O2^{ii}	0.95	2.53	3.261 (6)	134
C13—H13···O4	0.95	2.41	3.171 (5)	137
C16—H16*A*···O10	0.98	2.26	3.23 (2)	170
C16—H16*C*···O6	0.98	2.61	3.263 (9)	124
C17—H17*B*···O3^{iii}	0.99	2.45	3.315 (6)	146
O6—H6*A*···O1	0.83 (2)	1.99 (2)	2.810 (5)	173 (9)
O6—H6*B*···O7	0.83 (2)	2.05 (2)	2.876 (7)	175 (9)

Symmetry codes: (i) *x*+1/2, -*y*+5/4, -*z*+3/4; (ii) -*x*+3/4, *y*, -*z*+3/4; (iii) -*x*+5/4, -*y*+5/4, *z*.

References

1 S. J. Butler, *Chem. Commun.*, 2015, **51**, 10879–10882.

2 R. Mailhot, T. Traviss-Pollard, R. Pal and S. J. Butler, *Chem. - A Eur. J.*, 2018, **24**, 10745–10755.

3 M. Harris, L. Vander Elst, S. Laurent and T. N. Parac-Vogt, *Dalt. Trans.*, 2016, **45**, 4791–4801.

4 S. Karthik, A. Jana, B. Saha, B. K. Kalyani, S. K. Ghosh, Y. Zhao and N. D. P. Singh, *J. Mater. Chem. B*, 2014, **2**, 7971–7977.

5 C. Wang, X. Yang, S. Wang, T. Zhu, L. Bo, L. Zhang, H. Chen, D. Jiang, X. Dong and S. Huang, *J. Mater. Chem. C*, 2018, **6**, 865–874.

6 D. Maffeo and J. A. G. Williams, *Inorganica Chim. Acta*, 2003, **355**, 127–136.

7 C. Breen, R. Pal, M. R. J. Elsegood, S. Teat, F. Iza, K. Wende, B. Buckley and S. J. Butler, *Chem. Sci.*, 2020, DOI:10.1039/C9SC06053G.

8 B. B. Bleaney, C. M. Dobson, B. A. Levine, R. B. Martin, R. J. P Williams and A. V Xavier, *Origin of Lanthanide Nuclear Magnetic Resonance Shifts and Their Uses*, 1972.

9 A. Beeby, I. M. Clarkson, R. S. Dickins, S. Faulkner, D. Parker, L. Royle, A. S. De Sousa, J. A. G. Williams and M. Woods, *J. Chem. Soc. Perkin Trans. 2*, 1999, 493–503.

10 G. M. Sheldrick, *Acta Crystallogr. Sect. A Struct. Chem.*, 2015, **71**, 3–8.

11 G. M. Sheldrick, *Acta Crystallogr. Sect. C Struct. Chem.*, 2015, **71**, 3–8.

12 J. Tao, J. P. Perdew, V. N. Staroverov and G. E. Scuseria, 2003, **91**, 146401.

13 M. . Frisch and E. Al., *Gaussian09*, Wallingford CT., 01 edn., 2009.

14 M. Regueiro-Figueroa, D. Esteban-Gõmez, A. De Blas, T. Rodríguez-Blas and C. Platas-Iglesias, *Chem. - A Eur. J.*, 2014, **20**, 3974–3981.

15 A. Roca-Sabio, M. Regueiro-Figueroa, D. Esteban-Gómez, A. de Blas, T. Rodríguez-Blas and C. Platas-Iglesias, *Comput. Theor. Chem.*, 2012, **999**, 93–104.

16 N. C. Cakić, B. Tickner, M. Zaiss, D. Esteban-Gómezgómez, C. Platas-Iglesias, G. Angelovski, † Mr and N. Agents, *Inorg. Chem.*, 2017, **56**, 7737–7745.

17 L. Maron and O. Eisenstein, *J. Phys. Chem. A*, 2000, **104**, 7140–7143.

18 M. Dolg, H. Stoll, A. Savin and H. Preuss, *Theor. Chim. Acta*, 1989, **75**, 173–194.

19 O. Eisenstein and L. Maron, *J. Organometalic Chem.*, 2002, **647**, 190–197.

20 C. Platas-Iglesias, *Eur. J. Inorg. Chem.*, 2011, 2012, 2023–2033.

21 M. Dolg, X. Cao and J. Ciupka, *J. Electron Spectros. Relat. Phenomena*, 2014, **194**, 8–13.

22 S. Grimme, *Chem. - A Eur. J.*, 2012, **18**, 9955–9964.

23 I.P. Funes-Ardois, GoodVibes: GoodVibes v1.0.1, 2016, doi:http://dx.doi.org/10.5281/zenodo.60811. (accessed 7 March 2017); Available from: http://paton.chem.ox.ac.uk/software/software.html.
